# Genome sequence of the agarwood tree *Aquilaria sinensis* (Lour.) Spreng: the first chromosome-level draft genome in the Thymelaeceae family

**DOI:** 10.1093/gigascience/giaa013

**Published:** 2020-03-02

**Authors:** Xupo Ding, Wenli Mei, Qiang Lin, Hao Wang, Jun Wang, Shiqing Peng, Huiliang Li, Jiahong Zhu, Wei Li, Pei Wang, Huiqin Chen, Wenhua Dong, Dong Guo, Caihong Cai, Shengzhuo Huang, Peng Cui, Haofu Dai

**Affiliations:** 1 Hainan Engineering Research Center of Agarwood, Institute of Tropical Bioscience and Biotechnology, Chinese Academy of Tropical Agricultural Sciences, Rd. Xueyuan No. 4, Haikou 571101, China; 2 Guangdong Laboratory of Lingnan Modern Agriculture, Shenzhen; Genome Analysis Laboratory of the Ministry of Agriculture; Agricultural Genomics Institute at Shenzhen, Chinese Academy of Agricultural Sciences, Rd. Pengfei No. 7, Shenzhen 518120, China; 3 Key Laboratory of Biology and Genetic Resources of Tropical Crops of Ministry of Agriculture and Rural Affairs, Institute of Tropical Bioscience and Biotechnology; Chinese Academy of Tropical Agriculture Sciences, Rd. Xueyuan No. 4, Haikou 571101, China

**Keywords:** *Aquilaria sinensis*, agarwood, chromosome-level genome assembly, Hi-C sequencing, annotation

## Abstract

**Backgroud:**

*Aquilaria sinensis* (Lour.) Spreng is one of the important plant resources involved in the production of agarwood in China. The agarwood resin collected from wounded *Aquilaria* trees has been used in Asia for aromatic or medicinal purposes from ancient times, although the mechanism underlying the formation of agarwood still remains poorly understood owing to a lack of accurate and high-quality genetic information.

**Findings:**

We report the genomic architecture of *A. sinensis* by using an integrated strategy combining Nanopore, Illumina, and Hi-C sequencing. The final genome was ∼726.5 Mb in size, which reached a high level of continuity and a contig N50 of 1.1 Mb. We combined Hi-C data with the genome assembly to generate chromosome-level scaffolds. Eight super-scaffolds corresponding to the 8 chromosomes were assembled to a final size of 716.6 Mb, with a scaffold N50 of 88.78 Mb using 1,862 contigs. BUSCO evaluation reveals that the genome completeness reached 95.27%. The repeat sequences accounted for 59.13%, and 29,203 protein-coding genes were annotated in the genome. According to phylogenetic analysis using single-copy orthologous genes, we found that *A. sinensis* is closely related to *Gossypium hirsutum* and *Theobroma cacao* from the Malvales order, and *A. sinensis* diverged from their common ancestor ∼53.18–84.37 million years ago.

**Conclusions:**

Here, we present the first chromosome-level genome assembly and gene annotation of *A. sinensis*. This study should contribute to valuable genetic resources for further research on the agarwood formation mechanism, genome-assisted improvement, and conservation biology of *Aquilaria* species.

## Background

Agarwood is the fragrant resin-filled heartwood from the trees of the *Aquilaria* or *Gyrinops* genus, high-quality preparations of which are more costly than gold in the international market [1, 2]. Agarwood has been used as precious incense in Buddhist, Islamic, and Hindu ceremonies, and also as a traditional medicine in Chinese therapies and Ayurveda [3]. Modern pharmacological and chemical studies have indicated that sesquiterpenoid and phenylethyl chromone derivatives are the principal compounds in agarwood, many of which have been studied for potential pharmacological activities including neuroprotective, sedative, acetylcholinesterase inhibitory, antioxidant, antibacterial, and anti-inflammatory activities [4–7]. However, healthy *Aquilaria* trees generate very little agarwood unless they have been stimulated by various forms of injury or microbial infestation. In the wild, agarwood formation is usually related to natural factors such as wounding by wind or lightning damage, or gnawing by insects and fungi. [8, 9]. As a result of agarwood's potential medicinal and economic importance, traditional methods used for producing agarwood in Asia include chopping, nailing, boring holes, burning the stem of *Aquilaria* trees, or pruning the partial trunk [10]. This has resulted in wild *Aquilaria* plants being excessively exploited, and many species are now decreasing or endangered [11].

**Figure 1:**
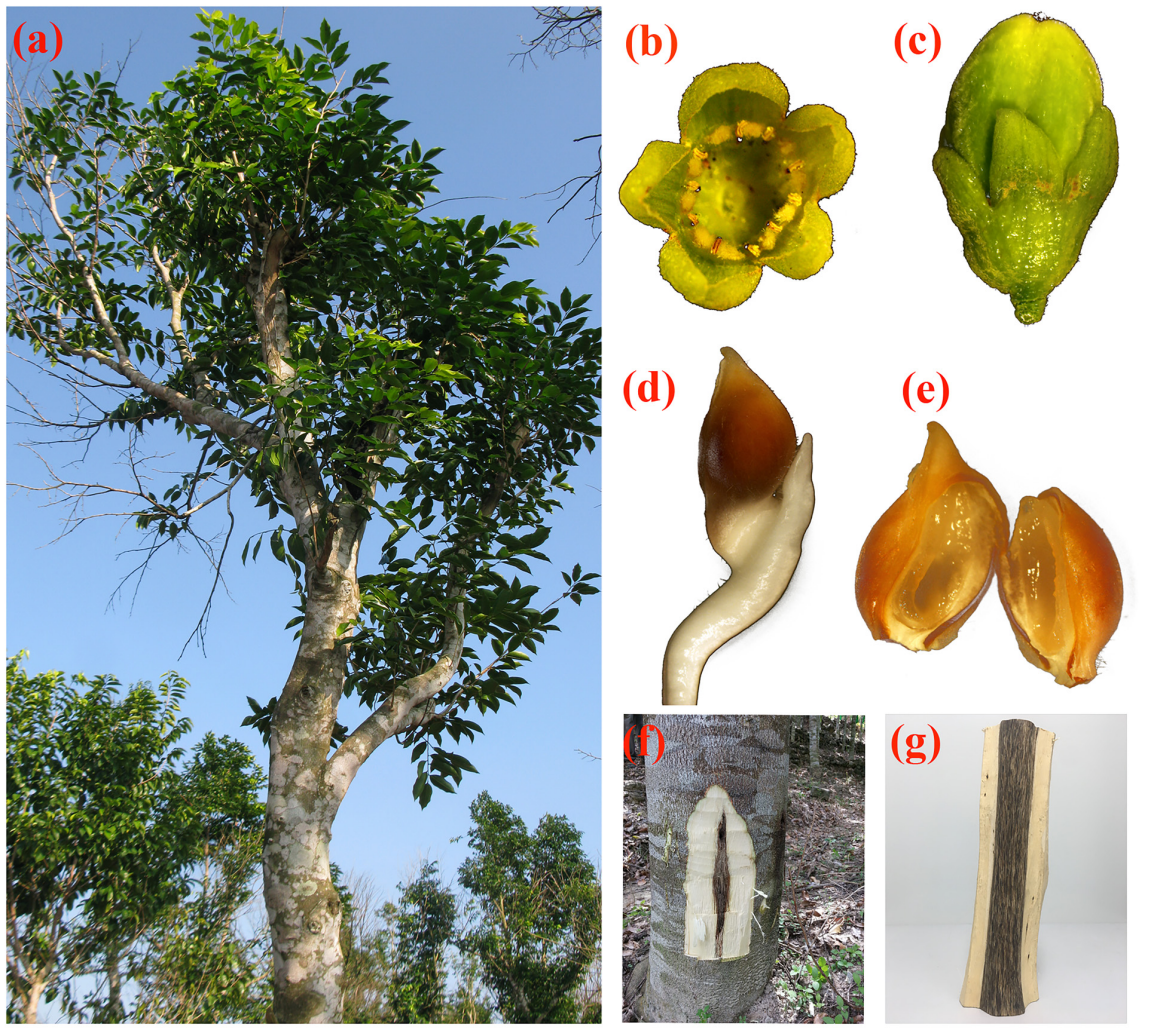
Morphological characteristics of *Aquilaria sinensis*. (a) mature tree; (b) flower; (c) fruit; (d) seed; (e) cracked seed; (f) agarwood generation; (g) agarwood. The images b–e were captured using a stereoscopic fluorescence microscope (Olympus SZX16, Pittsburgh, PA) under the dark field. All the photos were taken by Dr. Jun Wang and processed by Dr. Xupo Ding.

*Aquilaria sinensis* has been harvested and cultured for producing agarwood, which has been used in traditional Chinese medicine in China as early as the seventh century [11]. The morphological characteristics and agarwood of *A. sinensis* are shown in Fig. 1. As the largest producer of agarwood in China, the population of *A. sinensis* has undergone a dramatic decline in the past decade and its wild populations are threatened [11, 12]. The availability of agarwood is limited by the exhaustion of its time-consuming preparation and its plant sources. Although the expression of genes related to terpene synthesis or stress responses during agarwood formation has been described via transcriptome sequencing [2, 13, 14], the molecular mechanism of agarwood formation has remained unclear because of a lack of accurate genome information and genetic resources. Recently it has been discovered that 2-(2-phenylethyl) chromone and its derivatives were the key markers for agarwood formation in *A. sinensis* and their hypothetical biosynthetic pathway has been elucidated [8]. With the decreasing population of *A. sinensis* plants in the wild and increasing demand in the agarwood market, it is important to interrogate the genomic background to explore the mechanism of agarwood formation and to accelerate genome-assisted improvement in breeding systems.

Herein, we sequenced and assembled the genome of *Aquilaria sinensis* (NCBI:txid210372) by means of a hybrid approach using Illumina short reads, Oxford Nanopore Technologies (ONT) long reads, and Hi-C data. We reveal the genomic features of *Aquilaria sinensis*, including repeat sequences, gene annotation, and evolution. This reference genome will provide the fundamental genetic information to elucidate the metabolic formation of agarwood and facilitate genetic research on *Aquilaria* trees.

## Data Description

### Genomic DNA extraction and genome size estimation

An individual plant of cultivar *Aquilaria sinensis* (Lour.) Spreng was collected from Chengxi district (110 19.245 E, 19 59.757 N), Haikou, China. After collection healthy, fresh leaves were snap-frozen in liquid nitrogen, followed by preservation at −80°C in the laboratory prior to DNA extraction. High molecular weight plant genomic DNA was extracted from these leaves using a modified CTAB method [15]. The quality and quantity of the isolated DNA were checked by electrophoresis on a 0.75% agarose gel and a NanoDrop D-1000 spectrophotometer (NanoDrop Technologies, Wilmington, DE), and the DNA was then accurately quantified using Qubit technology (Life Technologies, Carlsbad, CA). Subsequently, 150-bp paired-end (PE) libraries with insert lengths of 270 bp were constructed and 49.84 Gb raw data were generated on the Illumina Hiseq2500 platform (Illumina HiSeq 2500 System, RRID:SCR_016383) using standard protocols, which were used for estimating the genome size of *A. sinensis* with the following formula: genome size = [Num (total *k*-mer) − Num (erroneous *k*-mer)]/mean depth of *k*-mer[16, 17]. Finally, the genome size of A.*sinensis* was estimated as 773.3 Mb with the total number of 19-mer ∼3.71 × 10^10^ and the peak of 19-mer at the depth of 48 (Supplementary Fig. S1). The GC content of *A. sinensis* genome was 39.23%, which is considered a moderate GC level (Supplementary Fig. S2). Meanwhile, the heterozygosity of 0.6% and repeat content of 53.12% for the*A. sinensis* genome were also estimated [18].

### Genomic sequencing and assembly using Nanopore long reads

One Nanopore 1D library was prepared following the Oxford Nanopore SQK-LSK 108 kit and GridION protocol (Oxford Nanopore Technologies, Oxford, UK) [19]. Genomic DNA was first repaired and end-prepped with NEBNext FFPE Repair Mix (New England Biolabs [NEB]) and the NEBNext Ultra II End Repair/dA-Tailing Module (NEB). The DNA was then purified with AMPure XP beads (Beckmann Coulter) and ligated with sequencing adapters provided by ONT using concentrated T4 DNA ligase 2 M U mL^−1^ (NEB). After purification with AMPure XP beads (Beckman Coulter) using dilution buffer (ONT) and wash buffer (ONT), the library was mixed with sequencing buffer (ONT) and library loading beads (ONT) and loaded on 16 flow cells (R9.4) of GridION X5 platform (GridION, RRID:SCR_017986) [20], generating 71.3 Gb raw DNA reads (∼100× coverage of the genome assembly). We obtained 4.8 million Nanopore long reads (67.7 Gb in total) with an N50 read length of 21.29 kb and the longest read length of 935.06 kb after removing adapter sequences (Supplementary Table S1).

The clean long reads obtained from Nanopore were initially assembled by wtdbg (wtdbg, RRID:SCR_017225) version 1.3 [21] with parameters as follows: wtdbg -t 60 -i Passed.fastq -o Sample -H -k 17 -S 1.01 -e 4. The iterative polishing was conducted thrice by Pilon version 1.22 (Pilon, RRID:SCR_014731) [22] and BWA (BWA, RRID:SCR_010910) [23] with the default parameters. The Pilon program was also run with default parameters to fill gaps, fix bases (including single-nucleotide polymorphisms and indels), and correct local misassemblies. A total of 99.26% of Illumina short reads were able to align to the assembled genome (Supplementary Table S2). The primary draft genome assembly was 720 Mb with a contig N50 length of 1.1 Mb and the longest contig length of 11.9 Mb (Supplementary Table S3). The contig N50 of the *A. sinensis* genome was much higher than other published medicinal plants' genome assemblies (Supplementary Table S4).

### Hi-C library construction and chromosome-scale assembly

Hi-C, derived from chromosome conformation capture technology, is a method that probes the 3D architecture of whole genomes by coupling proximity-based ligation with massively parallel sequencing [24]. The Hi-C contract matrix has been widely used for assembly correction to generate chromosome-scale scaffolds. In this work, the genomic DNA used for the Hi-C library was extracted from a fresh leaf sample of *A. sinensis* using standard methods. The cross-linked DNA from lysed cells was digested with DpnII after cells were fixed with formaldehyde. Sticky ends were biotin labeled and proximity ligated to form chimeric junctions and then physically sheared to a size of 300–500 bp. Chimeric fragments representing the original cross-linked and long-distance physical interactions were then processed into PE sequencing libraries after PCR amplification. The PCR cycling protocol was as follows: with 95°C for 5 minutes; cycled 18×; 4°C for 30 seconds, 45°C for 1 second, 70°C for 20 seconds, and 98°C for 30 seconds; and then held at 4°C. The products of PCR were purified according to the Hi-C protocol and then the purified DNA was sheared, end-repaired, adenylation tailed, and universal adapter ligated, and samples were indexed as described in the manufacturer's recommendations [25].

**Figure 2:**
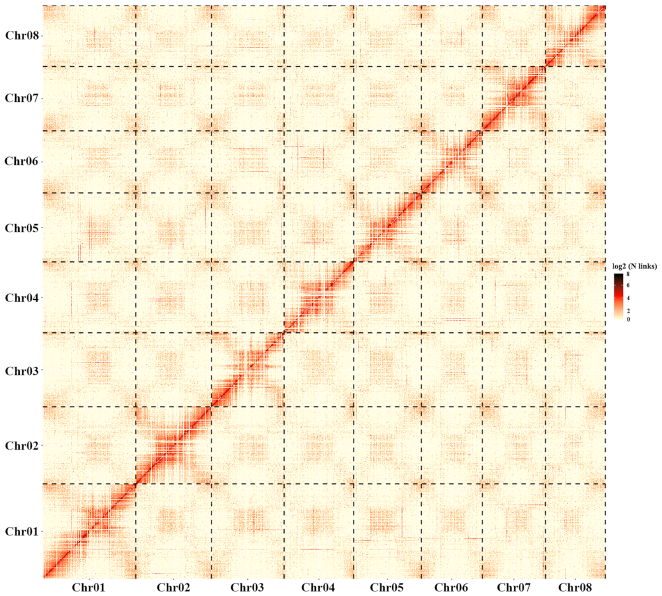
Hi-C interaction matrix for *A. sinensis* genome assembly using 8 clusters.

The whole-genome Hi-C library was sequenced with 150 bp PE sequencing on Illumina Hiseq 2500. A total of 714.27 million clean PE reads (∼103.07 Gb, roughly 142× coverage of assembled genome) were generated after filtering adapters and low-quality reads with Fastp (version 0.12.6) and HiC-Pro (HiC-Pro, RRID:SCR_017643) [26, 27]. By mapping the Hi-C data to the Nanopore-based assembly using bowtie2 (bowtie2, RRID:SCR_005476) [28], we found 93.49 million unique mapped PE reads and 62.89 million valid interaction pairs, which, respectively, accounted for 26.18% and 17.61% in the clean data (Supplementary Table S5). We used BWA and Lachesis (Lachesis, RRID:SCR_017644) software to align PE reads and retain the reads aligned to 500 bp away from each restriction site [29]. According to the methods of clustering, ordering, and orienting to the assembly contigs, these sequences were divided into 8 chromosome clusters and scaffolded by using Lachesis software with tuned parameters (Supplementary Table S6, Fig. 2). Finally, a heat map of Hi-C interaction for final assembly was produced using R (version 3.5.3) [30, 31].

A total of 1,862 contigs were used for scaffolding by Hi-C data, which consequently generated 805 scaffolds. The Hi-C–assisted chromosome-length scaffolds resulted in a final size of 716.6 Mb accounting for the 99.85% draft genome, which showed a high level of continuity with a contig N50 of 1.1 Mb and a scaffold N50 of 88.78 Mb. The final draft genome assembly of *A. sinensis* was 726.5 Mb (Supplementary Table S3). The anchor rate of contigs (*>*100 kb) to pseudochromosomes was attained up to the 98.63% level based on the Hi-C assembly (Table 1). The scaffold N50 of the *A. sinensis* genome was also superior to other published medicinal plant genome assemblies (Supplementary Table S4).

**Table 1:** Statistics of the final genome assembly for *Aquilaria sinensis*

Statistic	Contig length (bp)	Contig No.	Scaffold length (bp)	Scaffold No.
N50	1,058,652	164	88,784,932	4
N60	726,407	246	86,380,100	5
N70	495,861	366	84,956,755	6
Longest	11,913,571	1	109,870,270	1
Total	720,187,708	2,015	726,587,161	9
Length ≥1 kb	720,187,482	2,013	726,587,161	9
Length ≥2 kb	720,179,880	2,008	726,587,161	9
Length ≥5 kb	720,112,854	1,991	726,587,161	9

### RNA preparation and sequencing

Iso-seq was performed for genome assembly and annotation. The sample of mixed root, stem, and leaf used for RNA extraction was obtained from the same plant used for Oxford Nanopore DNA sequencing and immediately snap-frozen in liquid nitrogen. Total RNA was extracted from the frozen tissue using a Qiagen RNA extraction kit (Qiagen, Hilden, DE) and the sequencing library was then prepared with SMRTbell^TM^ template prep kit 1.0 (Pacific Biosciences, Menlo Park, CA, USA) after RNA reverse transcription with SMARTer^TM^ PCR cDNA Synthesis kit and complementary DNA (cDNA) amplification with KAPA HiFi PCR kits (Kapa Biosystems, Boston, Massachusetts, USA). Full-length transcriptome sequencing was subsequently performed using the PacBio Sequel System (PacBio Sequel System, RRID:SCR_017989). A total of 18,411,342 subreads were obtained from Iso-seq after raw data filtering with SMRTLING 5.1 and derived 136,050 consensus sequences, of which 94.71% (128,854) can be aligned to the final genome of *A. sinensis* (Supplementary Table S7).

### Genome quality evaluation

To evaluate the completeness of our assembly, we subjected the final assembled genome sequences to BUSCO version 3 (BUSCO, RRID:SCR_015008) (BUSCO, *Embryophyta* odb 10) [32, 33]. Overall, 95.27% of 1,375 expected embryophyta genes were identified in our genome assembly as the complete and partial BUSCO profiles. Among these identified 1,310 complete expected embryophyta genes, 1,202 and 108 were identified as single copy and duplicated copies, respectively (Supplementary Table S8).

### Repeat sequences within the *A. sinensis* genome assembly

Transposable elements (TEs) and tandem repeats were identified with both homology-based annotation and *de novo* methods. Consensus sequences of repetitive elements were *de novo* identified and classified using the software package RepeatModeler version 1.04 (RepeatModeler, RRID:SCR_015027) [34]. RepeatMask version 3.2.9 (RepeatMasker, RRID:SCR_012954) [34], RepeatProteinMasker [35], and TRF [36] were used to discover and identify repeats within the respective genomes. Furthermore, simple sequence repeats (SSRs) in the *A. sinensis* genome were also classified with MISA (MISA, RRID:SCR_010765) [37]. The results showed that *de novo* predicted repeats were more recently active than Repbase [38] predicted repeats (Supplementary Fig. S3). The identified repeat sequences in the *A. sinensis* genome assembly accounted for 59.13% and total length of those accounted for 425.87 Mb (Supplementary Table S9). In particular, the details showed that long terminal repeats (LTRs) were the most abundant repeat type and that 2 non-LTR retrotransposons, short interspersed nuclear elements (SINEs) and long interspersed nuclear elements (LINEs) [39], had the lowest proportions in the final assemblies. In addition, 13.12% of repeat sequences could not be classified (Table 2). A total of 367,251 SSRs were identified from the draft assembly in 675 scaffolds. Mononucleotides (64.71%), dinucleotides (18.19%), and trinucleotides (12.46%) comprised nearly 96% of SSRs in our assembly (Supplementary Table S10).

**Table 2:** Statistics of transposable elements in *Aquilaria sinensis* genome sequences

Type	Repbase TEs		Mips-REdat TEs		TE proteins		RepeatModeler		Combined TEs	
	Length (Mb)	% in genome	Length (Mb)	% in genome	Length (Mb)	% in genome	Length (Mb)	% in genome	Length (Mb)	% in genome
DNA	13,223,408	1.84	1,392,136	0.19	10,456,270	1.45	28,698 131	3.98	38,895,471	5.4
LINE	2,916,904	0.41	253,492	0.04	7,680,548	1.07	6,394,899	0.89	12,239,695	1.7
LTR	73,748,923	10.24	22,973,865	3.19	75,336,839	10.46	138,348,032	19.21	192,609,862	26.74
SINE	2,232	0	1,145	0	0	0	0	0	4,539	0
Other	6,189,190	0.86	380,555	0.05	1,369,337	0.19	0	0	87,659,087	12.17
Unknown	35,443	0	0	0	0	0	124,331,790	17.26	94,460,416	13.12
Total	96,116,100	13.35	25,001,193	3.47	94,842,994	13.17	296,679,047	41.19	425,869,070	59.13

### Gene prediction and annotation

Three strategies were used for gene prediction. Augustus version 3.2.3 (Augustus, RRID:SCR_008417) [40], GlimmHmm [41], and GeneID (GeneID, RRID:SCR_002473) [42] were used for *ab initio* gene prediction, using model training based on coding sequence (CDS) from *Corchorus olitorius* (COLO4_1.0) [43], *Durio zibethinus* (Duzib1.0) [44], *Gossypium hirsutum* (ASM98774v1) [45], *Herrania umbratica* (ASM216827v2) [46], *Theobroma cacao* (Cirollo_cocoa_geneoe_v2) [47], and *Arabidopsis thaliana* (TAIR10) [48]. GeneWise (GeneWise, RRID:SCR_015054) [49] and GeMoMa [50] were used for homology prediction. PASA (PASA, RRID:SCR_014656) [51] and Tophat (TopHat, RRID:SCR_013035) [52] were used for gene structural prediction based on expressed sequence tag and cDNA sequences. Finally, the total gene prediction was obtained from the union of these 3 strategies with EVM [51] and filtering the TEs with Transposon PSI (Transposon, RRID:SCR_001159) [53]. RNA-sequencing data of mixed tissues were mapped with the annotation of the reference genome using MatchAnnot [54], respectively.

The final annotation was composed of 29,203 gene models with an average of 3,177.62 bp transcripts and 1,114.16 bp CDS, each gene containing 5.02 exons with an average length of 222.09 bp. The comparative information of genes from *A. sinensis* and 6 closely related plants was also calculated (Supplementary Table S11), including their distributions of CDS and gene length, exon and intron length, and exon and intron number (Supplementary Fig. S4). Genes were characterized for their putative function by performing Blastall [55] and KAAS [56] searches of the peptide sequences against Swiss-Prot (Swiss-Prot, RRID:SCR_002380) [57], NR [58], TrEMBL (TrEMBL, RRID:SCR_002380) [57], KEGG database (KEGG, RRID:SCR_012773) [59], COG (Clusters of Orthologous Groups) database (COG, RRID:SCR_007273) [60], and the Gene Ontology (GO) database (GO, RRID:SCR_002811) [61]. Protein conservative models and motif prediction were performed with InterProScan version 5.2 (InterproScan, RRID:SCR_005829) [62]. Of these 29,203 protein-coding genes, 82.64% have functional annotation. The database research hits can be summarized as follows: Swiss-Prot (19,586 [67.07%]), NR (24,097 [82.52%]), TrEMBL (23,455 [80.32%]), KEGG (8,494 [29.09%]), COG (13,592 [46.54%]), GO (14,019 [48.00%]), and InterProScan (20,031 [68.59%]) (Supplementary Table S12). In addition, we also identified 207 microRNAs, 34 ribosomal RNAs, 173 transfer RNAs (tRNAs), and 1,173 small nuclear RNAs via the Rfam non-coding RNA database (Rfam, RRID:SCR_007891) [63], tRNAscan-SE (tRNAscan-SE, RRID:SCR_010835) [64], and RNAmmer [65]. The average length, total length, and percentage of non-coding RNAs in the *A. sinensis* genome were further assessed (Supplementary Table S13). In addition, 48.61% of predicted genes (14,197) were supported by Iso-seq transcripts (Supplementary Table S14).

### Gene family identification and phylogenetic tree construction

**Figure 3:**
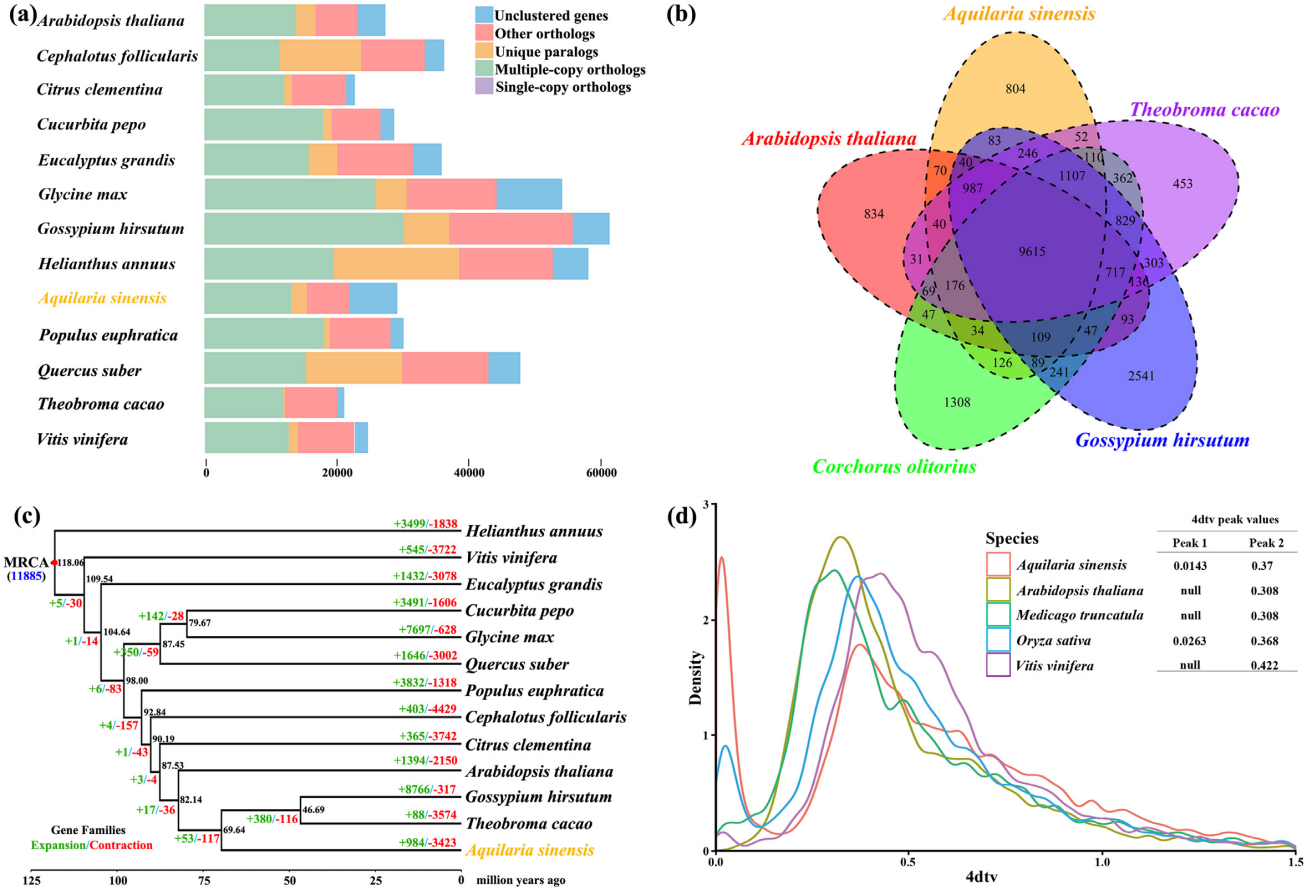
Comparative genomic analysis of *Aquilaria sinensis* and other plant species. (a) Distribution of genes and gene families of 13 plant species we investigated. (b) Venn diagram showing the distribution of shared gene families among the Malvales plants *Aquilaria sinensis* (agarwood), *Theobroma cacao* (cocoa), *Gossypium hirsutum* (cotton), *Corchorus olitorius* (jute), and the model plant *Arabidopsis thaliana* (Arabidopsis). (c) Divergence time estimation and gene family changes among 13 plant species. The black number at each node denotes estimated divergence time from present (million years ago). The blue number at the root (11,885) denotes the total number of gene families predicted in the most recent common ancestor (MRCA), and the green/red numbers around each branch denote gene family gain/loss number. The red nodes indicate the known divergence time of Asterids and Rosids. (d) Transversion substitutions at 4-fold degenerate sites (4dTv) distribution in selected assemblies of *A. sinensis, A. thaliana, O. sativa, M. truncatula*, and *V. vinifera*.

By keeping the longest transcript for each gene, whole protein coding gene sets from the*A. sinensis* genome and 12 other representative plant genomes including *G. hirsutum* (ASM98774v1), *A. thaliana* (TAIR10), *T. cacao* (Cirollo_cocoa_geneoe_v2), *Cephalotus follicularis* (Cfol_1.0), *Citrus clementina* (Citrus_clementina_v1.0), *Cucurbita pepo* (ASM280686v2), *Eucalyptus grandis* (Egrandis1_0), *Glycine max* (Glycine_max_v2.1), *Helianthus annuus* (HanXRQr 1.0), *Populus euphratica* (PopEup_1.0), *Quercus suber* (CorkOak 1.0), and *Vitis vinifera* (assembly 12X) were used to construct a global gene family classification with all-vs-all BLASTP (1e^−5^ cutoff, Blast+ v2.3.056) and OrthoMCL version 2.0.9 (Ortholog Groups of Protein Sequences, RRID:SCR_007839) [66]. The default settings were used for BLASTP and OrthoMCL. In our assembly, 21,955 genes were clustered into 13,713 gene families. Gene family analysis also revealed that 789 gene families and 7,248 genes were unique to *A. sinensis* in the above comparison (Fig. 3a and Supplementary Table S15). Of these, 9,615 gene families were shared among *A. sinensis* and 4 representative species (*G. hirsutum* from Malvaceae, *C. olitorius* from Tiliaceae, *T. cacao* from Sterculiaceae, and *A. thaliana* as the model plant from Cruciferae), whereas 804 gene families were unique to the *A. sinensis* genome (Fig. 3b). Malvaceae, Tiliaceae, and Sterculiaceae are beyond the order Malvales, and the Thymelaeceae family is also divided into order Malvales in APG IV [67].

Single-copy genes, or the orphan genes with only a single copy in the genome during duplication and evolution of species, are highly conserved and are generally used for establishing genetic relationship and origin of species. Alignment of single-copy genes was performed with protein sequences by Mafft (Mafft, RRID:SCR_011811) [68], then poorly aligned and highly divergent sites were subjected to filtering with Gblocks (Gblocks, RRID:SCR_015945) [69] and the final CDSs were used for evolutional analyses by RAxML with GTRGAMMA model (RAxML, RRID:SCR_006086) [70]. The bootstrap was 100 and *H. annuus* from the Asterids was the outgroup [71]. We constructed a phylogenetic tree and estimated the divergence time of 13 plants by 89 single-copy gene families with the MCMCTREE of PAML [72] (Supplementary Fig. S5) (Parameters: clock = 2, RootAge = < 100.6, model = 7, BDparas = 1 1 0, kappa gamma = 6 2, alpha gamma = 1 1, rgene gamma = 2 3.18, sigma2 gamma = 1 1.3; divergence time of Asterids and Rosids [∼118 million years ago (Mya)] was used for calibration [71]). The divergence time between *A. sinensis* and *A. thaliana* was estimated as 82.14 (95% CI, 67.63–93.99) Mya, and the divergence time between *A. sinensis* and the common ancestor of *G. hirsutum* and *T. cacao* from Malvales order was ∼69.64 (95% CI, 53.18–84.37) Mya (Fig. 3c and Supplementary Fig. S6), whereas the divergence time between *G. hirsutum* and *T. cacao* was determined as 31.33–69.23 Mya in our analysis, which is concordance with the previous studies [44, 45].

### Gene family expansion and contraction

Expansion and contraction of a defining gene family is an important driver of metabolite variation and species adaptation during plant evolution [73]. We determined the expansion and contraction of orthologous gene families in the *A. sinensis* genome by means of CAFÉ 2.2 (CAFÉ, RRID:SCR_005983) with default parameters [74]. We inferred 53 expanded families and 117 contracted families with the *A. sinensis* genome after comparing 11,855 gene families across all 13 species (Fig. 3c and Supplementary Table S16). We used Blast2GO (B2G, RRID:SCR_005828) to enrich the ontology categories (GO and KEGG terms). The expanded gene families were involved in the pathways of plant circadian rhythm, tricarboxylic acid cycle, propanoate metabolism, ribosome biogenesis, and aminoacyl-tRNA biosynthesis (Supplementary Table S17 and Fig. S7), and the contracted gene families mapped pathways of starch/sucrose metabolism, sesquiterpenoid and triterpenoid biosynthesis, and linoleic acid metabolism (Supplementary Table S18 and Fig. S8).

### 4DTv Distribution

We used MCScanX to identify the syntenic regions [75], with the longest isoform for each gene selected for this exercise. The top 5 mutual hits of the BLASTP results in gene family analysis were used as input. Only the syntenic segments that have >5 gene pairs were considered for 4-fold degenerate synonymous site (4DTv) calculation. Pairwise sequence was aligned using MUSCLE [76]. Raw 4DTv values were corrected for possible multiple transversions at the same site. Based on 4DTv distribution, a large accumulation of gene duplications is evident in the *A. sinensis* genome and distinct from the scenarios in *A. thaliana, M. truncatula*, and *V. vinifera* (Fig. 3d).

## Conclusions

In summary, a high-quality *de novo* genome assembly and in-depth characterization of *A. sinensis*, combining Nanopore single-molecule long reads and Hi-C, has been provided in this study. The final assembly was ∼726.5 Mb in size, which was slightly smaller than the *k*-mer estimated genome size of 773.3 Mb. The Hi-C data were used to identify and revise 230 misassemblies and assign the contigs into chromosome-scale scaffolds. This consequently generated an assembly with a high level of continuity with a contig N50 of 1.1 Mb and a scaffold N50 of 88.78 Mb. We also predicted 29,203 protein-coding genes from the final assembly and 82.64% (24,133 genes) of all protein-coding genes were annotated. We estimated that the divergence time between *A. sinensis* and its common ancestor *G. hirsutum* and *T. cacao* from the Malvales order was ∼53.18–84.37 Mya. The genome of *A. sinensis* seems to have experienced a recent whole-genome duplication event after the K-T boundary [77]. The chromosome-level genome assembly of *A. sinensis* is also the first high-quality genome in the Thymelaeceae family. Considering that wild *A. sinensis* tree populations are currently highly threatened by heavy exploitation for the production of commercially valuable agarwood products, the genome assembly of the *A. sinensis* tree presented here will provide valuable information to aid the global conservation of these precious biological resources and contribute to understanding the mechanism of agarwood formation, which eventually will help us reveal the evolution of aromatic genes and plants.

## Availability of Supporting Data and Materials

Supporting data and materials are available in the *GigaScience* GigaDB database [78], with the raw genomics and transcriptome sequences deposited in the NCBI SRA database under the BioProject accession number PRJNA556948 and BioSample accession number SAMN12385133.

## Additional files

**Supplementary Figure 1:**  *k*-mer (*k* = 19) analysis for estimating the size of the *Aquilaria sinensis* genome.

**Supplementary Figure 2:** GC content and average sequencing depth of the Illumina sequencing data used for genome estimation.

**Supplementary Figure 3:** Distribution of sequence divergence rates of different TE types with Repbase (A) and *de novo* (B) methods in the *Aquilaria sinensis* genome.

**Supplementary Figure 4:** Distribution of gene elements in *Aquilaria sinensis* genome and 6 other plant genomes.

**Supplementary Figure 5:** Phylogenetic tree of 13 plant species including *Aquilaria sinensis*.

**Supplementary Figure 6:** Estimation of divergence time of 13 plant species investigated in the present study. The colored numbers on the nodes are the divergence time from present (million years ago). Numbers in parentheses indicate the 95% confidence interval of the divergence time.

**Supplementary Figure 7:** GO enrichment of expansion gene families in *Aquilaria sinensis* genome.

**Supplementary Figure 8:** GO enrichment of contraction gene families in *Aquilaria sinensis* genome.

**Supplementary Table 1:** Summary of Nanopore sequencing for *Aquilaria sinensis* genome.

**Supplementary Table 2:** Support of Illumina data for Nanopore data in *Aquilaria sinensis* genome assembly.

**Supplementary Table 3:** Statistics of the results of *Aquilaria sinensis* genome assembly before Hi-C mapping.

**Supplementary Table 4:** Comparisons of genome assemblies of medicinal plants based on descending contig N50.

**Supplementary Table 5:** Summary of mapping status of Hi-C data.

**Supplementary Table 6:** Statistics of initial and final assembly with Hi-C.

**Supplementary Table 7:** Mapping result of Iso-seq from *Aquilaria sinensis*.

**Supplementary Table 8:** Statistics of BUSCO evolution for *Aquilaria sinensis* genome.

**Supplementary Table 9:** Statistics of repeat sequence in *Aquilaria sinensis* genome via different methods.

**Supplementary Table 10:** Statistics of SSRs in *Aquilaria sinensis* genome sequences.

**Supplementary Table 11:** Statistics of characteristics of gene models in *Aquilaria sinensis* and 6 other plant genomes.

**Supplementary Table 12:** The annotated genes of *Aquilaria sinensis* that can be functionally classified in each corresponding database.

**Supplementary Table 13:** Noncoding RNA annotation in the *Aquilaria sinensis* genome.

**Supplementary Table 14:** Annotation of Iso-seq and comparison with genome annotation of the *Aquilaria sinensis* genome.

**Supplementary Table 15:** Summary of gene families among 13 plant species.

**Supplementary Table 16:** Summary of gene family changes among 13 species.

**Supplementary Table 17:** KEGG mapping of expansion gene families in *Aquilaria sinensis* genome.

**Supplementary Table 18:** KEGG mapping of contraction gene families in *Aquilaria sinensis* genome.

giaa013_GIGA-D-19-00288_Original_Submission

giaa013_GIGA-D-19-00288_Revision_1

giaa013_GIGA-D-19-00288_Revision_2

giaa013_Response_to_Reviewer_Comments_Original_Submission

giaa013_Response_to_Reviewer_Comments_Revision_1

giaa013_Reviewer_1_Report_Original_Submission Caroline Belser -- 9/5/2019 Reviewed

giaa013_Reviewer_1_Report_Revision_1 Caroline Belser -- 12/16/2019 Reviewed

giaa013_Reviewer_2_Report_Original_Submission Fernando Cruz -- 9/29/2019 Reviewed

giaa013_Reviewer_2_Report_Revision_1 Fernando Cruz -- 1/9/2020 Reviewed

giaa013_Supplemental_Files

## Abbreviations

4DTv: 4-fold degenerate synonymous sites; APG: Angiosperm Phylogeny Group; bp: base pairs; BUSCO: Benchmarking Universal Single-Copy Orthologs; CAFÉ: Computational Analysis of Gene Family Evolution; cDNA: complementary DNA; CDS: coding sequence; COG: Clusters of Orthologous Groups; CTAB: cetyl trimethylammonium bromide; EVM: EVidenceModeler; FFPE: formalin-fixed paraffin-embedded; Gb: gigabase pairs; GC: guanine-cytosine; GO: Gene Ontology; Hi-C: high-throughput chromosome conformation capture; Iso-seq: Isoform sequencing; IUCN: International Union for Conservation of Nature and Natural Resources; KAAS: KEGG Automatic Annotation Server; kb: kilobase pairs; KEGG: Kyoto Encyclopedia of Genes and Genomes; K-T: Cretaceous-Tertiary; LINE: long interspersed nuclear element; Mb: megabase pairs; MRCA: most recent common ancestor; Mya: million years ago; NCBI: National Center for Biotechnology Information; NEB: New England Biolabs; Nr: non-redundant protein database; ONT: Oxford Nanopore Technologies; PASA: Program to Assemble Spliced Alignments; PE: paired end; Pfam: protein families; RAxML: Randomized Axelerated Maximum Likelihood; SINE: short interspersed nuclear element; SMRT: single-molecule real time; SRA: Sequence Read Archive; SSR: simple sequence repeat; TE: transposable element; TrEMBL: Translated EMBL-Bank; TRF: Tandem Repeats Finder; tRNA: transnfer RNA; CI: confidence interval.

## Competing Interests

The authors declare that they have no competing interests.

## Funding

This work was supported by the Central Public-interest Scientific Institution Basal Research Fund for Chinese Academy of Tropical Agricultural Sciences (17CXTD-15 and 1630052020003), the National Natural Science Foundation of China (31870668) and the China Agriculture Research System (CARS-21). We are grateful to NextOmics Co., Ltd. (Wuhan, China), for providing technical help.

## Authors' Contributions

H.F.D., P.C., and W.L.M. conceptualized the research program. X.P.D., W.L.M., and S.Q.P. designed experiments and coordinated the program. S.Z.H. collected the specimens and J.W. took the photos. H.L.L and J.H.Z. extracted the DNA. X.P.D., Q.L., P.W., P.C., W.L., H.Q.C., W.H.D., D.G., and C.H.C were partially involved with either experiments or data analysis. X.P.D. and Q.L. wrote the manuscript. All authors read and approved the final manuscript.

## References

[1] Kumeta Y, Ito M Characterization of α-humulene synthases responsible for the production of sesquiterpenes induced by methyl jasmonate in *Aquilaria* cell culture. J Nat Med. 2016;70(3):452–9. 27180085 10.1007/s11418-016-0999-8

[2] Xu Y, Zhang Z, Wang M, et al. Identification of genes related to agarwood formation: transcriptome analysis of healthy and wounded tissues of *Aquilaria sinensis*. BMC Genomics. 2013;14(1):227. 23565705 10.1186/1471-2164-14-227 PMC3635961

[3] Naef R The volatile and semi‐volatile constituents of agarwood, the infected heartwood of *Aquilaria* species: a review. Flavour Fragr J. 2011;26(2):73–87.

[4] Liao G, Mei WL, Kong FD, et al. 5, 6, 7, 8-Tetrahydro-2-(2-phenylethyl) chromones from artificial agarwood of *Aquilaria sinensis* and their inhibitory activity against acetylcholinesterase. Phytochemistry. 2017;139:98–108. 28433955 10.1016/j.phytochem.2017.04.011

[5] Hashim YZHY, Kerr PG, Abbas P, et al. *Aquilaria* spp.(agarwood) as source of health beneficial compounds: a review of traditional use, phytochemistry and pharmacology. J Ethnopharmacol. 2016;189:331–60. 27343768 10.1016/j.jep.2016.06.055

[6] Ma CT, Eom T, Cho E, et al. Aquilanols A and B, macrocyclic humulene-type sesquiterpenoids from the agarwood of *Aquilaria malaccensis*. J Nat Prod. 2017;80(11):3043–8. 29083898 10.1021/acs.jnatprod.7b00462

[7] Yang L, Yang YL, Dong WH, et al. Sesquiterpenoids and 2-(2-phenylethyl) chromones respectively acting as α-glucosidase and tyrosinase inhibitors from agarwood of an *Aquilaria* plant. J Enzyme Inhib Med Chem. 2019;34(1):853–62. 31010356 10.1080/14756366.2019.1576657 PMC6495113

[8] Liao G, Dong WH, Yang JL, et al. Monitoring the chemical profile in agarwood formation within one year and speculating on the biosynthesis of 2-(2-phenylethyl) chromones. Molecules. 2018;23(6):1261. 10.3390/molecules23061261 PMC6100365 29799457

[9] Chhipa H, Chowdhary K, Kaushik N Artificial production of agarwood oil in *Aquilaria* sp. by fungi: a review. Phytochem Rev. 2017;16(5):835–60.

[10] Azren PD, Lee SY, Emang D, et al. History and perspectives of induction technology for agarwood production from cultivated *Aquilaria* in Asia: a review. J For Res. 2019;30(1):1–11.

[11] Harvey-Brown Y *Aquilaria sinensis* The IUCN Red List of Threatened Species2018.doi:10.2305/IUCN.UK.2018-2.RLTS.T32382A2817115.en.

[12] Wang Y, Zhan DF, Jia X, et al. Complete chloroplast genome sequence of *Aquilaria sinensis* (Lour.) Gilg and evolution analysis within the Malvales order. Front Plant Sci. 2016;7:280. 27014304 10.3389/fpls.2016.00280 PMC4781844

[13] Wang X, Gao B, Liu X, et al. Salinity stress induces the production of 2-(2-phenylethyl) chromones and regulates novel classes of responsive genes involved in signal transduction in *Aquilaria sinensis* calli. BMC Plant Biol. 2016;16(1):119. 27230436 10.1186/s12870-016-0803-7 PMC4881210

[14] Wang X, Zhang Z, Dong X, et al. Identification and functional characterization of three type III polyketide synthases from *Aquilaria sinensis* calli. Biochem Biophys Res Commun. 2017;486(4):1040–7. 28366630 10.1016/j.bbrc.2017.03.159

[15] Porebski S, Bailey LG, Baum BR Modification of a CTAB DNA extraction protocol for plants containing high polysaccharide and polyphenol components. Plant Mol Biol Rep. 1997;15(1):8–15.

[16] Liu B, Shi Y, Yuan J, et al. Estimation of genomic characteristics by analyzing k-mer frequency in de novo genome projects. arXiv. 2013:1308.2012.

[17] Ding X, Mei W, Huang S, et al. Genome survey sequencing for the characterization of genetic background of *Dracaena cambodiana* and its defense response during dragon's blood formation. PLoS One. 2018;13(12):e0209258. 30550595 10.1371/journal.pone.0209258 PMC6294377

[18] Vurture GW, Sedlazeck FJ, Nattestad M, et al. GenomeScope: fast reference-free genome profiling from short reads. Bioinformatics. 2017;33(14):2202–4. 28369201 10.1093/bioinformatics/btx153 PMC5870704

[19] Leggett RM, Clark MD A world of opportunities with nanopore sequencing. J Exp Bot. 2017;68(20):5419–29. 28992056 10.1093/jxb/erx289

[20] Schmidt MHW, Vogel A, Denton AK, et al. De novo assembly of a new *Solanum pennellii* accession using nanopore sequencing. Plant Cell. 2017;29(10):2336–48. 29025960 10.1105/tpc.17.00521 PMC5774570

[21] Ruan J, Li H Fast and accurate long-read assembly with wtdbg2. Nat Methods. 2020;17:155–8. 31819265 10.1038/s41592-019-0669-3 PMC7004874

[22] Walker BJ, Abeel T, Shea T, et al. Pilon: an integrated tool for comprehensive microbial variant detection and genome assembly improvement. PLoS One. 2014;9(11):e112963. 25409509 10.1371/journal.pone.0112963 PMC4237348

[23] Li H, Durbin R Fast and accurate long-read alignment with Burrows-Wheeler transform. Bioinformatics. 2010;26(5):589–95. 20080505 10.1093/bioinformatics/btp698 PMC2828108

[24] Lieberman-Aiden E, Van Berkum NL, Williams L, et al. Comprehensive mapping of long-range interactions reveals folding principles of the human genome. Science. 2009;326(5950):289–93. 19815776 10.1126/science.1181369 PMC2858594

[25] Xu CQ, Liu H, Zhou SS, et al. Genome sequence of *Malania oleifera*, a tree with great value for nervonic acid production. Gigascience. 2019;8(2):giy164. 30689848 10.1093/gigascience/giy164 PMC6377399

[26] Chen S, Zhou Y, Chen Y, et al. fastp: an ultra-fast all-in-one FASTQ preprocessor. Bioinformatics. 2018;34(17):i884–90. 30423086 10.1093/bioinformatics/bty560 PMC6129281

[27] Servant N, Varoquaux N, Lajoie BR, et al. HiC-Pro: an optimized and flexible pipeline for Hi-C data processing. Genome Biol. 2015;16(1):259. 26619908 10.1186/s13059-015-0831-x PMC4665391

[28] Langmead B, Salzberg SL Fast gapped-read alignment with Bowtie 2. Nat Methods. 2012;9(4):357. 22388286 10.1038/nmeth.1923 PMC3322381

[29] Burton JN, Adey A, Patwardhan RP, et al. Chromosome-scale scaffolding of de novo genome assemblies based on chromatin interactions. Nat Biotechnol. 2013;31(12):1119. 24185095 10.1038/nbt.2727 PMC4117202

[30] R Core Team. R: A language and environment for statistical computing. 2019 https://www.R-project.org/.Accessed 24 November 2018.

[31] Yin D, Ji C, Ma X, et al. Genome of an allotetraploid wild peanut *Arachis monticola*: a de novo assembly. Gigascience. 2018;7(6):giy066. 10.1093/gigascience/giy066 PMC6009596 29931126

[32] Simão FA, Waterhouse RM, Ioannidis P, et al. BUSCO: assessing genome assembly and annotation completeness with single-copy orthologs. Bioinformatics. 2015;31(19):3210–2. 26059717 10.1093/bioinformatics/btv351

[33] Waterhouse RM, Seppey M, Simão FA, et al. BUSCO applications from quality assessments to gene prediction and phylogenomics. Mol Biol Evol. 2017;35(3):543–8. 10.1093/molbev/msx319 PMC5850278 29220515

[34] Bedell JA, Korf I, Gish W MaskerAid: a performance enhancement to RepeatMasker. Bioinformatics. 2000;16(11):1040–1. 11159316 10.1093/bioinformatics/16.11.1040

[35] Allred DB, Cheng A, Sarikaya M, et al. Three-dimensional architecture of inorganic nanoarrays electrodeposited through a surface-layer protein mask. Nano Lett. 2008;8(5):1434–8. 18376869 10.1021/nl0803444

[36] Benson G Tandem Repeats Finder: a program to analyze DNA sequences. Nucleic Acids Res. 1999;27(2):573–80. 9862982 10.1093/nar/27.2.573 PMC148217

[37] Thiel T, Michalek W, Varshney R, et al. Exploiting EST databases for the development and characterization of gene-derived SSR-markers in barley (*Hordeum vulgare* L.). Theor Appl Genet. 2003;106(3):411–22. 12589540 10.1007/s00122-002-1031-0

[38] Jurka J, Kapitonov VV, Pavlicek A, et al. Repbase Update, a database of eukaryotic repetitive elements. Cytogenet Genome Res. 2005;110(1-4):462–7. 16093699 10.1159/000084979

[39] Yang L, Scott LA, Wichman HA Tracing the history of LINE and SINE extinction in sigmodontine rodents. Mobile DNA. 2019;10(1):22. 31139266 10.1186/s13100-019-0164-5 PMC6530004

[40] Stanke M, Steinkamp R, Waack S, et al. AUGUSTUS: a web server for gene finding in eukaryotes. Nucleic Acids Res. 2004;32(suppl 2):W309–12. 15215400 10.1093/nar/gkh379 PMC441517

[41] Majoros WH, Pertea M, Salzberg SL TigrScan and GlimmerHMM: two open source ab initio eukaryotic gene-finders. Bioinformatics. 2004;20(16):2878–9. 15145805 10.1093/bioinformatics/bth315

[42] Blanco E, Parra G, Guigó R Using geneid to identify genes. Curr Protoc Bioinformatics. 2007;18(1):4.3. 1–4.3. 28. 10.1002/0471250953.bi0403s18 18428791

[43] Islam MS, Saito JA, Emdad EM, et al. Comparative genomics of two jute species and insight into fibre biogenesis. Nat Plants. 2017;3(2):16223. 28134914 10.1038/nplants.2016.223

[44] Teh BT, Lim K, Yong CH, et al. The draft genome of tropical fruit durian (*Durio zibethinus*). Nat Genet. 2017;49(11):1633. 28991254 10.1038/ng.3972

[45] Li F, Fan G, Lu C, et al. Genome sequence of cultivated upland cotton (*Gossypium hirsutum* TM-1) provides insights into genome evolution. Nat Biotechnol. 2015;33(5):524. 25893780 10.1038/nbt.3208

[46] *Herrania umbratica*genome. https://www.ncbi.nlm.nih.gov/genome/55117.

[47] Argout X, Martin G, Droc G, et al. The cacao Criollo genome v2.0: an improved version of the genome for genetic and functional genomic studies. BMC Genomics. 2017;18(1):730. 28915793 10.1186/s12864-017-4120-9 PMC5603072

[48] Michael TP, Jupe F, Bemm F, et al. High contiguity *Arabidopsis thaliana* genome assembly with a single nanopore flow cell. Nat Commun. 2018;9(1):541. 29416032 10.1038/s41467-018-03016-2 PMC5803254

[49] Birney E, Durbin R Using GeneWise in the*Drosophila* annotation experiment. Genome Res. 2000;10(4):547–8. 10779496 10.1101/gr.10.4.547 PMC310858

[50] Keilwagen J, Hartung F, Grau J GeMoMa: Homology-Based Gene Prediction Utilizing Intron Position Conservation and RNA-seq Data. Gene Prediction. New York, NY: Human; 2019:161–77. 10.1007/978-1-4939-9173-0_9 31020559

[51] Haas BJ, Salzberg SL, Zhu W, et al. Automated eukaryotic gene structure annotation using EVidenceModeler and the Program to Assemble Spliced Alignments. Genome Biol. 2008;9(1):R7. 18190707 10.1186/gb-2008-9-1-r7 PMC2395244

[52] Trapnell C, Pachter L, Salzberg SL TopHat: discovering splice junctions with RNA-Seq. Bioinformatics. 2009;25(9):1105–11. 19289445 10.1093/bioinformatics/btp120 PMC2672628

[53] Yagi M, Kosugi S, Hirakawa H, et al. Sequence analysis of the genome of carnation (*Dianthus caryophyllus* L.). DNA Res. 2013;21(3):231–41. 24344172 10.1093/dnares/dst053 PMC4060945

[54] Hu J, Uapinyoying P, Goecks J Interactive analysis of long-read RNA isoforms with Iso-Seq Browser. bioRxiv. 2017, doi:10.1101/102905.

[55] NCBI Resource Coordinators. Database resources of the National Center for Biotechnology Information. Nucleic Acids Res. 2017;45:D12. 27899561 10.1093/nar/gkw1071 PMC5210554

[56] Moriya Y, Itoh M, Okuda S, et al. KAAS: an automatic genome annotation and pathway reconstruction server. Nucleic Acids Res. 2007;35(suppl 2):W182–5. 17526522 10.1093/nar/gkm321 PMC1933193

[57] Boeckmann B, Bairoch A, Apweiler R, et al. The SWISS-PROT protein knowledgebase and its supplement TrEMBL in 2003. Nucleic Acids Res. 2003;31(1):365–70. 12520024 10.1093/nar/gkg095 PMC165542

[58] Yu K, Zhang T Construction of customized sub-databases from NCBI-nr database for rapid annotation of huge metagenomic datasets using a combined BLAST and MEGAN approach. PLoS One. 2013;8(4):e59831. 23573212 10.1371/journal.pone.0059831 PMC3613424

[59] Kanehisa M, Furumichi M, Tanabe M, et al. KEGG: new perspectives on genomes, pathways, diseases and drugs. Nucleic Acids Res. 2016;45(D1):D353–61. 27899662 10.1093/nar/gkw1092 PMC5210567

[60] Kristensen DM, Kannan L, Coleman MK, et al. A low-polynomial algorithm for assembling clusters of orthologous groups from intergenomic symmetric best matches. Bioinformatics. 2010;26(12):1481–7. 20439257 10.1093/bioinformatics/btq229 PMC2881409

[61] Gene Ontology Consortium. Gene Ontology Consortium: going forward. Nucleic Acids Res. 2014;43(D1):D1049–56. 25428369 10.1093/nar/gku1179 PMC4383973

[62] Hunter S, Apweiler R, Attwood TK, et al. InterPro: the integrative protein signature database. Nucleic Acids Res. 2008;37(suppl 1):D211–5. 18940856 10.1093/nar/gkn785 PMC2686546

[63] Griffiths-Jones S, Moxon S, Marshall M, et al. Rfam: annotating non-coding RNAs in complete genomes. Nucleic Acids Res. 2005;33(suppl 1):D121–4. 15608160 10.1093/nar/gki081 PMC540035

[64] Lowe TM, Eddy SR tRNAscan-SE: a program for improved detection of transfer RNA genes in genomic sequence. Nucleic Acids Res. 1997;25(5):955–64. 9023104 10.1093/nar/25.5.955 PMC146525

[65] Lagesen K, Hallin P, Rødland EA, et al. RNAmmer: consistent and rapid annotation of ribosomal RNA genes. Nucleic Acids Res. 2007;35(9):3100–8. 17452365 10.1093/nar/gkm160 PMC1888812

[66] Li L, Stoeckert CJ, Roos DS OrthoMCL: identification of ortholog groups for eukaryotic genomes. Genome Res. 2003;13(9):2178–89. 12952885 10.1101/gr.1224503 PMC403725

[67] Chase MW, Christenhusz MJM, Fay MF, et al. An update of the Angiosperm Phylogeny Group classification for the orders and families of flowering plants: APG IV. Bot J Linn Soc. 2016;181(1):1–20.

[68] Katoh K, Standley DM MAFFT multiple sequence alignment software version 7: improvements in performance and usability. Mol Biol Evol. 2013;30(4):772–80. 23329690 10.1093/molbev/mst010 PMC3603318

[69] Castresana J Selection of conserved blocks from multiple alignments for their use in phylogenetic analysis. Mol Biol Evol. 2000;17(4):540–52. 10742046 10.1093/oxfordjournals.molbev.a026334

[70] Stamatakis A RAxML-VI-HPC: maximum likelihood-based phylogenetic analyses with thousands of taxa and mixed models. Bioinformatics. 2006;22(21):2688–90. 16928733 10.1093/bioinformatics/btl446

[71] Badouin H, Gouzy J, Grassa CJ, et al. The sunflower genome provides insights into oil metabolism, flowering and Asterid evolution. Nature. 2017;546(7656):148. 28538728 10.1038/nature22380

[72] Yang Z PAML 4: phylogenetic analysis by maximum likelihood. Mol Biol Evol. 2007;24(8):1586–91. 17483113 10.1093/molbev/msm088

[73] Denoeud F, Carretero-Paulet L, Dereeper A, et al. The coffee genome provides insight into the convergent evolution of caffeine biosynthesis. Science. 2014;345(6201):1181–4. 25190796 10.1126/science.1255274

[74] De Bie T, Cristianini N, Demuth JP, et al. CAFE: a computational tool for the study of gene family evolution. Bioinformatics. 2006;22(10):1269–71. 16543274 10.1093/bioinformatics/btl097

[75] Wang Y, Tang H, DeBarry JD, et al. MCScanX: a toolkit for detection and evolutionary analysis of gene synteny and collinearity. Nucleic Acids Res. 2012;40(7):e49. 22217600 10.1093/nar/gkr1293 PMC3326336

[76] Edgar RC MUSCLE: multiple sequence alignment with high accuracy and high throughput. Nucleic Acids Res. 2004;32(5):1792–7. 15034147 10.1093/nar/gkh340 PMC390337

[77] Fawcett JA, Maere S, Van De Peer Y Plants with double genomes might have had a better chance to survive the Cretaceous–Tertiary extinction event. Proc Natl Acad Sci U S A. 2009;106(14):5737–42. 19325131 10.1073/pnas.0900906106 PMC2667025

[78] Ding X, Mei W, Lin Q, et al. Supporting data for “Genome sequence of agarwood tree *Aquilaria sinensis* (Lour.) Spreng: the first chromosome-level draft genome in the Thymelaeceae family.". GigaScience Database. 2020 10.5524/100702. PMC7050300 32118265

