# Supplementary material for: Genome sequence of the agarwood tree Aquilaria sinensis (Lour.) Spreng: the first chromosome-level draft genome in the Thymelaeceae family
Source: Gigascience. 2020 Mar 2;9(3):giaa013. doi: 10.1093/gigascience/giaa013 (PMC7050300; doi:10.1093/gigascience/giaa013)
Supplement: giaa013_GIGA-D-19-00288_Original_Submission [file giaa013_giga-d-19-00288_original_submission.pdf]

GigaScience

Genome sequence of agarwood tree Aquilaria sinensis (Lour.) Spreng: the first chromosome-level draft genome in the Thymelaeceae family

--Manuscript Draft--

**Manuscript Number:**	GIGA-D-19-00288
**Full Title:**	Genome sequence of agarwood tree Aquilaria sinensis (Lour.) Spreng: the first chromosome-level draft genome in the Thymelaeceae family
**Article Type:**	Data Note
**Funding Information:**	Central Public-interest Scientific Institution Basal Research Fund for Chinese Academy of Tropical Agricultural Sciences (17CXTD-15) — Prof. Wenli Mei<br>National Natural Science Foundation of China (31870668) — Prof. Wenli Mei<br>China Agriculture Research System (CARS-21) — Prof. Haofu Dai
**Abstract:**	Backgroud: Aquilaria sinensis (Lour.) Spreng is one of important plant resources for producing agarwood in China. The agarwood collected from the wounded Aquilaria trees have been used for aromatic or medicinal purposes in these regions from the ancient time, whereas the mechanism underlying the formation of agarwood still remained poorly understood by a lack of accurate and high-quality genetics information. Findings: We report genomic architecture of A.sinensis by an integrated strategy combining with Nanopore sequencing, Illumina sequencing and Hi-C sequencing. The final genome was approximately 726.5 Mb, which reached a high level of continuity with a contig N50 of 1.1 Mb. We combined Hi-C data with the genome assembly to generate chromosome-level scaffolds. Eight super-scaffolds corresponding to the 8 chromosomes were assembled to a final size of 716.6 Mb, with a scaffold N50 of 88.78 Mb using 1,862 contigs. Benchmarking Universal Single-Copy Orthologs evaluation reveals that the genome completeness reaches 95.27%. The repeat sequences are accounted for 59.13% and the protein-coding genes are annotated for 29,203 in the entire genome. According to phylogenetic analysis using single-copy orthologous genes, we found that A.sinensis is closely related to Gossypium hisutum and Theobroma cacao from the Malvales order, and A.sinensis was diverged from their common ancestor approximately 53.18-84.37 million years ago. We also found a recent whole-genome duplication (WGD) event in A.sinensis genome. Conclusions: Here, we represent the first chromosome-level genome assembly and gene annotation of A.sinensis. This study would contribute to provide valuable genetic resources for the further researches on agarwood formation mechanism, genome-assisted improvements and conservation biology of Aquilaria species.
**Corresponding Author:**	Haofu Dai, Ph.D<br><br>CHINA
**Corresponding Author Secondary Information:**	
**Corresponding Author's Institution:**	
**Corresponding Author's Secondary Institution:**	
**First Author:**	Xupo Ding
**First Author Secondary Information:**	
**Order of Authors:**	Xupo Ding<br>Wenli Mei<br>Qiang Lin

	Hao Wang
	Jun Wang
	Shiqing Peng
	Huiliang Li
	Jiahong Zhu
	Wei Li
	Pei Wang
	Huiqin Chen
	Wenhua Dong
	Dong Guo
	Caihong Cai
	Shengzhuo Huang
	Peng Cui
	Haofu Dai, Ph.D
**Order of Authors Secondary Information:**	
**Additional Information:**	
**Question**	**Response**
Are you submitting this manuscript to a special series or article collection?	No
**Experimental design and statistics**<br><br>Full details of the experimental design and statistical methods used should be given in the Methods section, as detailed in our Minimum Standards Reporting Checklist. Information essential to interpreting the data presented should be made available in the figure legends.<br><br>Have you included all the information requested in your manuscript?	Yes
**Resources**<br><br>A description of all resources used, including antibodies, cell lines, animals and software tools, with enough information to allow them to be uniquely identified, should be included in the Methods section. Authors are strongly encouraged to cite Research Resource Identifiers (RRIDs) for antibodies, model organisms and tools, where possible.	Yes

Powered by Editorial Manager® and ProduXion Manager® from Aries Systems Corporation

Have you included the information requested as detailed in our Minimum Standards Reporting Checklist?	
**Availability of data and materials**<br><br>All datasets and code on which the conclusions of the paper rely must be either included in your submission or deposited in publicly available repositories (where available and ethically appropriate), referencing such data using a unique identifier in the references and in the “Availability of Data and Materials” section of your manuscript.<br><br>Have you have met the above requirement as detailed in our Minimum Standards Reporting Checklist?	Yes

Powered by Editorial Manager® and ProduXion Manager® from Aries Systems Corporation

Manuscript Click here to download Manuscript Manuscript-20190808.docx

DATA NOTE

Genome sequence of agarwood tree *Aquilaria sinensis* (Lour.) Spreng: the first chromosome-level draft genome in the Thymelaeceae family

Xupo Ding[1, †], Wenli Mei[1, †], Qiang Lin[2 †], Hao Wang[1], Jun Wang[1], Shiqing Peng[3], Huiliang Li[3], Jiahong Zhu[3], Wei Li[1], Pei Wang[1], Huiqin Chen[1], Wenhua Dong[1], Dong Guo[3], Caihong Cai[1], Shengzhuo Huang[1], Peng Cui[2*], Haofu Dai[1, *]

[1] Hainan Engineering Research Center of Agarwood, Institute of Tropical Bioscience and Biotechnology, Chinese Academy of Tropical Agricultural Sciences, Rd. Xueyuan No.4, Haikou, 571101, China,

[2] Agricultural Genomics Institute, Chinese Academy of Agricultural Sciences, 518120 Shenzhen, Guangdong, China;

[3] Key Laboratory of Biology and Genetic Resources of Tropical Crops of Ministry of Agriculture and Rural Affairs, Institute of Tropical Bioscience and Biotechnology; Chinese Academy of Tropical Agriculture Sciences, Rd. Xueyuan No.4, Haikou, 571101, China

Correspondence address.

Peng Cui, Agricultural Genomics Institute, Chinese Academy of Agricultural Sciences, Rd. Pengfei No. 7, Shenzhen, 518120, China; Tel: +86-13828743816; E-mail: cuipeng@caas.cn;

Haofu Dai, Institute of Tropical Bioscience and Biotechnology, Chinese Academy of Tropical Agricultural Sciences, Rd. Xueyuan No.4, Haikou, 571101, China. Tel: +86-89866961869; E-mail: daihaofu@itbb.org.cn

[†]Contributed equally to this work.

Abstract

Backgroud: *Aquilaria sinensis* (Lour.) Spreng is one of important plant resources for producing agarwood in China. The agarwood collected from the wounded *Aquilaria* trees have been used for aromatic or medicinal purposes in these regions from the ancient time, whereas the mechanism underlying the formation of agarwood still remained poorly understood by a lack of accurate and high-quality genetics information. **Findings**: We report genomic architecture of *A.sinensis* by an integrated strategy combining with Nanopore sequencing, Illumina sequencing and Hi-C sequencing. The final genome was approximately 726.5 Mb, which reached a high level of continuity with a contig N50 of 1.1 Mb. We combined Hi-C data with the genome assembly to generate chromosome-level scaffolds. Eight super-scaffolds corresponding to the 8 chromosomes were assembled to a final size of 716.6 Mb, with a scaffold N50 of 88.78 Mb using 1,862 contigs. Benchmarking Universal Single-Copy Orthologs evaluation reveals that the genome completeness reaches 95.27%. The repeat sequences are accounted for 59.13% and the protein-coding genes are annotated for 29,203 in the entire genome. According to phylogenetic analysis using single-copy orthologous genes, we found that *A.sinensis* is closely related to *Gossypium hisutum* and *Theobroma cacao* from the Malvales order, and *A.sinensis* was diverged from their common ancestor approximately 53.18-84.37 million years ago. We also found a recent whole-genome duplication (WGD) event in *A.sinensis* genome. **Conclusions**: Here, we represent the first chromosome-level genome assembly and gene annotation of *A.sinensis*. This study would contribute to provide valuable genetic resources for the further researches on agarwood formation mechanism, genome-assisted improvements and conservation biology of *Aquilaria* species.

Keywods: *Aquilaria sinensis*; agarwood; chromosome-level genome assembly; Hi-C sequencing; annotation

Background information

Agarwood is fragrant resin-filled heartwood from the trees of the *Aquilaria* or *Gyrinops* genus and high-quality agarwood is more costly than gold in the international market [1,2]. Agarwood has been used as precious incense in Buddhist, Islamic and Hindu ceremonies, and also as the traditional medicine in Chinese therapies and Ayurveda [3]. Modern pharmacological and chemical studies have indicated that sesquiterpenoid and phenylethyl chromone derivatives are the principal compounds in agarwood, and many of them have potential pharmacological activities including neuroprotection, sedative, acetylcholinesterase inhibition, antioxidant, anti-bacterial and anti-inflammatory activities [4,5,6,7]. However, healthy *Aquilaria* trees hardly generate agarwood unless they were stimulated by various forms of injury or microbial infestation. In the wild, agarwood formation was usually considered related to natural factors such as wounded by wind, lighting, or gnawed by insects and fungi. [8,9]. Due to their medicinal and economic importance, the traditional methods were widely used for producing agarwood in Asia, such as chopping, nailing, holing, burning on the stem of *Aquilaria* trees or pruning the partial trunk [10], resulting the wild *Aquilaria* plants exploited excessively, and many of them considered as decreasing or endangered [11].

Aquilaria sinensis has been harvested and cultured for producing agarwood, which was used as Traditional Chinese Medicine (TCM) in China as early as the 7th century [11]. The morphological characteristics and agarwood of the *A.sinensis* are shown in Fig. 1. As the largest producer of agarwood in China, wild tree of *A.sinensis* is the endangered species circumscribed in the family of Thymelaeceae and has been listed in the IUCN red list from 1998 [11, 12]. The availability of agarwood is limited by the exhaustion of its time-consuming preparation and its plant sources. Although the expression of genes related to terpene synthesis or stress responses during agarwood formation have been described via transcriptome sequencing [2, 13, 14], the molecular mechanism of agarwood formation has remained unclear on account of lacking accurate genome information and genetic resources. Recently, we also founded that 2-(2-phenylethyl) chromone and its derivatives were the sign matters for agarwood formation in *A.sinensis* and their hypothetical biosynthetic pathway were further proposed [8]. With reduction of *A.sinensis* plant and increasing of agarwood requirement on the market, it is vital to interrogate genomic resource to explore the mechanism of agarwood formation and to accelerate the genome-assisted improvement in breeding systems.

Herein, we sequenced and assembled the genome of *Aquilaria sinensis* by hybrid approach using Illumina short reads, Oxford Nanopore long reads and Hi-C data. We reveal the genomic features of *Aquilaria sinensis*, including repeat sequence, gene annotation and evolution. This reference genome will provide the fundamental genetic information to elucidate the metabolic formation of agarwood and facilitate the genetic research of *Aquilaria* tree.

Data Description

Genomic DNA extraction and genome size estimation

An individual plant of cultivar *Aquilaria sinensis* (Lour.) Spreng was collected from Chengxi district (110°19′24.47″E, 19°59′7.57″N), Haikou, China. Healthy and fresh leaves after collection were frozen in liquid nitrogen immediately, followed by preservation at -80℃ in the laboratory prior to DNA extraction. High-molecular-weight plant genomic DNA was extracted from these leaves using a modified CTAB method [15]. The quality and quantity of the isolated DNA were checked by electrophoresis on a 0.75% agarose gel and a NanoDrop D-1000 spectrophotometer (NanoDrop Technologies, Wilmington, DE), and then were accurately quantified by Qubit technology. Subsequently, two libraries with insert lengths of 270 bp were constructed and 49.84 Gb raw data were generated using the standard protocol of Illumina Hiseq2500 platform, which were used for estimating the genome size of *A.sinensis* by the formula (genome size= [Num (total k-mer)-Num (erro k-mer)]/ average depth of k-mer) [16]. Finally, the genome size of A.*sinensis* was estimated as 773.3 Mb with the total number of 19-mer approximately to 3.71×10^{10} and the peak of 19-mer at the depth of 48 (Supplementary Fig. S1). The GC content of *A.sinensis* genome was 39.23%, which is considered a moderated GC content (Supplementary Fig. S2). Meanwhile, the heterozygosity of 0.32% and repeat content of 53.12% for *A.sinensis* genome were also estimated in this section.

Genomic sequencing and assembly using Nanopore long reads

One Nanopore 1D library was prepared following the Oxford Nanopore SQK-LSK 108 kit and GridION protocol [17]. Genomic DNA was first repaired and end prepped with NEBNext FFPE Repair Mix (New England Biolabs) and the NEBNext Ultra II End Repair/dA-Tailing Module (NEB). The DNA was then purified with AMPure XP beads (Beckmann Coulter) and ligated with sequencing adapters provided by ONT using concentrated T4 DNA ligase 2 M U ml^{-1} (NEB). After purification with AMPure XP beads (Beckman Coulter) using dilution buffer (ONT) and wash

buffer (ONT), the library was mixed with sequencing buffer (ONT) and library loading beads (ONT) and loaded on 16 flow cells (R9.4) of GridION X5 platform [18], generating 71.3 Gb raw DNA reads (roughly 100× coverage of the genome assembly). We obtained 4.8 million subreads (67.7 Gb in total) with an N50 read length of 21.29 kb and the longest read length of 935.06 kb after removing adaptor (Supplementary Table S1).

The clean long reads obtained from Nanopore were initially assembled by wtdbg version 1.3 (https://github.com/ruanjue/wtdbg) with parameters: wtdbg -t 60 -i Passed.fastq -o Sample -H -k 17 -S 1.01 -e 4. The iterative polishing was conducted thrice by Pilon version 1.22 (RRID:SCR_014731) [19] and BWA (RRID:SCR_010910) [20] with the default parameters. The Pilon program was also run with default parameters to fill gaps, fix bases, and correct local misassemblies. 99.26% of Illumina short reads can be able to align to the assembled genome (Supplementary Table S2). The primary draft genome assembly was 720 Mb with a contig N50 length of 1.1 Mb and the longest contig length of 11.9 Mb (Supplementary Table S3). The contig N50 of *A.sinensis* genome was much higher than other published medicinal plants genome assemblies (Supplementary Table S4).

Hi-C library construction and chromosome-scale assembly

Hi-C, derived from chromosome conformation capture technology, is a method that probes the three-dimensional architecture of whole genomes by coupling proximity-based ligation with massively parallel sequencing [23]. The Hi-C contract matrix has been widely used for assembly correction to generate chromosome-scale scaffolds. In this work, the genomic DNA used for Hi-C library was extracted from a fresh leaf sample of *A.sinensis* with the standard method. The crosslinked DNA from lysed cells was digested with Dpn II after cells fixed with formaldehyde. Sticky ends were biotin labeled and proximity ligated to form chimeric junctions and then physically sheared to a size of 300-500 bp. Chimeric fragments representing the original cross-linked and long-distance physical interactions were then processed into paired-end sequencing libraries after the polymerase chain reaction (PCR) amplification. The PCR cycling protocol was as the following with 95℃ for 5 minutes; cycled 18×; 4℃ for 30 seconds, 45℃ for 1 second, 70℃ for 20 seconds, and 98℃ for 30 seconds; held at 4℃. The products of PCR were purified according to the Hi-C protocol and then the purified DNA was sheared, end-repaired, adenylation tailed, and universal adapter ligated, and samples were indexed as described in the manufacturer's recommendations [24].

The whole genome Hi-C library was sequenced with 150bp paired-end (PE) sequencing on Illumina Hiseq 2500. A total of 714.27 million clean PE reads (~103.07 Gb, roughly 142× coverage of assembled genome) were generated after filtering adapters and low quantity reads with Fastp (version 0.12.6) [25]. By mapping the Hi-C data to the Nanopore-based assembly using bowtie2 (RRID:SCR_005476) [26], we found 93.49 million unique mapped paired-end reads and 62.89 million valid interaction pairs, which were respectively accounted for 26.18% and 17.61% in the clean data (Supplementary Table S5). We employed BWA and Lachesis software to align paired end reads and retain the reads aligned to 500 bp away from each restriction site [27]. According to the conduct of clustering, ordering and orienting to the assembly contigs, these sequences were divided into 8 chromosome clusters and scaffolded by using Lachesis software with tuned parameters (Supplementary Table S6, Fig. 2). Finally, heatmap of Hi-C interaction for finally assembly was shown by R program (Version 3.5.3) [28, 29].

Total 1,862 contigs were used for scaffolding by Hi-C data, which consequently generated 805 scaffolds. The Hi-C assisted chromosome-length scaffolds resulted in a final size of 716.6 Mb accounting for the 99.85% draft genome, which showed a high level of continuity with a contig N50 of 1.1 Mb and a scaffold N50 of 88.78 Mb. The final draft genome assembly of *A.sinensis* was 726.5 Mb (Supplementary Table S3). The anchor rate of contigs (>100 kb) to pseudochromosomes was attained up to the 98.63% based on the Hi-C assembly (Table 1). The scaffold N50 of *A.sinensis* genome was also superior to other published medicinal plant genome assemblies (Supplementary Table S4).

Genome quality evaluation

To evaluate the completeness of our assembly, we subjected the final assembled genome sequences to Benchmarking Universal Single-Copy Orthologs (BUSCO) version 3 (BUSCO. *Embryophyta* odb 10, RRID:SCR_015008) (BUSCO, *Embryophyta* odb 10) [30,31]. Overall, 95.27% of 1375 expected embryophyta genes were identified in our genome assembly as the complete and partial BUSCO profiles. Among these identified 1310 complete expected embryophyta genes, 1202 and 108 were identified as single copy and duplicated copies respectively (Supplementary Table S7).

Repeat sequences within the *A.sinensis* genome assembly

Transposable elements (TEs) and tandem repeats were identified with both homology-based

annotation and *de novo* methods. Consensus sequences of repetitive elements were *de novo* identified and classified using the software package RepeatModeler version 1.04 (RepeatModeler, RRID:SCR 015027) [32]. RepeatMask version 3.2.9 (RepeatMasker, RRID:SCR 012954) [32], RepeatProteinMasker [33] and TRF [34] were used to discover and identify repeats within the respective genomes. Furthermore, simple sequence repeat (SSR) in the *A.sinensis* genome were also classified with MISA (MISA, RRID:SCR 010765) [35].The results showed that *de novo* predicted repeats were more recently active than Repbase [36] predicted repeats (Supplementary Fig. S3). The identified repeat sequences in the *A.sinensis* genome assembly accounted for 59.13% and total length of those accounted for 425.87 Mb (Supplementary Table S8). In particular, the details showed that long terminal repeat (LTR) was the most abundant repeat type and that two non-LTR retrotransposons, short interspersed nuclear element (SINE) and long interspersed nuclear element (LINE) [37], had the lowest proportions in the final assemblies. In addition, 13.12% of repeat sequences could not be classified (Table 2). Total 367,251 SSRs are identified from the draft assembly in 675 scaffolds. Mononucleotides (64.71%), dinucleotides (18.19%), and trinucleotides (12.46%) comprised nearly 96% of SSRs in our assembly (Supplementary Table S9).

Gene predication and annotation

Three strategies were used for gene prediction. Augustus version 3.2.3 (Augustus, RRID:SCR 008417) [38], GlimmHmm [39] and GeneID (GeneID, RRID:SCR 002473) [40] were used for *ab initio* gene prediction, using model training based on CDS from *Corchorus olitorius* (COLO4_1.0) [41], *Durio zibethinus* (Duzib1.0) [42], *Gossypium hirsutum* (ASM98774v1) [43], *Herrania umbratica* (ASM216827v2) [44], *Theobroma cacao* (Cirollo_cocoa_geneoe_v2) [45] and *Arabidopsis thaliana* (TAIR10) [46]. GeneWise (GeneWise, RRID:SCR 015054) [47] and GeMoMa [48] were used for homology prediction. PASA (PASA, RRID:SCR 014656) [49] and Tophat (TopHat, RRID:SCR 013035) [50] were used for gene structural prediction based on EST and cDNA sequences. Finally, the total gene prediction was obtained from the union of these three strategies with EVM [49]and filtering the transposable elements with Transposon PSI (Transposon, RRID:SCR 001159) [51]. RNA-seq data of mixed tissues was mapped onto the reference genome using Bowtie2 [26] and GMAP (GMAP, RRID:SCR_008992) [52], respectively. The final annotation was composed of 29,203 genes models with an average of 3,177.62 bp transcripts and 1,114.16 bp coding sequence, and each gene contains 5.02 exon with the average length of 222.09

bp. The comparative information of genes from *A.sinensis* and six closely related plants was also calculated (Supplementary Table S10), including their distributions of CDS and gene length, exon and intron length, exon and intron number (Supplementary Fig. S4). Genes were characterized for their putative function by performing the Blastall [53] and KAAS [54] search of the peptide sequences against the Swiss-Prot (Swiss-Prot, RRID:SCR_002380) [55], NR [56], TrEMBL (TrEMBL, RRID:SCR_002380) [55], KEGG (the Kyoto Encyclopedia of Genes and Genomes, Orthology) database (KEGG, RRID:SCR_012773) [57], COG (Clusters of Orthologous Groups) database (COG, RRID:SCR_007273) [58] and the Gene Ontology (GO) database (GO, RRID:SCR_002811) [59]. Protein conservative models and motifs predication were used InterProScan version 5.2 (InterproScan, RRID:SCR_005829) [60]. Of these 29,203 protein-coding genes, 82.64% have functional annotation. Summary of hits of database research was as the following: Swiss-Prot (19,586; 67.07%), NR (24,097; 82.52%), TrEMBL(23,455; 80.32%), KEGG (8,494; 29.09%), COG (13,592; 46.54%), GO (14,019, 78.70%) and InterProScan (20,031; 68.59%) (Supplementary Table S11). In addition, we also identified 207 miRNAs, 34 rRNAs, 173 tRNAs and 1,173 snRNAs via Rfam non-coding RNA (ncRNA) database (Rfam, RRID:SCR 007891) [61], tRNAscan-SE (tRNAscan-SE, RRID:SCR 010835) [62] and RNAmmer [63]. The average length, total length and percentage of ncRNAs in *A.sinensis* genome were further assessed (Supplementary Table S12).

Gene family identification and phylogenetic tree construction

Whole protein coding genes sets from *A.sinensis* genome and other 12 representative plant genomes including *G.hirutum* (ASM98774v1), *A.thaliana* (TAIR10), *T.cacao* (Cirollo_cocoa_geneoe_v2), *Cephalotus follicularis* (Cfol_1.0), *Citrus clementina* (Citrus_clementina_v1.0), *Cucurbita pepo* (ASM280686v2), *Eucalyptus grandis* (Egrandis1_0), *Glycine max* (Glycine_max_v2.1), *Helianthus annuus* (HanXRQr 1.0), *Populus euphratica* (PopEup_1.0), *Quercus suber* (CorkOak 1.0),and *Vitis vinifera* (assembly 12X) were used to construct a global gene family classification with all-vs-all BLASTP($1e^{-5}$ cutoff, Blast+ v2.3.056) and OrthoMCL version 2.0.9 (Ortholog Groups of Protein Sequences, RRID: SCR_007839) [64] after removing the redundancy caused by alternative splicing variations and remaining the longest transcript of each gene. In our assembly, 21,955 genes were clustered into 13,713 gene families. 789 gene families and 7,248 genes were unique to *A.sinensis* (Fig.3a and Supplementary Table S13). Of these, 9,615 gene families were

clustered among *G.hirsutum* from Malvaceae, *C.olitorius* from Tiliaceae, *T.cacao* from Sterculiaceae and *A.thaliana* as the model plant from Cruciferae, whereas 804 predicted genes family were unique to *A.sinensis* genome (Fig. 3b). *G.hirsutum*, *C.olitorius* and *T.cacao* represent the typical family sequenced to date in Malvales and the Thymelaeceae family is also divided into Malvales order in APG IV [65].

Single-copy genes or the orphan genes with only one copy in the genome duplication and evolution of species are highly conserved, which are generally used for establishing genetic relationship and origin of species. Alignment of single-copy gene was performed with protein sequences by Mafft (Mafft, RRID: SCR_011811) [66], then the bias regions were filtering with the Gblocks (Gblocks, RRID: SCR_015945) [67] and the final CDSs were obtained for evolutional analyses with GTRGAMMA model in the RaxML (RaxML, RRID: SCR_006086) [68] methods. The bootstrap was 100 and *Helianthus annuus* from the asterids was the outgroup [69]. We constructed a phylogenetic tree and estimated the divergence time of 13 plants by 89 single-copy gene families with the MCMCTREE of PAML [70] (Supplementary Fig. S5) (Parameters: clock = 2, RootAge = <100.6, model = 7, BDparas = 1 1 0，kappa gamma = 6 2，alpha gamma = 1 1, rgene gamma = 2 3.18, sigma2 gamma = 1 1.3). The divergence time between *A.sinensis* and *A.thaliana* was estimated as 82.14 (67.63-93.99) million years ago (Mya), and the divergence time between *A.sinensis* and the common ancestor of *G.hisutum* and *T.cacao* from Malvales order was approximately 69.64 (53.18-84.37) million years ago (Fig. 3c and Supplementary Fig. S6).

Gene family expansion and contraction

Expansion and contraction of defining gene family is an important driver of physiological changes of metabolite constituents and species adaptation during plant evolution [71]. We determined expansion and contraction of orthologous gene families in *A.sinensis* genome by CAFÉ 2.2 (CAFÉ, RRID: SCR_005983) with default parameters[72]. We inferred 53 expansion families and 117 contraction families with *A.sinensis* genome after comparing 11855 gene families across all 13 species (Fig.3c). The gene family changes for all 13 species were summarized in Supplementary Table14. Using Blast2GO (B2G, RRID: SCR_005828), we found 53 expansion gene families enriched in 56 GO terms (Supplementary Fig. S7) and 117 contraction gene families enriched in 60 GO terms (Supplementary Fig. S8). Using the KEGG, the expansion gene families involved in the

pathways of plant circadian rhythm, tricarboxylic acid cycle, propanoate metabolism, Ribosome biogenesis and aminoacyl-tRNA biosynthesis (Supplementary Table S15), and the contraction gene families mapped pathways of starch/sucrose metabolism, sesquiterpenoid and triterpenoid biosynthesis and linoleic acid metabolism (Supplementary Table S16).

Whole-genome duplication

Whole genome duplications (WGDs), which also known as paleopolyploidization events, are widespread in plant lineages and represent a powerful evolutionary force for the development of novel gene functions and the emergence of new species [73]. We used MCScanX to identify the syntenic regions [74]. The longest isoform for each gene was selected for this exercise. The best five mutual hit of the BLASTP results in gene family analysis were used as input. Only the syntenic segments that have more than five gene pairs were considered for 4DTv calculation. Pairwise sequence was aligned using MUSCLE [75]. Raw 4DTv values were corrected for possible multiple transversions at the same site. Based on 4dTv distribution, a recent whole genome duplication (WGD) event is evident in the *A.sinenesis* genome and distinct from the scenarios in *A.thaliana*, *M.truncatula* and *V.vinifera* (Fig. 3d).

Conclusions

We *de novo* assembled a high-quality genome of *A.sinensis* in this study. The final assembly is approximately 726.5 Mb, which was slightly smaller than the k-mer estimated genome size of 773.3 Mb. The Hi-C data was used to revise the mis-assemblies and assign the contigs into a chromosome-scale scaffolds, and consequently generated an assembly with a high level of continuity with a contig N50 of 1.1 Mb and a scaffold N50 of 88.78 Mb. We also predicted 29,203 protein-coding genes from the final assembly and 82.64% (24,133 genes) of all protein-coding genes were annotated. We estimated that the divergence time between *A.sinensis* and its common ancestor with *G.hisutum* and *T.cacao* from Malvales order was approximately 53.18-84.37 million years ago. The genome of *A.sinensis* seems to experience a recent whole-genome duplication event after the K-T boundary [76]. Our assembly is also the first high-quality genome in the Thymelaeceae family. Considering the extinction and severely endangered status of natural *A.sinensis* tree populations due to stem heavily exploited for creating costly agarwood products, the genome assembly of *A.sinensis* will provide valuable information to aid the global conservation of these precious species and contribute to the understanding of the formation of agarwood.

Availability of supporting data

Supporting data and materials are available in the *GigaScience* GigaDB database (GigaDB, RRID:SCR 004002) [77], with the raw genomics sequences deposited in the NCBI Sequence Read Archive (SRA) database under the BioProject accession number PRJNA556948 and BioSample accession number SAMN12385133.

Additional files

S Fig.1 K-mer (k = 19) analysis for estimating the size of the *Aquilaria sinensis* genome.

S Fig.2 GC content and average sequencing depth of the Illumina sequencing data used for genome estimation.

S Fig.3 Distribution of sequence divergence rates of different TE types with Repbase (A) and *de nove* (B) methods in *Aquilaria sinensis* genome.

S Fig.4 Distribution of gene elements in *Aquilaria sinensis* genome and other six plant genome.

S Fig.5 Phylogenetic tree of 13 plant species including *Aquilaria sinensis.*

S Fig.6 Estimation of divergence time of 13 plant species investigated in the present study. The colored numbers on the nodes are the divergence time from present (million years ago). Numbers in the bracket indicate the 95% confidence interval of the divergence time.

S Fig.7 GO enrichment of expansion gene families in *Aquilaria* sinenesis genome.

S Fig.8 GO enrichment of contraction gene families in *Aquilaria* sinenesis genome.

S Table 1 Summary of Nanopore sequencing for *Aquilaria sinensis* genome.

S Table 2 Supporting of Illumina data for Nanopore data in *Aquilaria sinensis* genome assembled.

S Table 3 Statistics of the results of *Aquilaria sinensis* genome assembly before Hi-C mapping.

S Table 4 Comparisons of genome assemblies of medicinal plants based on descending Contig N50.

S Table 5 Summary of mapping status of Hi-C data

S Table 6 Statistics of pseudochromosomes length and scaffold number.

S Table 7 Statistics of BUSCO evolution for *Aquilaria sinensis* genome.

S Table 8 Statistics of repeat sequence in *Aquilaria sinensis* genome via different methods.

S Table 9 Statistics of SSRs in *Aquilaria sinensis* genome sequences.

S Table 10 Statistics of characters of gene models in *Aquilaria sinensis* and other six plant

genome.

S Table 11 The annotated genes of *Aquilaria sinensis* which can be functionally classified in each corresponding database.

S Table 12 Noncoding RNA annotation in the *Aquilaria sinensis* genome.

S Table 13 Summary of gene families among 13 plant species.

S Table 14 Summary of gene families changes among 13 species.

S Table 15 KEGG mapping of expansion gene families in *Aquilaria* sinenesis genome.

S Table 16 KEGG mapping of contraction gene families in *Aquilaria* sinenesis genome.

Abbreviation

IUCN: International Union for Conservation of Nature and Natural resources; BUSCO: Benchmarking Universal Single-Copy Orthologs; Hi-C: high-throughput chromosome conformation capture; TE: transposable element; EVM: EVidenceModeler;NR: PASA: Program to Assemble Spliced Alignments; NCBI non-redundant protein database; PCR: polymerase chain reaction; Pfam: protein families; TrEMBL: Translated EMBL-Bank; MYA: million years ago. CAFÉ: Computational Analysis of gene Family Evolution; MRCA: most recent common ancestor; K-T: Cretaceous-Tertiary.

Competing interests

The authors declare that they have no competing interests.

Funding

This work was supported by the Central Public-interest Scientific Institution Basal Research Fund for Chinese Academy of Tropical Agricultural Sciences (17CXTD-15), the National Natural Science Foundation of China (31870668) and the China Agriculture Research System (CARS-21). We are grateful to NextOmics Co., Ltd. (Wuhan, China) for providing technical help.

Author contributions

H.F.D., P.C. and W.L.M. conceptualized the research program. X.P.D., W.L.M. and S.Q.P. designed experiments and coordinated the program. S.Z.H. collected the sample and J.W. take the photos. H.L.L and J.H.Z. extracted the DNA. X.P.D., Q.L., H.W., P.C., W.L., H.Q.C., W.H.D., D.G. and C.H.C were partially involved either experiments or data analysis. X.P.D., W.L.M. and Q.L. wrote the manuscript. All authors read and approved the final manuscript.

References

1. Kumeta Y and Ito M. Characterization of α-humulene synthases responsible for the production of sesquiterpenes induced by methyl jasmonate in *Aquilaria* cell culture. Journal of Natural Medicines 2016;70(3): 452-459.

2. Xu Y, Zhang Z, Wang M, et al. Identification of genes related to agarwood formation: transcriptome analysis of healthy and wounded tissues of *Aquilaria sinensis*. BMC Genomics 2013;14(1): 227.

3. Naef R. The volatile and semi- volatile constituents of agarwood, the infected heartwood of *Aquilaria* species: a review. Flavour and Fragrance Journal 2011;26(2): 73-87.

4. Liao G, Mei WL, Kong FD, et al. 5, 6, 7, 8-Tetrahydro-2-(2-phenylethyl) chromones from artificial agarwood of *Aquilaria sinensis* and their inhibitory activity against acetylcholinesterase. Phytochemistry 2017;139: 98-108.

5. Hashim Y Z H Y, Kerr P G, Abbas P, et al. *Aquilaria* spp.(agarwood) as source of health beneficial compounds: A review of traditional use, phytochemistry and pharmacology. Journal of Ethnopharmacology 2016, 189: 331-360.

6. Ma CT, Eom T, Cho E, et al. Aquilanols A and B, macrocyclic humulene-type sesquiterpenoids from the agarwood of *Aquilaria malaccensis*. Journal of Natural Products 2017, 80(11): 3043-3048.

7. Yang L, Yang YL, Dong WH, et al. Sesquiterpenoids and 2-(2-phenylethyl) chromones respectively acting as α-glucosidase and tyrosinase inhibitors from agarwood of an *Aquilaria* plant. Journal of Enzyme Inhibition and Medicinal Chemistry 2019;34(1): 853-862.

8. Liao G, Dong W H, Yang J L, et al. Monitoring the chemical profile in agarwood formation within one year and speculating on the biosynthesis of 2-(2-phenylethyl) chromones. Molecules 2018;23(6): 1261.

9. Chhipa H, Chowdhary K, Kaushik N. Artificial production of agarwood oil in *Aquilaria* sp. by fungi: a review. Phytochemistry Reviews 2017;16(5): 835-860.

10. Azren P D, Lee S Y, Emang D, et al. History and perspectives of induction technology for agarwood production from cultivated Aquilaria in Asia: a review. Journal of forestry research 2019, 30(1): 1-11.

11. Harvey-Brown, Y. *Aquilaria sinensis*. The IUCN Red List of Threatened Species 2018. 2018; e.T32382A2817115. http://dx.doi.org/10.2305/IUCN.UK.2018-2.RLTS.T32382A2817115.en

12. Wang Y, Zhan D F, Jia X, et al. Complete chloroplast genome sequence of *Aquilaria sinensis* (Lour.) Gilg and evolution analysis within the Malvales order. Frontiers in Plant Science 2016;7: 280.

13. Wang X, Gao B, Liu X, et al. Salinity stress induces the production of 2-(2-phenylethyl) chromones and regulates novel classes of responsive genes involved in signal transduction in *Aquilaria sinensis* calli. BMC plant biology 2016;16(1): 119.

14. Wang X, Zhang Z, Dong X, et al. Identification and functional characterization of three type III polyketide

synthases from *Aquilaria sinensis* calli. Biochemical and biophysical research communications 2017;486(4): 1040-1047.

15. Porebski S, Bailey LG, Baum BR. Modification of a CTAB DNA extraction protocol for plants containing high polysaccharide and polyphenol components. Plant Molecular Biology Reporter 1997;15(1): 8-15.

16. Ding X, Mei W, Huang S, et al. Genome survey sequencing for the characterization of genetic background of *Dracaena cambodiana* and its defense response during dragon's blood formation. PloS ONE 2018;13(12): e0209258.

17. Leggett RM and Clark MD. A world of opportunities with nanopore sequencing. Journal of Experimental Botany 2017;68(20): 5419-5429.

18. Schmidt MHW, Vogel A, Denton AK, et al. *De novo* assembly of a new *Solanum pennellii* accession using nanopore sequencing. The Plant Cell 2017;29(10): 2336-2348.

19. Walker B J, Abeel T, Shea T, et al. Pilon: an integrated tool for comprehensive microbial variant detection and genome assembly improvement. PloS ONE 2014;9(11): e112963.

20. Li H, Durbin R. Fast and accurate long-read alignment with Burrows-Wheeler transform. Bioinformatics 2010; 26(5): 589-595.

21. Zhao Q, Yang J, Cui MY, et al. The Reference Genome Sequence of *Scutellaria baicalensis* Provides Insights into the Evolution of Wogonin Biosynthesis. Molecular Plant, 2019. Doi: 10.1016/j.molp.2019.04.002.

22. Wuyun T, Wang L, Liu H, et al. The hardy rubber tree genome provides insights into the evolution of polyisoprene biosynthesis. Molecular plant, 2018, 11(3): 429-442.

23. Lieberman-Aiden E, Van Berkum NL, Williams L, et al. Comprehensive mapping of long-range interactions reveals folding principles of the human genome. Science 2009;326(5950): 289-293.

24. Xu CQ, Liu H, Zhou SS, et al. Genome sequence of *Malania oleifera*, a tree with great value for nervonic acid production. GigaScience 2019;8(2): giy164.

25. Chen S, Zhou Y, Chen Y, et al. fastp: an ultra-fast all-in-one FASTQ preprocessor. Bioinformatics 2018;34(17): i884-i890.

26. Langmead B, Salzberg SL. Fast gapped-read alignment with Bowtie 2. Nature Methods 2012;9(4): 357.

27. Burton J N, Adey A, Patwardhan R P, et al. Chromosome-scale scaffolding of de novo genome assemblies based on chromatin interactions. Nature Biotechnology 2013;31(12): 1119.

28. R Core Team. R: A language and environment for statistical computing. 2019. https://www.R-project.org/.

29. Yin D, Ji C, Ma X, et al. Genome of an allotetraploid wild peanut *Arachis monticola*: a de novo assembly.

GigaScience, 2018;7(6): giy066.

30. Simão FA, Waterhouse RM, Ioannidis P, et al. BUSCO: assessing genome assembly and annotation completeness with single-copy orthologs. Bioinformatics 2015;31(19): 3210-3212.

31. Waterhouse RM, Seppey M, Simão FA, et al. BUSCO applications from quality assessments to gene prediction and phylogenomics. Molecular Biology and Evolution 2017;35(3): 543-548.

32. Bedell JA, Korf I, Gish W. MaskerAid: a performance enhancement to RepeatMasker. Bioinformatics 2000;16(11): 1040-1041.

33. Allred DB, Cheng A, Sarikaya M, et al. Three-dimensional architecture of inorganic nanoarrays electrodeposited through a surface-layer protein mask. Nano Letters 2008;8(5): 1434-1438.

34. Benson G. Tandem repeats finder: a program to analyze DNA sequences. Nucleic Acids Research 1999;27(2): 573-580.

35. Thiel T, Michalek W, Varshney R, et al. Exploiting EST databases for the development and characterization of gene-derived SSR-markers in barley (*Hordeum vulgare* L.). Theoretical and Applied Genetics 2003;106(3): 411-422.

36. Jurka J, Kapitonov V V, Pavlicek A, et al. Repbase Update, a database of eukaryotic repetitive elements[J]. Cytogenetic and Genome Research 2005;110(1-4): 462-467.

37. Yang L, Scott LA, Wichman HA. Tracing the history of LINE and SINE extinction in sigmodontine rodents. Mobile DNA 2019;10(1): 22.

38. Stanke M, Steinkamp R, Waack S, et al. AUGUSTUS: a web server for gene finding in eukaryotes. Nucleic Acids Research 2004;32(suppl_2): W309-W312.

39. Majoros WH, Pertea M, Salzberg SL. TigrScan and GlimmerHMM: two open source ab initio eukaryotic gene-finders. Bioinformatics 2004;20(16): 2878-2879.

40. Blanco E, Parra G, Guigó R. Using geneid to identify genes. Current Protocols in Bioinformatics 2007;18(1): 4.3. 1-4.3. 28.

41. Islam MS, Saito JA, Emdad EM, et al. Comparative genomics of two jute species and insight into fibre biogenesis. Nature Plants 2017;3(2): 16223.

42. Teh BT, Lim K, Yong CH, et al. The draft genome of tropical fruit durian (*Durio zibethinus*). Nature Genetics 2017;49(11): 1633.

43. Li F, Fan G, Lu C, et al. Genome sequence of cultivated Upland cotton (*Gossypium hirsutum* TM-1) provides insights into genome evolution. Nature Biotechnology 2015;33(5): 524.

44. *Herrania umbratica*. https://www.ncbi.nlm.nih.gov/genome/55117

45. Argout X, Martin G, Droc G, et al. The cacao Criollo genome v2. 0: an improved version of the genome for genetic and functional genomic studies. BMC Genomics 2017;18(1): 730.

46. Michael TP, Jupe F, Bemm F, et al. High contiguity *Arabidopsis thaliana* genome assembly with a single nanopore flow cell. Nature Communications 2018;9(1): 541.

47. Birney E, Durbin R. Using GeneWise in the Drosophila annotation experiment. Genome Research 2000;10(4): 547-548.

48. Keilwagen J, Hartung F, Grau J. GeMoMa: Homology-Based Gene Prediction Utilizing Intron Position Conservation and RNA-seq Data. Gene Prediction. Humana, New York, NY, 2019: 161-177.

49. Haas BJ, Salzberg SL, Zhu W, et al. Automated eukaryotic gene structure annotation using EVidenceModeler and the Program to Assemble Spliced Alignments. Genome Biology 2008;9(1): R7.

50. Trapnell C, Pachter L, Salzberg SL. TopHat: discovering splice junctions with RNA-Seq. Bioinformatics 2009;25(9): 1105-1111.

51. Yagi M, Kosugi S, Hirakawa H, et al. Sequence analysis of the genome of carnation (*Dianthus caryophyllus* L.). DNA Research 2013;21(3): 231-241.

52. Wu TD, Watanabe CK. GMAP: a genomic mapping and alignment program for mRNA and EST sequences. Bioinformatics 2005;21(9): 1859-1875.

53. Coordinators NR. Database resources of the national center for biotechnology information. Nucleic Acids Research 2017;45: D12.

54. Moriya Y, Itoh M, Okuda S, et al. KAAS: an automatic genome annotation and pathway reconstruction server[J]. Nucleic Acids Research 2007;35(suppl_2): W182-W185.

55. Boeckmann B, Bairoch A, Apweiler R, et al. The SWISS-PROT protein knowledgebase and its supplement TrEMBL in 2003. Nucleic Acids Research 2003;31(1): 365-370.

56. Yu K, Zhang T. Construction of customized sub-databases from NCBI-nr database for rapid annotation of huge metagenomic datasets using a combined BLAST and MEGAN approach. PLoS ONE 2013;8(4): e59831.

57. Kanehisa M, Furumichi M, Tanabe M, et al. KEGG: new perspectives on genomes, pathways, diseases and drugs. Nucleic Acids Research 2016;45(D1): D353-D361.

58. Kristensen DM, Kannan L, Coleman MK, et al. A low-polynomial algorithm for assembling clusters of orthologous groups from intergenomic symmetric best matches. Bioinformatics 2010;26(12): 1481-1487.

59. Gene Ontology Consortium. Gene ontology consortium: going forward. Nucleic Acids Research 2014;43(D1):

D1049-D1056.

60. Hunter S, Apweiler R, Attwood TK, et al. InterPro: the integrative protein signature database. Nucleic Acids Research 2008;37(suppl_1): D211-D215.

61. Griffiths-Jones S, Moxon S, Marshall M, et al. Rfam: annotating non-coding RNAs in complete genomes[J]. Nucleic Acids Research 2005;33(suppl_1): D121-D124.

62. Lowe TM, Eddy SR. tRNAscan-SE: a program for improved detection of transfer RNA genes in genomic sequence. Nucleic Acids Research 1997;25(5): 955-964.

63. Lagesen K, Hallin P, Rødland E A, et al. RNAmmer: consistent and rapid annotation of ribosomal RNA genes. Nucleic Acids Research 2007;35(9): 3100-3108.

64. Li L, Stoeckert CJ, Roos DS. OrthoMCL: identification of ortholog groups for eukaryotic genomes. Genome Research 2003;13(9): 2178-2189.

65. Chase MW, Christenhusz MJM, Fay MF, et al. An update of the Angiosperm Phylogeny Group classification for the orders and families of flowering plants: APG IV. Botanical Journal of the Linnean Society 2016;181(1): 1-20.

66. Katoh K, Standley D M. MAFFT multiple sequence alignment software version 7: improvements in performance and usability. Molecular Biology and Evolution 2013;30(4): 772-780.

67. Castresana, J. Selection of Conserved Blocks from Multiple Alignments for Their Use in Phylogenetic Analysis. Molecular Biology and Evolution 2000 17(4):540-552.

68. Stamatakis, A. RAxML-VI-HPC: maximum likelihood-based phylogenetic analyses with thousands of taxa and mixed models. Bioinformatics 2006;22(21):2688-2690.

69. Badouin H, Gouzy J, Grassa CJ, et al. The sunflower genome provides insights into oil metabolism, flowering and Asterid evolution. Nature 2017;546(7656): 148.

70. Yang Z. PAML 4: phylogenetic analysis by maximum likelihood. Molecular Biology and Evolution 2007;24(8): 1586-1591.

71. Denoeud F, Carretero-Paulet L, Dereeper A, et al. The coffee genome provides insight into the convergent evolution of caffeine biosynthesis. Science 2014;345(6201): 1181-1184.

72. De Bie T, Cristianini N, Demuth J P, et al. CAFE: a computational tool for the study of gene family evolution. Bioinformatics 2006;22(10): 1269-1271.

73. Otto, SP. The evolutionary consequences of polyploidy. Cell 2007; 131, 452–462.

74. Wang Y, Tang H, DeBarry J D, et al. MCScanX: a toolkit for detection and evolutionary analysis of gene synteny

and collinearity. Nucleic Acids Research 2012;40(7): e49-e49.

75. Edgar R C. MUSCLE: multiple sequence alignment with high accuracy and high throughput. Nucleic Acids Research 2004;32(5): 1792-1797.

76. Fawcett J A, Maere S, Van De Peer Y. Plants with double genomes might have had a better chance to survive the Cretaceous–Tertiary extinction event. Proceedings of the National Academy of Sciences 2009;106(14): 5737-5742.

77. Ding X, Mei W, Lin Q et al. Supporting data for "Genome sequence of agarwood tree *Aquilaria sinensis* (Lour.) Spreng: the first chromosome-level draft genome in the Thymelaeceae family" GigaScience Database 2019.

Figure legends

Figure 1: Morphological characteristic of *Aquilaria sinensis*. (a) mature tree; (b) flower; (c) fruit; (d) seed; (e) cracked seed; (f) agarwood generation; (g) agarwood. The pictures of numbered b, c, d and e were taken with stereoscopic fluorescence microscope (Olympus SZX16, Pittsburgh, PA) under the dark field. All the photos were taken by Dr. Jun Wang and processed by Dr. Xupo Ding.

Figure 2: Hi-C interaction matrix for *A.sinensis* genome assembly with 8 clusters.

Figure 3: Comparative genomic analysis of *Aquilaria sinensis* and other plant species. (a) Distribution of genes and gene families of 13 plant species we investigated. (b) A Venn diagram showing the distribution of shared gene families among the Malvales plants *Aquilaria sinensis* (agarwood), *Theobroma cacao* (cocoa), *Gossypium hirsutum* (cotton), *Corchorus olitorius* (jute) and the model plant *Arabidopsis thaliana* (Arabidopsis). (c) Divergence time estimation and gene families changes among 13 plant species. The black number at each node denotes estimated divergence time from present (million years ago). The blue number at the root (11885) denotes the total number of gene families predicted in the most recent common ancestor (MRCA) and the green/red numbers around each branch denote gene family gain/loss number. The red nodes indicate the known divergence time of Asterids and Rosids. (d) 4dTv distribution of whole genome duplication (WGD) in selected assemblies of *A.sinensis*, *A.thaliana*, *O.sativa*, *M.truncatula* and *V.vinifera*. 4dTv, transversion substitutions at four-fold degenerate sites.

Table legends

Table 1. Statistics of the final genome assembly for *Aquilaria sinensis.*

Table 2. Statistics of transposable elements in *Aquilaria sinensis* genome sequences.

 Click here to download Table Table 1.docx

Table 1. Statistics of the final genome assembly for *Aquilaria sinensis.*

Statistics Type	Scaffold Length (bp)	Scaffold Number	Contig Length (bp)	Contig Number
N50	1,904,330	90	1,096,099	155
N60	1,393,087	134	755,145	235
N70	1,061,733	194	506,948	352
N80	766,844	275	308,585	532
N90	442,131	398	153,880	859
Longest	13,647,514	1	11,913,571	1
Total	726,504,961	675	720,187,708	1,862
Length>=1kb	726,504,961	675	720,187,708	1,862
Length>=2kb	726,504,961	675	720,187,708	1,862
Length>=5kb	726,504,961	675	720,155,025	1,854

Click here to download Table Table 2.docx

Table 2. Statistics of transposable elements in *Aquilaria sinensis* genome sequences.

Type	Repbase TEs		Mips-REdat TEs		TE proteins		RepeatModeler		Combined TEs	
	Length (Mb)	% in genome	Length (Mb)	%in genome	Length (Mb)	% in Genome	Length (Mb)	% in genome	Length (Mb)	% in genome
DNA	13223408	1.84	1392136	0.19	10456270	1.45	28698131	3.98	38895471	5.4
LINE	2916904	0.41	253492	0.04	7680548	1.07	6394899	0.89	12239695	1.7
LTR	73748923	10.24	22973865	3.19	75336839	10.46	138348032	19.21	192609862	26.74
SINE	2232	0	1145	0	0	0	0	0	4539	0
Other	6189190	0.86	380555	0.05	1369337	0.19	0	0	87659087	12.17
Unknown	35443	0	0	0	0	0	124331790	17.26	94460416	13.12
Total	96116100	13.35	25001193	3.47	94842994	13.17	296679047	41.19	425869070	59.13

Figure 1. Morphological characteristic of Aquilaria sinensis.

Click here to download Figure Figure 1.tif

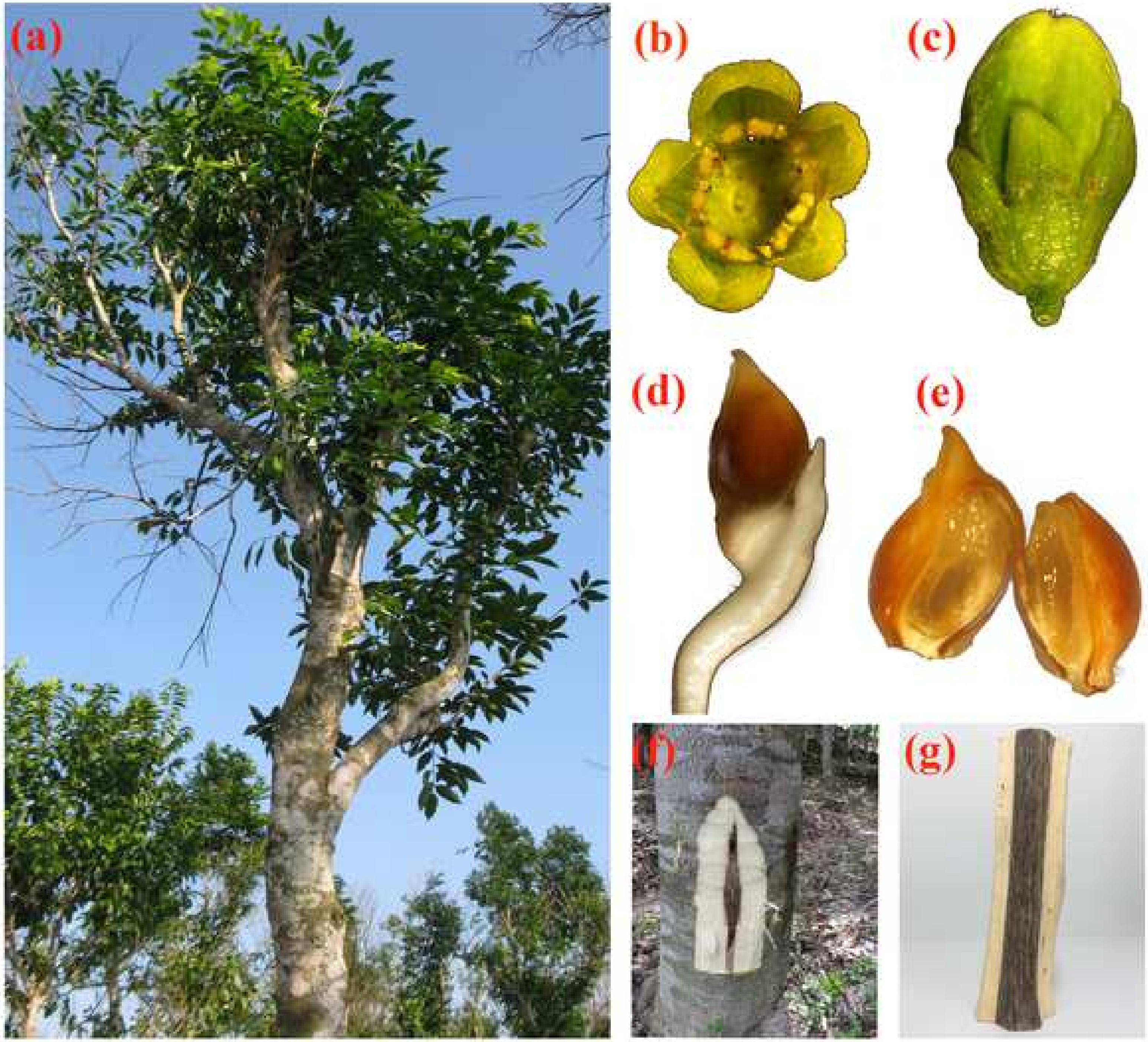

Figure 2. Hi-C interaction matrix for A.sinensis genome assembly with 8 clusters.

Click here to download Figure Figure 2.tif

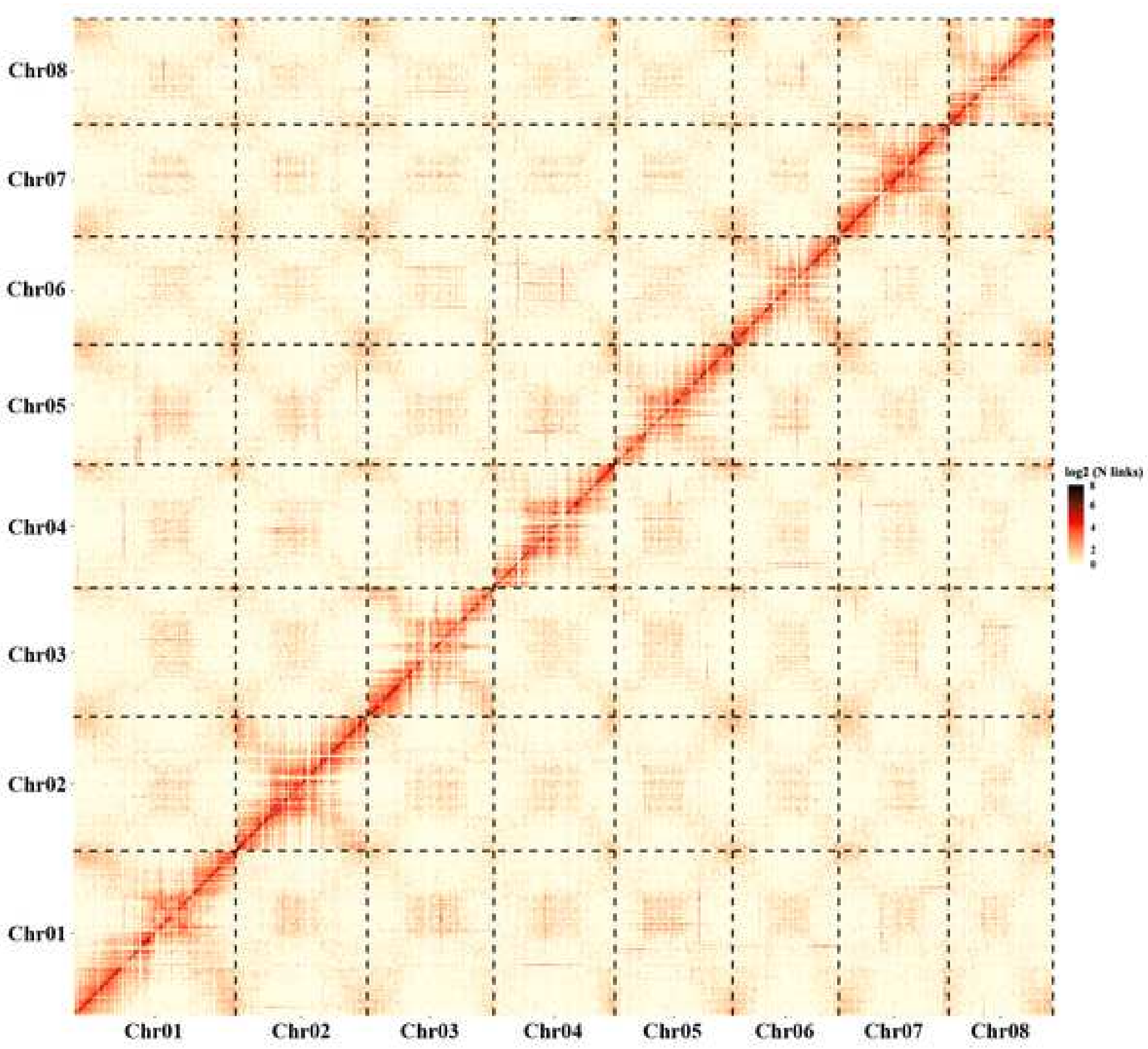

Figure 3. Comparative genomic analysis of Aquilaria sinensis and other plant species.

Click here to download Figure Figure 3.tif

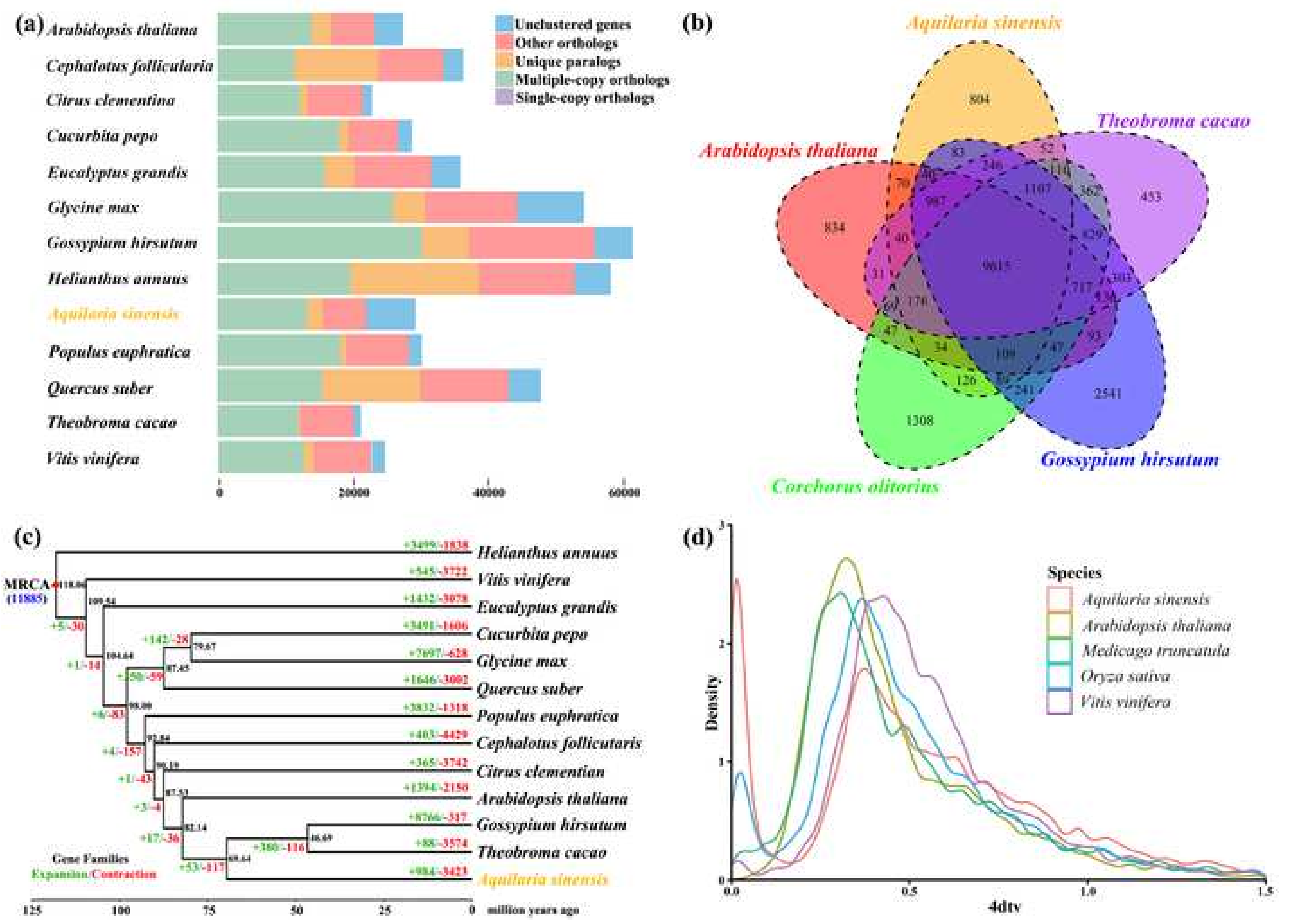

S Fig.1 K-mer (k = 19) analysis for estimating the size of the Aquilaria sinensis genome.

Click here to access/download
Supplementary Material
S Fig.1.tif

S Fig.2 GC content and average sequencing depth of the Illumina sequencing data used for genome estimation.

Click here to access/download
Supplementary Material
S Fig.2.tif

S Fig.3 Distribution of sequence divergence rates of different TE types with Repbase (A) and de nove (B) methods in Aquilaria

Click here to access/download
Supplementary Material
S Fig.3.tif

S Fig.4 Distribution of gene elements in Aquilaria sinensis genome
and other six plant genome.

Click here to access/download
Supplementary Material
S Fig.4.tif

S Fig.5 Phylogenetic tree of 13 plant species including Aquilaria sinensis.

Click here to access/download
Supplementary Material
S Fig.5.tif

S Fig.6 Estimation of divergence time of 13 plant species investigated in the present study.

Click here to access/download
Supplementary Material
S Fig.6.tif

S Fig.7 GO enrichment of expansion gene families in Aquilaria sinenesis genome.

Click here to access/download
Supplementary Material
S Fig.7.tif

S Fig.8 GO enrichment of contraction gene families in Aquilaria sinenesis genome.

Click here to access/download
Supplementary Material
S Fig.8.tif

S Table1-S Table16

Click here to access/download
Supplementary Material
S Table.xls

Cover letter

Click here to download Personal Cover Cover letter20190808.docx

Dear Editor,

We would like to submit the enclosed manuscript entitled " Genome sequence of agarwood tree *Aquilaria sinensis* (Lour.) Spreng: the first chromosome-level draft genome in the Thymelaeceae family ", which we wish to be considered for publication in *GigaScience*.

Agarwood is fragrant resin-filled heartwood from the trees of the *Aquilaria* or *Gyrinops* genus and high-quality agarwood is more costly than gold in the international market. For the inspiring and mystery smell, agarwood is considered as the 'King of Incense' and is the only incense used in all religions of Christianism, Catholicism, Buddhism, Islam, Taosim, and Hindusim. Agarwood was also used as the traditional medicine in Chinese therapies and Ayurveda. Now, more than 200 scientists in the world are focusing on chemical and biological researches of agarwood. Modern pharmacological and chemical studies have been indicated that the compounds in agarwood have potential pharmacological activities including neuroprotection, sedative, AchE inhibition, antioxidant, anti-bacterial and anti-inflammatory activities. Interestingly, only the stem of *Aquilaria* tree wounded by wind, light or gnawed by insects and infected by fungi have the possibility to produce small amount of agarwood. Due to their medicinal and economic importance, *Aquilaria* resources have been destroyed severely owing to overexploitation. Illuminating the molecular mechanism of its formation in *Aquilaria* plants might be effective for plant resource usage and agarwood production. For this purpose, our group launched the genome sequencing project of *Aquilaria* trees. The first species selected for whole genome sequencing is *Aquilaria sinensis*, which is the important original plants of agarwood production in China and southeast Asia from the 7th century. Our note present genomic characterization of *A.sinensis* via the data from whole genome sequencing on Nanopore GridION X5, including the genomic size, GC content, simple sequence repeat, gene predication and annotation and phylogenetic analysis. The Hi-C technology help us scaffolded those contigs to chromosomes-level, resulting in a genome with a high level of continuity, with a contig N50 of 1.1 Mb and a scaffold N50 of 88.78 Mb. We also predicted 29,203 protein-coding genes and 24,133 genes of all protein-coding genes were annotated. We found that the divergence time between *A.sinensis* and its common ancestor with *G.hisutum* and *T.cacao* from

Malvales order was approximately 53.18-84.37 million years ago. Gene family expansion/contraction and a recent whole-genome duplication (WGD) event were also analyzed in *A.sinensis* genome. Although this paper is about the initial results about *A.sinensis* genome, but the results presented here are anticipated to contribute on the progress of genetic studies of *Aquilaria* trees and may improve our understanding of agarwood formation. Furthermore, this is the first chromosome-level draft genome in the Thymelaeceae family.

We hereby certify that this paper consists of original, unpublished work which is not under consideration for publication elsewhere.

We hope your favorable consideration for publication to *GigaScience*.

Sincerely,

Prof. Haofu Dai[1] and Prof. Peng Cui[2]

[1] Hainan Engineering Research Center of Agarwood, Institute of Tropical Bioscience and Biotechnology, Chinese Academy of Tropical Agricultural Sciences, Rd. Xueyuan No.4, Haikou, 571101, China
Tel: +86-898-6696-1869; Email: daihaofu@itbb.org.cn;
[2] Agricultural Genomics Institute at Shenzhen, Chinese Academy of Agricultural Sciences, Rd. Pengfei No. 7, Shenzhen, 518120, China
Tel: +86-13828743816; E-mail: cuipeng@caas.cn.

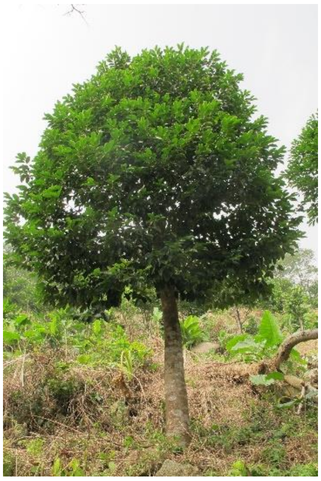

Aquilaria sinensis tree

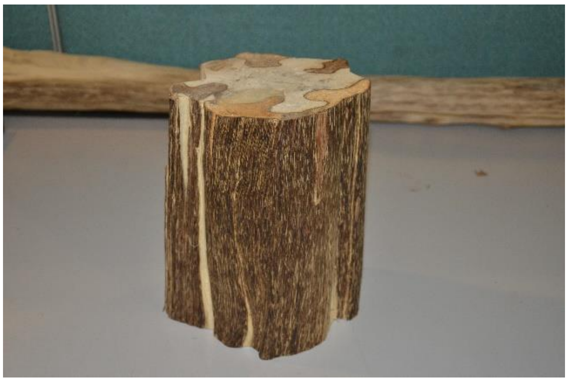

Agarwood in stem of *A.sinensis*
