# Supplementary material for: Genome sequence of the agarwood tree Aquilaria sinensis (Lour.) Spreng: the first chromosome-level draft genome in the Thymelaeceae family
Source: Gigascience. 2020 Mar 2;9(3):giaa013. doi: 10.1093/gigascience/giaa013 (PMC7050300; doi:10.1093/gigascience/giaa013)
Supplement: giaa013_GIGA-D-19-00288_Revision_1 [file giaa013_giga-d-19-00288_revision_1.pdf]

GigaScience

Genome sequence of agarwood tree Aquilaria sinensis (Lour.) Spreng: the first chromosome-level draft genome in the Thymelaeceae family

--Manuscript Draft--

**Manuscript Number:**	GIGA-D-19-00288R1
**Full Title:**	Genome sequence of agarwood tree Aquilaria sinensis (Lour.) Spreng: the first chromosome-level draft genome in the Thymelaeceae family
**Article Type:**	Data Note
**Funding Information:**	Central Public-interest Scientific Institution Basal Research Fund for Chinese Academy of Tropical Agricultural Sciences (17CXTD-15) — Prof. Wenli Mei<br>National Natural Science Foundation of China (31870668) — Prof. Wenli Mei<br>China Agriculture Research System (CARS-21) — Prof. Haofu Dai
**Abstract:**	Backgroud: Aquilaria sinensis (Lour.) Spreng is one of important plant resources for producing agarwood in China. The agarwood collected from the wounded Aquilaria trees have been used for aromatic or medicinal purposes in these regions from the ancient time, whereas the mechanism underlying the formation of agarwood still remained poorly understood by a lack of accurate and high-quality genetics information. Findings: We report genomic architecture of A.sinensis by an integrated strategy combining with Nanopore sequencing, Illumina sequencing and Hi-C sequencing. The final genome was approximately 726.5 Mb, which reached a high level of continuity with a contig N50 of 1.1 Mb. We combined Hi-C data with the genome assembly to generate chromosome-level scaffolds. Eight super-scaffolds corresponding to the 8 chromosomes were assembled to a final size of 716.6 Mb, with a scaffold N50 of 88.78 Mb using 1,862 contigs. Benchmarking Universal Single-Copy Orthologs evaluation reveals that the genome completeness reaches 95.27%. The repeat sequences are accounted for 59.13% and the protein-coding genes are annotated for 29,203 in the entire genome. According to phylogenetic analysis using single-copy orthologous genes, we found that A.sinensis is closely related to Gossypium hisutum and Theobroma cacao from the Malvales order, and A.sinensis was diverged from their common ancestor approximately 53.18-84.37 million years ago. Conclusions: Here, we represent the first chromosome-level genome assembly and gene annotation of A.sinensis. This study would contribute to provide valuable genetic resources for the further researches on agarwood formation mechanism, genome-assisted improvements and conservation biology of Aquilaria species.
**Corresponding Author:**	Haofu Dai, Ph.D<br><br>CHINA
**Corresponding Author Secondary Information:**	
**Corresponding Author's Institution:**	
**Corresponding Author's Secondary Institution:**	
**First Author:**	Xupo Ding
**First Author Secondary Information:**	
**Order of Authors:**	Xupo Ding<br>Wenli Mei<br>Qiang Lin<br>Hao Wang

Powered by Editorial Manager® and ProduXion Manager® from Aries Systems Corporation

	Jun Wang
	Shiqing Peng
	Huiliang Li
	Jiahong Zhu
	Wei Li
	Pei Wang
	Huiqin Chen
	Wenhua Dong
	Dong Guo
	Caihong Cai
	Shengzhuo Huang
	Peng Cui
	Haofu Dai, Ph.D
**Order of Authors Secondary Information:**	
**Response to Reviewers:**	Dear editor and reviewers:<br>Thanks for your letter and the reviewers' comments concerning our manuscript entitled "Genome sequence of agarwood tree Aquilaria sinensis (Lour.) Spreng: the first chromosome-level draft genome in the Thymelaeceae family" (ID: GIGA-S-19-00378). Those comments are all valuable and very helpful for revising and improving our paper, as well as the important guiding the significance to our future research. We have studied the comments carefully and have made correction which we hope meeting with approval. Especially we upload the relevant data to the NCBI-SRA and revised the language. Revised positions are marked in red in the manuscript with track changes. The main corrections in this revised manuscript and the responds to the reviewer comments are as the following and its word format (including table and figure) have been uploaded in the Supplementary materials too.<br><br>Response to the Reviewer #1:<br>Comment: The authors describe the first chromosome-level genome assembly of the agarwood tree. This work is of great interest because of the intensive use of the agar tree and the need of preservation of this tree. The authors have produced a big work to assemble and annotate the genome with good results.<br>1. Comment: The methods are well described but in line 204, the authors introduce RNA sequencing of mixed tissues. There is no description of the generation of those data (type of tissues used, RNA extraction method, RNA sequencing libraries preparation, sequencing depth ...). The data are not available in the NCBI Sequence Read Archive under the BioProject accession number given.<br>Response：The description of the RNA-seq data have been added and can be tracked with red marks in the revised manuscript from line 170 to line 179. Raw data have been submitted to PRJNA556948 in the SRA database of NCBI and its run number is SRR10276652.<br>2. Comment: The table 1 doesn't contain the statistics of the final assembly (same table than Supplementary table S3). What is the proportion of the gaps introduced after scaffolding with Hi-C?<br>Response：The new table 1 about the final assembly have been upload in the revised manuscript. The proportion of gaps is roughly 0.9% (~length of 6.4 Mb) in the final genome.<br>3. Comment: In the figure 3d, a table with the values of the picks could be added (minor issue).<br>Response：The values of the peaks have been added.<br>4. Comment: In the line 285, the authors used the HI-C data to correct the mis-assemblies. How many mis-assemblies were found? How have they been detected?<br>Response：In the initial Hi-C interactive heatmap, the regions of the interactive intensity between different pseudochromosomes higher than their intensity within same pseudochromosome were determined as the mis-assemblies. Then these regions were

Powered by Editorial Manager® and ProduXion Manager® from Aries Systems Corporation

corrected based on the interactive intensities of re-clustered after the contig divided into the equal length of 100 kb. The following table is the comparison of initial Hi-C assembly and the correct Hi-C assembly:

ChrInitial assemblyFinal assembly
SizeScaf NumSizeScaf Num
Chr01114,318,616151109,870,270166
Chr02108,239,0534999,503,77260
Chr0394,462,1337089,391,08391
Chr0489,950,3957288,784,93286
Chr0585,082,3757686,380,100112
Chr0680,447,6896984,956,75591
Chr0776,713,5048780,601,76289
Chr0876,239,0039477,134,862110
Total725,452,768668716,623,536805

5. Comment: Finally, the Hi-C sequencing data are not available in the NCBI Sequence Read Archive under the BioProject accession number given.
Response：The raw data of Hi-C sequencing have been submitted to PRJNA556948 in the SRA database of NCBI now and its run number is SRR10362483.

Response to Reviewer #2:
Comment: The genome sequence presented in your manuscript is a highly valuable resource for sustainable commercial exploitation and to help understanding the evolution of aromatic plants. You have reached a high level of continuity, obtainng a well-annotated chromosome-level assembly. In addition, the phylogenetic analysis is consistent with previous divergence time estimates and the analysis of gene families help to define the evolutionary context of the released the genome. However, before accepting the publication, I would like you to do a major revision of the manuscript and address some of my concerns.
1. Comment: In the background you state that A. sinensis have been included in the IUCN red list. Although that's true you don't mention the current assessment as VULNERABLE (by Harvey Brown, 2018). I think you should let this clear and be cautious at the end when you say it is extremely endangered, etc. Avoid overstatements, the species populations have suffered a dramatic decline in the last 10 years and wild populations are threatened, that's it.
Response：We have confirmed your information about the new assessment of A.sinensis and the description of endangered have been instead by “the species populations of A.sinensis have suffered a dramatic decline in the last 10 years and its wild populations are threatened” in the revision.
2. Comment: Please clarify which method you used to run the 19-mer analysis have you used your own method or software? Is it available for the community and reproducible?
Response：Using K-mer for estimating genome size is the routine approach based on sequencing data for estimating genomic characteristics. This GCE (genomic character estimator) was described in https://arxiv.org/abs/1308.2012 and adopted in other genome projects.
3. Comment: Regarding to this analysis how did you obtained the heterozygosity estimate of 0.32%? have you compared this with the results obtained with Genomescope, GCE or similar methods?
Response：The heterozygosity was estimated by GCE method with a Poisson model according to the same paper of genome size estimation. The heterozygosity value is various with different softwares. The Genome Scope used a mixed model and shown that the heterozygosity of A.sinensis is 0.6% and KAT show the number of 0.34%. We replaced the number with Genomescope result according comparative description in Genomescope’s paper
https://academic.oup.com/bioinformatics/article/33/14/2202/3089939.
4. Comment: I assume the HiSeq2500 was used to sequence two pair-end libraries and, by comparing sequence and k-mer coverage, the read length was 75bp. Please, specify if the library was paired or single end and what was the read length.
Response：The library was pair-end and its read length is 150 bp. This information has been revised in this current manuscript.
5. Comment: Line 122 - you wrote "We obtained 4.8 million subreads" however,

Powered by Editorial Manager® and ProduXion Manager® from Aries Systems Corporation

nanopore produced long reads and the term subread is used only for pacbio technology. I think this is wrong and should be corrected.
Response：The subreads is instead by nanopore long reads.
6. Comment: Nanopore sequence data: I would also like to know what was the mean accuracy of the reads. The reads that pass the filter are the ones having an average quality of at least 7, right?
Response：It is right, the mean qscore is greater than 7 (>7).
7. Comment: 133 - well, the comparison with other genomes looks very good. However, I suggest adding some comparison to the contig-level assembly for A.sinensis obtained with Pacbio (GCA_005392925.1 SCBG_Asin_1.0) and also A.agallocha (GCA_000696445.1). Mentioning these assemblies will look fair and also will highlight the higher contiguity of the one you present here.
Response：Two contig level assemblies for Aquilaria species have been detailed in the Table S4. The assembly derived from PacBio platform have not been published and the specific details could be got from the NCBI, so we just list N50 of contig in the compared table.
8. Comment: P.129. while polishing with pilon you haven't try to fix indels, why is this? Knowing that nanopore have recurrent errors in homopolymer tracks, I think will be an obvious thing to do. I am curious to know the reason.
Response: In Pilon software, the parameter of "fix bases" included the snps and indels. We specified it in the revised manuscript and the fix parameter has been listed as following:

Pilon --fix parameter：

```
--fix fixlist
 A comma-separated list of categories of issues to try to fix:
   "snps": try to fix individual base errors;
   "indels": try to fix small indels;
   "bases": shorthand for "snps" and "indels" (for back compatibility);
```

9. Comment: Regarding to the preprocessing of Hi-C reads. Does Fastp merely remove the adaptors? Other protocols, such as Arima Hi-C Mapping pipeline, try to remove some sequence at the ligation junction and keep the 5' ends to avoid the inclusion of "chimeric" reads…
Response：Fastp removed low quality reads and adaptors. For "chimeric" reads, this Hi-C pipeline is built on the basis of Hi-C pro software. Hi-C pro can detect all types of reads and get valid reads for downstream Hi-C analysis.
10. Comment: The assembly has a fairly high gene completeness as estimated by BUSCO. The mapping rate of the pair end reads reflects sequence quality. However, I would like you to perform some additional evaluations of the assembly such as a KAT stacked histogram, that reflect the efficiency and completeness of the assembly process, having into account the genome architecture (https://kat.readthedocs.io/en/latest/).
Response： The estimated assembly completeness is 90.08% in following 27-mer stacked histogram by KAT tools. Black square labeled the distribution of k-mers present in illumine paired-end data but absent in the assembly. Red labeled K-mers present in the read set and once in the assembly. Mainly of black fall into the regions of low quality and heterozygous (half of homozygous peak in x-axis).
11. Comment: I would also like you to include a link to the assembly and annotation in a public repository (this will be very important for the scientific community). In fact, I would like to have a look at the genome myself. In the manuscript you merely give access to the raw reads.
Response：The assembly, annotation and other related files of A.sinensis genome have been uploaded to GigaDB private FTP area for reviewers within peer review stage. The Gigascience will release them to GigaDB if the manuscript were published. Actually, we are constructing a professional public repository about Aquilaria species, including their genomics, transcriptome and metabolism, especially for their natural products. We will let you know when our database was online in the future.
12. Comment: In the supplementary table 2: what are exactly "properly mapped" reads? Do you mean "properly paired"? After looking at ENA it seems that the illlumina is a single end library…I am a bit confused with the term "properly mapped".
Response：The illumine sequence was based on the paired-end library, we have sent this mistake to the submission staff of NCBI for correction.
13. Comment: Please correct some typos: you wrote "Predication" several times (line 193, line 216…) but the correct word is PREDICTION, (i.e. Gene Prediction). Also

"sinenensis" (instead of "sinensis") or "de nove" (instead of de novo) appear later.
Response：These typos have been corrected in the revised manuscript.
14. Comment: Sentence in lines 227-235 will read much better by starting with "After removing the redundancy caused by alternative splicing variations and retaining the longest transcript of each gene, whole protein coding genes sets….OrthoMCL…[64]."
Response：This good suggestion has been adopted in the revised manuscript.
15. Comment: Please revise the English of this last pages (lines 238-296,) (specially for the last two pages, that seem to have been written on a rush)
Response：These paragraphs were revised in the revised manuscript.
16. Comment: Line 246 Gblocks filters "poorly aligned or highly divergent sites" from the alignment. I found "bias(ed) regions", as you wrote, a bit weird.
Response：We have accepted this more professional description in the revised manuscript.
17. Comment: Rather than contraction and expansion families I suggest to call them "contracted" and "expanded)
Response：All the contraction and expansion before families or gene families were replaced by contracted or expanded in the revised manuscript.
18. Comment: YOU SHOULD IMPROVE THE IMAGE RESOLUTION OF THE FIGURES the image resolution of the Figures in the manuscript (quality is very poor and numbers are often hard to read) What happened to Figure3c? in fact the supplementary images have much better quality.
Response：The image resolution of all the original figures in manuscript is 508 ×508 dpi. When they were inserted into word (.doc), their definition in generating PDF were much better than coalescing them directly after the manuscript.
19. Comment: I found the Whole genome duplication analyses weak and speculative. To my knowledge the 4DTV ratios can be used to date some evolutionary events, such as hybridization of gene family expansions/contractions. In your case the closest species to A. sinensis have very different genome sizes, with cocoa being almost half of sinensis (~300Mb) and Cotton with almost 3 times the sinensis genome size 2.2Gb. Have you found evidences of a ~300Mb of the agarwood genome being duplicated? Unless you have a more clear analysis of genome size evolution I suggest you to remove this from the manuscript?
Response：As your suggestion, we narrow down this section to “4DTv analysis” and shown the peak value of distribution. A.sinensis is the only species with chromosome-level assembly in Familia Thymelaeaceae. The cross-Familia comparison cannot fully reveal the diversity in plant genome size.
20. Comment: It would be good if you compare the divergence time estimates you have obtained with those found by other authors. By checking www.timetree.org I have confirmed that your estimate lay between the Confidence Intervals obtained by comparing several studies. It makes sense, to discuss a little bit your results or at least say that they are in concordance with previous studies.
Response：The divergence time of Asterids and Rosids, G.hisutum and T.cacao obtained in our analysis were compare with the previous studies about calibration divergence time in the revised manuscript.
21. Comment: A question about the biology of the plant. In your analyses, have you found any relationship between the immune genes and those involved in the production of agarwood or aromatic resin?
Response：This good suggestion might guide the significance to our future research. Immune gene is very important for plant defense response. But actually, we are focused on the genes about the terpene and flavonoid synthesis in the current stage, especially about the chromone synthesis, which is the sign matters for agarwood formation in A.sinensis. In addition, plant defense related genes were also concerned in our studies, the genes involved in abscisic acid and salicylic acid synthesis and regulation were correlative with contents of chromone in regions of agarwood formation. The ABA and SA also could induced the agarwood formation after they were injected into the stem of A.sinensis plants. According to the quantity, NB-LRRs and WD-40 were frequently presented in the DEG and annotation files. The expression trends of some NB-LRRs and WD-40 genes were highly correlative with agarwood formation based on time gradient. The pattern mentioned above from our previous transcriptome dataset without biological replicates (just five stages and each stage with mixture samples. because the RNA in the stem of agarwood formation was hardly accumulated), only part of them were verified with qPCR. Out further effort will focus on the LCM-based transcriptomics study.

Powered by Editorial Manager® and ProduXion Manager® from Aries Systems Corporation

	We tried our best to improve the manuscript and made some changes in the revised manuscript. These changes will not influence the content and framework of the paper. And here we did not list the changes but marked in red in revised paper. We appreciated for you and the reviews warm work earnestly, and hope that the correction will meet with approval. Once again, thanks for your comments and suggestion.<br><br>Sincerely,<br><br>Haofu Dai<br><br>Hainan Engineering Research Center of Agarwood, Institute of Tropical Bioscience and Biotechnology, Chinese Academy of Tropical Agricultural Sciences, Rd. Xueyuan No.4, Haikou, 571101, China<br>Tel: +86-898-6696-1869<br>Email: daihaofu@itbb.org.cn
**Additional Information:**	
**Question**	**Response**
Are you submitting this manuscript to a special series or article collection?	No
**Experimental design and statistics**<br><br>Full details of the experimental design and statistical methods used should be given in the Methods section, as detailed in our Minimum Standards Reporting Checklist. Information essential to interpreting the data presented should be made available in the figure legends.<br><br>Have you included all the information requested in your manuscript?	Yes
**Resources**<br><br>A description of all resources used, including antibodies, cell lines, animals and software tools, with enough information to allow them to be uniquely identified, should be included in the Methods section. Authors are strongly encouraged to cite Research Resource Identifiers (RRIDs) for antibodies, model organisms and tools, where possible.<br><br>Have you included the information requested as detailed in our Minimum Standards Reporting Checklist?	Yes

Powered by Editorial Manager® and ProduXion Manager® from Aries Systems Corporation

**Availability of data and materials**<br><br>All datasets and code on which the conclusions of the paper rely must be either included in your submission or deposited in publicly available repositories (where available and ethically appropriate), referencing such data using a unique identifier in the references and in the "Availability of Data and Materials" section of your manuscript.<br><br>Have you have met the above requirement as detailed in our Minimum Standards Reporting Checklist?	Yes

Powered by Editorial Manager® and ProduXion Manager® from Aries Systems Corporation

Revised Manuscript
Click here to view linked References
Click here to access/download;Manuscript;Revised Manuscript.docx

DATA NOTE

Genome sequence of agarwood tree *Aquilaria sinensis* (Lour.) Spreng: the first chromosome-level draft genome in the Thymelaeceae family

Xupo Ding[1, †], Wenli Mei[1, †], Qiang Lin[2 †], Hao Wang[1], Jun Wang[1], Shiqing Peng[3], Huiliang Li[3], Jiahong Zhu[3], Wei Li[1], Pei Wang[1], Huiqin Chen[1], Wenhua Dong[1], Dong Guo[3], Caihong Cai[1], Shengzhuo Huang[1], Peng Cui[2*], Haofu Dai[1, *]

[1] Hainan Engineering Research Center of Agarwood, Institute of Tropical Bioscience and Biotechnology, Chinese Academy of Tropical Agricultural Sciences, Rd. Xueyuan No.4, Haikou, 571101, China,

[2] Guangdong Laboratory of Lingnan Modern Agriculture, Shenzhen; Genome Analysis Laboratory of the Ministry of Agriculture; Agricultural Genomics Institute at Shenzhen, Chinese Academy of Agricultural Sciences, Rd. Pengfei No. 7, Shenzhen, 518120, China

[3] Key Laboratory of Biology and Genetic Resources of Tropical Crops of Ministry of Agriculture and Rural Affairs, Institute of Tropical Bioscience and Biotechnology; Chinese Academy of Tropical Agriculture Sciences, Rd. Xueyuan No.4, Haikou, 571101, China

Correspondence address.

Peng Cui, Agricultural Genomics Institute at Shenzhen, Chinese Academy of Agricultural Sciences, Rd. Pengfei No. 7, Shenzhen, 518120, China; Tel: +86-13828743816; E-mail: cuipeng@caas.cn; Haofu Dai, Institute of Tropical Bioscience and Biotechnology, Chinese Academy of Tropical Agricultural Sciences, Rd. Xueyuan No.4, Haikou, 571101, China. Tel: +86-89866961869; E-mail: daihaofu@itbb.org.cn

[†]Contributed equally to this work.

Abstract

Backgroud: *Aquilaria sinensis* (Lour.) Spreng is one of important plant resources for producing agarwood in China. The agarwood collected from the wounded *Aquilaria* trees have been used for aromatic or medicinal purposes in these regions from the ancient time, whereas the mechanism underlying the formation of agarwood still remained poorly understood by a lack of accurate and high-quality genetics information. **Findings**: We report genomic architecture of *A.sinensis* by an integrated strategy combining with Nanopore sequencing, Illumina sequencing and Hi-C sequencing. The final genome was approximately 726.5 Mb, which reached a high level of continuity with a contig N50 of 1.1 Mb. We combined Hi-C data with the genome assembly to generate chromosome-level scaffolds. Eight super-scaffolds corresponding to the 8 chromosomes were assembled to a final size of 716.6 Mb, with a scaffold N50 of 88.78 Mb using 1,862 contigs. Benchmarking Universal Single-Copy Orthologs evaluation reveals that the genome completeness reaches 95.27%. The repeat sequences are accounted for 59.13% and the protein-coding genes are annotated for 29,203 in the entire genome. According to phylogenetic analysis using single-copy orthologous genes, we found that *A.sinensis* is closely related to *Gossypium hisutum* and *Theobroma cacao* from the Malvales order, and *A.sinensis* was diverged from their common ancestor approximately 53.18-84.37 million years ago. **Conclusions**: Here, we represent the first chromosome-level genome assembly and gene annotation of *A.sinensis*. This study would contribute to provide valuable genetic resources for the further researches on agarwood formation mechanism, genome-assisted improvements and conservation biology of *Aquilaria* species.

Keywods: *Aquilaria sinensis*; agarwood; chromosome-level genome assembly; Hi-C sequencing; annotation

Background information

Agarwood is fragrant resin-filled heartwood from the trees of the *Aquilaria* or *Gyrinops* genus and high-quality agarwood is more costly than gold in the international market [1,2]. Agarwood has been used as precious incense in Buddhist, Islamic and Hindu ceremonies, and also as the traditional medicine in Chinese therapies and Ayurveda [3]. Modern pharmacological and chemical studies have indicated that sesquiterpenoid and phenylethyl chromone derivatives are the principal compounds in agarwood, and many of them have potential pharmacological activities including neuroprotection, sedative, acetylcholinesterase inhibition, antioxidant, anti-bacterial and anti-inflammatory activities [4,5,6,7]. However, healthy *Aquilaria* trees hardly generate agarwood unless they were stimulated by various forms of injury or microbial infestation. In the wild, agarwood formation was usually considered related to natural factors such as wounded by wind, lighting, or gnawed by insects and fungi. [8,9]. Due to their medicinal and economic importance, the traditional methods were widely used for producing agarwood in Asia, such as chopping, nailing, holing, burning on the stem of *Aquilaria* trees or pruning the partial trunk [10], resulting the wild *Aquilaria* plants exploited excessively, and many of them considered as decreasing or endangered [11].

Aquilaria sinensis has been harvested and cultured for producing agarwood, which was used as Traditional Chinese Medicine (TCM) in China as early as the 7th century [11]. The morphological characteristics and agarwood of the *A.sinensis* are shown in Fig. 1. As the largest producer of agarwood in China, the species populations of *A.sinensis* have suffered a dramatic decline in the last 10 years and its wild populations are threatened [11, 12]. The availability of agarwood is limited by the exhaustion of its time-consuming preparation and its plant sources. Although the expression of genes related to terpene synthesis or stress responses during agarwood formation have been described via transcriptome sequencing [2, 13, 14], the molecular mechanism of agarwood formation has remained unclear on account of lacking accurate genome information and genetic resources. Recently, we also founded that 2-(2-phenylethyl) chromone and its derivatives were the sign matters for agarwood formation in *A.sinensis* and their hypothetical biosynthetic pathway were further proposed [8]. With reduction of *A.sinensis* plant and increasing of agarwood requirement on the market, it is vital to interrogate genomic resource to explore the mechanism of agarwood formation and to accelerate the genome-assisted improvement in breeding systems.

Herein, we sequenced and assembled the genome of *Aquilaria sinensis* by hybrid approach using

Illumina short reads, Oxford Nanopore long reads and Hi-C data. We reveal the genomic features of *Aquilaria sinensis*, including repeat sequence, gene annotation and evolution. This reference genome will provide the fundamental genetic information to elucidate the metabolic formation of agarwood and facilitate the genetic research of *Aquilaria* tree.

Data Description

Genomic DNA extraction and genome size estimation

An individual plant of cultivar *Aquilaria sinensis* (Lour.) Spreng was collected from Chengxi district (110°19′24.47″E, 19°59′7.57″N), Haikou, China. Healthy and fresh leaves after collection were frozen in liquid nitrogen immediately, followed by preservation at -80℃ in the laboratory prior to DNA extraction. High-molecular-weight plant genomic DNA was extracted from these leaves using a modified CTAB method [15]. The quality and quantity of the isolated DNA were checked by electrophoresis on a 0.75% agarose gel and a NanoDrop D-1000 spectrophotometer (NanoDrop Technologies, Wilmington, DE), and then were accurately quantified by Qubit technology. Subsequently, 150bp paired-end (PE) libraries with insert lengths of 270 bp were constructed and 49.84 Gb raw data were generated using the standard protocol of Illumina Hiseq2500 platform, which were used for estimating the genome size of *A.sinensis* by the formula (genome size= [Num (total k-mer)-Num (erro k-mer)]/ average depth of k-mer) [16, 17]. Finally, the genome size of A.*sinensis* was estimated as 773.3 Mb with the total number of 19-mer approximately to 3.71×10^{10} and the peak of 19-mer at the depth of 48 (Supplementary Fig. S1). The GC content of *A.sinensis* genome was 39.23%, which is considered a moderated GC content (Supplementary Fig. S2). Meanwhile, the heterozygosity of 0.6% and repeat content of 53.12% for *A.sinensis* genome were also estimated in this section[18].

Genomic sequencing and assembly using Nanopore long reads

One Nanopore 1D library was prepared following the Oxford Nanopore SQK-LSK 108 kit and GridION protocol [19]. Genomic DNA was first repaired and end prepped with NEBNext FFPE Repair Mix (New England Biolabs) and the NEBNext Ultra II End Repair/dA-Tailing Module (NEB). The DNA was then purified with AMPure XP beads (Beckmann Coulter) and ligated with sequencing adapters provided by ONT using concentrated T4 DNA ligase 2 M U ml^{-1} (NEB). After purification with AMPure XP beads (Beckman Coulter) using dilution buffer (ONT) and wash

buffer (ONT), the library was mixed with sequencing buffer (ONT) and library loading beads (ONT) and loaded on 16 flow cells (R9.4) of GridION X5 platform [20], generating 71.3 Gb raw DNA reads (roughly 100× coverage of the genome assembly). We obtained 4.8 million nanopore long reads (67.7 Gb in total) with an N50 read length of 21.29 kb and the longest read length of 935.06 kb after removing adaptor (Supplementary Table S1).

The clean long reads obtained from Nanopore were initially assembled by wtdbg version 1.3 (https://github.com/ruanjue/wtdbg) with parameters: wtdbg -t 60 -i Passed.fastq -o Sample -H -k 17 -S 1.01 -e 4. The iterative polishing was conducted thrice by Pilon version 1.22 (RRID:SCR_014731) [21] and BWA (RRID:SCR_010910) [22] with the default parameters. The Pilon program was also run with default parameters to fill gaps, fix bases (including SNPs and indels), and correct local misassemblies. 99.26% of Illumina short reads can be able to align to the assembled genome (Supplementary Table S2). The primary draft genome assembly was 720 Mb with a contig N50 length of 1.1 Mb and the longest contig length of 11.9 Mb (Supplementary Table S3). The contig N50 of *A.sinensis* genome was much higher than other published medicinal plants genome assemblies (Supplementary Table S4).

Hi-C library construction and chromosome-scale assembly

Hi-C, derived from chromosome conformation capture technology, is a method that probes the three-dimensional architecture of whole genomes by coupling proximity-based ligation with massively parallel sequencing [23]. The Hi-C contract matrix has been widely used for assembly correction to generate chromosome-scale scaffolds. In this work, the genomic DNA used for Hi-C library was extracted from a fresh leaf sample of *A.sinensis* with the standard method. The crosslinked DNA from lysed cells was digested with Dpn II after cells fixed with formaldehyde. Sticky ends were biotin labeled and proximity ligated to form chimeric junctions and then physically sheared to a size of 300-500 bp. Chimeric fragments representing the original cross-linked and long-distance physical interactions were then processed into paired-end sequencing libraries after the polymerase chain reaction (PCR) amplification. The PCR cycling protocol was as the following with 95℃ for 5 minutes; cycled 18×; 4℃ for 30 seconds, 45℃ for 1 second, 70℃ for 20 seconds, and 98℃ for 30 seconds; held at 4℃. The products of PCR were purified according to the Hi-C protocol and then the purified DNA was sheared, end-repaired, adenylation tailed, and universal

adapter ligated, and samples were indexed as described in the manufacturer's recommendations [24]. The whole genome Hi-C library was sequenced with 150bp paired-end (PE) sequencing on Illumina Hiseq 2500. A total of 714.27 million clean PE reads (~103.07 Gb, roughly 142× coverage of assembled genome) were generated after filtering adapters and low quantity reads with Fastp (version 0.12.6) [25]. By mapping the Hi-C data to the Nanopore-based assembly using bowtie2 (RRID:SCR_005476) [26], we found 93.49 million unique mapped paired-end reads and 62.89 million valid interaction pairs, which were respectively accounted for 26.18% and 17.61% in the clean data (Supplementary Table S5). We employed BWA and Lachesis software to align paired end reads and retain the reads aligned to 500 bp away from each restriction site [27]. According to the conduct of clustering, ordering and orienting to the assembly contigs, these sequences were divided into 8 chromosome clusters and scaffolded by using Lachesis software with tuned parameters (Supplementary Table S6, Fig. 2). Finally, heatmap of Hi-C interaction for finally assembly was shown by R program (Version 3.5.3) [28, 29].

Total 1,862 contigs were used for scaffolding by Hi-C data, which consequently generated 805 scaffolds. The Hi-C assisted chromosome-length scaffolds resulted in a final size of 716.6 Mb accounting for the 99.85% draft genome, which showed a high level of continuity with a contig N50 of 1.1 Mb and a scaffold N50 of 88.78 Mb. The final draft genome assembly of *A.sinensis* was 726.5 Mb (Supplementary Table S3). The anchor rate of contigs (>100 kb) to pseudochromosomes was attained up to the 98.63% based on the Hi-C assembly (Table 1). The scaffold N50 of *A.sinensis* genome was also superior to other published medicinal plant genome assemblies (Supplementary Table S4).

RNA preparation and sequencing

Iso-seq was performed for genome assembly and annotation. The sample mixed with root, stem and leaf using for RNA extraction was obtained from the same plant for DNA sequencing with Oxford Nanopore and frozen in liquid nitrogen immediately. Total RNA was extracted from the frozen tissue using Qiagen RNA extraction kit and then the sequencing library was prepared with SMRTbell™ template prep kit 1.0 (Pacific Biosciences, Menlo Park, CA, USA) after RNA reverse transcription with SMARTer™ PCR cDNA Synthesis kit and cDNA amplification with KAPA HiFi PCR kits. Subsequently, full-length transcriptome sequencing was performed using the PacBio Sequel System.

Total 18,411,342 subreads were obtained from Iso-seq after raw data filtering with SMRTLING 5.1 and then derived 136,050 consensus sequences, of which 94.70% (128, 854) can be aligned to the final genome of *A.sinensis* (Supplementary Table S7).

Genome quality evaluation

To evaluate the completeness of our assembly, we subjected the final assembled genome sequences to Benchmarking Universal Single-Copy Orthologs (BUSCO) version 3 (BUSCO. *Embryophyta* odb 10, RRID:SCR_015008) (BUSCO, *Embryophyta* odb 10) [30,31]. Overall, 95.27% of 1375 expected embryophyta genes were identified in our genome assembly as the complete and partial BUSCO profiles. Among these identified 1310 complete expected embryophyta genes, 1202 and 108 were identified as single copy and duplicated copies respectively (Supplementary Table S8).

Repeat sequences within the *A.sinensis* genome assembly

Transposable elements (TEs) and tandem repeats were identified with both homology-based annotation and *de novo* methods. Consensus sequences of repetitive elements were *de novo* identified and classified using the software package RepeatModeler version 1.04 (RepeatModeler, RRID:SCR 015027) [32]. RepeatMask version 3.2.9 (RepeatMasker, RRID:SCR 012954) [32], RepeatProteinMasker [33] and TRF [34] were used to discover and identify repeats within the respective genomes. Furthermore, simple sequence repeat (SSR) in the *A.sinensis* genome were also classified with MISA (MISA, RRID:SCR 010765) [35].The results showed that *de novo* predicted repeats were more recently active than Repbase [36] predicted repeats (Supplementary Fig. S3). The identified repeat sequences in the *A.sinensis* genome assembly accounted for 59.13% and total length of those accounted for 425.87 Mb (Supplementary Table S9). In particular, the details showed that long terminal repeat (LTR) was the most abundant repeat type and that two non-LTR retrotransposons, short interspersed nuclear element (SINE) and long interspersed nuclear element (LINE) [37], had the lowest proportions in the final assemblies. In addition, 13.12% of repeat sequences could not be classified (Table 2). Total 367,251 SSRs are identified from the draft assembly in 675 scaffolds. Mononucleotides (64.71%), dinucleotides (18.19%), and trinucleotides (12.46%) comprised nearly 96% of SSRs in our assembly (Supplementary Table S10).

Gene prediction and annotation

Three strategies were used for gene prediction. Augustus version 3.2.3 (Augustus, RRID:SCR 008417) [38], GlimmHmm [39] and GeneID (GeneID, RRID:SCR 002473) [40] were used for *ab*

initio gene prediction, using model training based on CDS from *Corchorus olitorius* (COLO4_1.0) [41], *Durio zibethinus* (Duzib1.0) [42], *Gossypium hirsutum* (ASM98774v1) [43], *Herrania umbratica* (ASM216827v2) [44], *Theobroma cacao* (Cirollo_cocoa_geneoe_v2) [45] and *Arabidopsis thaliana* (TAIR10) [46]. GeneWise (GeneWise, RRID:SCR 015054) [47] and GeMoMa [48] were used for homology prediction. PASA (PASA, RRID:SCR 014656) [49] and Tophat (TopHat, RRID:SCR 013035) [50] were used for gene structural prediction based on EST and cDNA sequences. Finally, the total gene prediction was obtained from the union of these three strategies with EVM [49]and filtering the transposable elements with Transposon PSI (Transposon, RRID:SCR 001159) [51]. RNA-seq data of mixed tissues was mapped with the annotation of reference genome using MatchAnnot [52], respectively.

The final annotation was composed of 29,203 genes models with an average of 3,177.62 bp transcripts and 1,114.16 bp coding sequence, and each gene contains 5.02 exon with the average length of 222.09 bp. The comparative information of genes from *A.sinensis* and six closely related plants was also calculated (Supplementary Table S11), including their distributions of CDS and gene length, exon and intron length, exon and intron number (Supplementary Fig. S4). Genes were characterized for their putative function by performing the Blastall [53] and KAAS [54] search of the peptide sequences against the Swiss-Prot (Swiss-Prot, RRID:SCR_002380) [55], NR [56], TrEMBL (TrEMBL, RRID:SCR_002380) [55], KEGG (the Kyoto Encyclopedia of Genes and Genomes, Orthology) database (KEGG, RRID:SCR_012773) [57], COG (Clusters of Orthologous Groups) database (COG, RRID:SCR_007273) [58] and the Gene Ontology (GO) database (GO, RRID:SCR_002811) [59]. Protein conservative models and motifs prediction were used InterProScan version 5.2 (InterproScan, RRID:SCR_005829) [60]. Of these 29,203 protein-coding genes, 82.64% have functional annotation. Summary of hits of database research was as the following: Swiss-Prot (19,586; 67.07%), NR (24,097; 82.52%), TrEMBL(23,455; 80.32%), KEGG (8,494; 29.09%), COG (13,592; 46.54%), GO (14,019, 78.70%) and InterProScan (20,031; 68.59%) (Supplementary Table S12). In addition, we also identified 207 miRNAs, 34 rRNAs, 173 tRNAs and 1,173 snRNAs via Rfam non-coding RNA (ncRNA) database (Rfam, RRID:SCR 007891) [61], tRNAscan-SE (tRNAscan-SE, RRID:SCR 010835) [62] and RNAmmer [63]. The average length, total length and percentage of ncRNAs in *A.sinensis* genome were further assessed (Supplementary Table S13). In addition, 48.61% of predicted genes (14,197) were supported by Iso-seq transcripts

(Supplementary Table S14).

Gene family identification and phylogenetic tree construction

By keeping the longest transcript for each gene, whole protein coding genes sets from *A.sinensis* genome and other 12 representative plant genomes including *G.hirutum* (ASM98774v1), *A.thaliana* (TAIR10), *T.cacao* (Cirollo_cocoa_geneoe_v2), *Cephalotus follicularis* (Cfol_1.0), *Citrus clementina* (Citrus_clementina_v1.0), *Cucurbita pepo* (ASM280686v2), *Eucalyptus grandis* (Egrandis1_0), *Glycine max* (Glycine_max_v2.1), *Helianthus annuus* (HanXRQr 1.0), *Populus euphratica* (PopEup_1.0), *Quercus suber* (CorkOak 1.0),and *Vitis vinifera* (assembly 12X) were used to construct a global gene family classification with all-vs-all BLASTP($1e^{-5}$ cutoff, Blast+ v2.3.056) and OrthoMCL version 2.0.9 (Ortholog Groups of Protein Sequences, RRID: SCR_007839) [64]. The default settings were used for BLASTP and OrthoMCL. In our assembly, 21,955 genes were clustered into 13,713 gene families. Gene family analysis also revealed that 789 gene families and 7,248 genes were unique to *A.sinensis* in above comparation (Fig.3a and Supplementary Table S15). Of these, 9,615 gene families were shared among *A.sinensis* and four representative species (*G.hirsutum* from Malvaceae, *C.olitorius* from Tiliaceae, *T.cacao* from Sterculiaceae and *A.thaliana* as the model plant from Cruciferae), whereas 804 gene families were unique to *A.sinensis* genome (Fig. 3b). Malvaceae, Tiliaceae and Sterculiaceae are beyond the order Malvales and the Thymelaeceae family is also divided into order Malvales in APG IV [65].

Single-copy genes or the orphan genes with only single copy in genome during duplication and evolution of species are highly conserved, which are generally used for establishing genetic relationship and origin of species. Alignment of single-copy gene was performed with protein sequences by Mafft (Mafft, RRID: SCR_011811) [66], then poorly aligned and highly divergent sites were filtering with the Gblocks (Gblocks, RRID: SCR_015945) [67] and the final CDSs were used for evolutional analyses by RaxML with GTRGAMMA model (RaxML, RRID: SCR_006086) [68]. The bootstrap was 100 and *Helianthus annuus* from the Asterids was the outgroup [69]. We constructed a phylogenetic tree and estimated the divergence time of 13 plants by 89 single-copy gene families with the MCMCTREE of PAML [70] (Supplementary Fig. S5) (Parameters: clock = 2, RootAge = <100.6, model = 7, BDparas = 1 1 0 , kappa gamma = 6 2 , alpha gamma = 1 1, rgene gamma = 2 3.18, sigma2 gamma = 1 1.3; Divergence time of Asterids and Rosids (~118 Mya) was

used for calibration [69]). The divergence time between *A.sinensis* and *A.thaliana* was estimated as 82.14 (67.63-93.99) million years ago (Mya), and the divergence time between *A.sinensis* and the common ancestor of *G.hisutum* and *T.cacao* from Malvales order was approximately 69.64 (53.18-84.37) million years ago (Fig. 3c and Supplementary Fig. S6), whereas the divergence time between *G.hisutum* and *T.cacao* have been determined as 31.33-69.23 million years ago in our analysis and this is concordance with the previous studies [71, 72].

Gene family expansion and contraction

Expansion and contraction of defining gene family is an important driver of metabolite variation and species adaptation during plant evolution [73]. We determined the expansion and contraction of orthologous gene families in *A.sinensis* genome by CAFÉ 2.2 (CAFÉ, RRID: SCR_005983) with default parameters[74]. We inferred 53 expanded families and 117 contracted families with *A.sinensis* genome after comparing 11,855 gene families across all 13 species (Fig.3c and Supplementary Table S16). Using Blast2GO (B2G, RRID: SCR_005828) to enrich the ontology categories (GO and KEGG terms), The expanded gene families involved in the pathways of plant circadian rhythm, tricarboxylic acid cycle, propanoate metabolism, Ribosome biogenesis and aminoacyl-tRNA biosynthesis (Supplementary Table S17 and Fig. S7), and the contracted gene families mapped pathways of starch/sucrose metabolism, sesquiterpenoid and triterpenoid biosynthesis and linoleic acid metabolism (Supplementary Table S18 and Fig. S8).

4DTv Distribution

We used MCScanX to identify the syntenic regions [75]. The longest isoform for each gene was selected for this exercise. The best five mutual hit of the BLASTP results in gene family analysis were used as input. Only the syntenic segments that have more than five gene pairs were considered for four-fold degenerate synonymous sites (4DTv) calculation. Pairwise sequence was aligned using MUSCLE [76]. Raw 4DTv values were corrected for possible multiple transversions at the same site. Based on 4DTv distribution, a large accumulation of gene duplications is evident in the *A.sinensis* genome and distinct from the scenarios in *A.thaliana*, *M.truncatula* and *V.vinifera* (Fig. 3d).

Conclusions

In sum, a high-quality *de novo* genome assembly and in-depth characterization for *A.sinensis*, combining with Nanopore single-molecule long reads and Hi-C has been provided in this study. The

final assembly is approximately 726.5 Mb, which was slightly smaller than the k-mer estimated genome size of 773.3 Mb. The Hi-C data was used to revise the mis-assemblies and assign the contigs into a chromosome-scale scaffolds, and consequently generated an assembly with a high level of continuity with a contig N50 of 1.1 Mb and a scaffold N50 of 88.78 Mb. We also predicted 29,203 protein-coding genes from the final assembly and 82.64% (24,133 genes) of all protein-coding genes were annotated. We estimated that the divergence time between *A.sinensis* and its common ancestor with *G.hisutum* and *T.cacao* from Malvales order was approximately 53.18-84.37 million years ago. The genome of *A.sinensis* seems to experience a recent whole-genome duplication event after the K-T boundary [77]. The chromosome-level genome assembly of *A.sinensis* is also the first high-quality genome in the Thymelaeceae family. Considering the vulnerable and severely endangered status of natural *A.sinensis* tree populations due to stem heavily exploited for creating costly agarwood products, the genome assembly of *A.sinensis* tree presented here will provide valuable information to aid the global conservation of these precious species and contribute to understanding the mechanism of the agarwood formation, eventually will help us reveal the evolution of aromatic genes and plants.

Availability of supporting data

Supporting data and materials are available in the *GigaScience* GigaDB database (GigaDB, RRID:SCR 004002) [78], with the raw genomics sequences deposited in the NCBI Sequence Read Archive (SRA) database under the BioProject accession number PRJNA556948 and BioSample accession number SAMN12385133.

Additional files

S Fig.1 K-mer (k = 19) analysis for estimating the size of the *Aquilaria sinensis* genome.

S Fig.2 GC content and average sequencing depth of the Illumina sequencing data used for genome estimation.

S Fig.3 Distribution of sequence divergence rates of different TE types with Repbase (A) and *de novo* (B) methods in *Aquilaria sinensis* genome.

S Fig.4 Distribution of gene elements in *Aquilaria sinensis* genome and other six plant genome.

S Fig.5 Phylogenetic tree of 13 plant species including *Aquilaria sinensis.*

S Fig.6 Estimation of divergence time of 13 plant species investigated in the present study. The colored numbers on the nodes are the divergence time from present (million years ago). Numbers

in the bracket indicate the 95% confidence interval of the divergence time.

S Fig.7 GO enrichment of expansion gene families in *Aquilaria sinensis* genome.

S Fig.8 GO enrichment of contraction gene families in *Aquilaria sinensis* genome.

S Table 1 Summary of Nanopore sequencing for *Aquilaria sinensis* genome.

S Table 2 Supporting of Illumina data for Nanopore data in *Aquilaria sinensis* genome assembled.

S Table 3 Statistics of the results of *Aquilaria sinensis* genome assembly before Hi-C mapping.

S Table 4 Comparisons of genome assemblies of medicinal plants based on descending Contig N50.

S Table 5 Summary of mapping status of Hi-C data.

S Table 6 Statistics of pseudochromosomes length and scaffold number.

S Table 7. Mapping result of Iso-seq from *Aquilaria sinensis*.

S Table 8 Statistics of BUSCO evolution for *Aquilaria sinensis* genome.

S Table 9 Statistics of repeat sequence in *Aquilaria sinensis* genome via different methods.

S Table 10 Statistics of SSRs in *Aquilaria sinensis* genome sequences.

S Table 11 Statistics of characters of gene models in *Aquilaria sinensis* and other six plant genome.

S Table 12 The annotated genes of *Aquilaria sinensis* which can be functionally classified in each corresponding database.

S Table 13 Noncoding RNA annotation in the *Aquilaria sinensis* genome.

S Table 14. Annotation of Iso-seq and compared with genome annotation of *Aquilaria sinensis* genome.

S Table 15 Summary of gene families among 13 plant species.

S Table 16 Summary of gene families changes among 13 species.

S Table 17 KEGG mapping of expansion gene families in *Aquilaria sinensis* genome.

S Table 18 KEGG mapping of contraction gene families in *Aquilaria sinensis* genome.

Abbreviation

IUCN: International Union for Conservation of Nature and Natural resources; SMRT: single molecular real time; Iso-seq: Isoform sequencing; BUSCO: Benchmarking Universal Single-Copy Orthologs; Hi-C: high-throughput chromosome conformation capture; TE: transposable element;

EVM: EVidenceModeler;NR: PASA: Program to Assemble Spliced Alignments; Nr: NCBI non-redundant protein database; PCR: polymerase chain reaction; Pfam: protein families; TrEMBL: Translated EMBL-Bank; MYA: million years ago. CAFÉ: Computational Analysis of gene Family Evolution; MRCA: most recent common ancestor; K-T: Cretaceous-Tertiary.

Competing interests

The authors declare that they have no competing interests.

Funding

This work was supported by the Central Public-interest Scientific Institution Basal Research Fund for Chinese Academy of Tropical Agricultural Sciences (17CXTD-15), the National Natural Science Foundation of China (31870668) and the China Agriculture Research System (CARS-21). We are grateful to NextOmics Co., Ltd. (Wuhan, China) for providing technical help.

Author contributions

H.F.D., P.C. and W.L.M. conceptualized the research program. X.P.D., W.L.M. and S.Q.P. designed experiments and coordinated the program. S.Z.H. collected the sample and J.W. take the photos. H.L.L and J.H.Z. extracted the DNA. X.P.D., Q.L., H.W., P.C., W.L., H.Q.C., W.H.D., D.G. and C.H.C were partially involved either experiments or data analysis. X.P.D. and Q.L. wrote the manuscript. All authors read and approved the final manuscript.

References

1. Kumeta Y and Ito M. Characterization of α-humulene synthases responsible for the production of sesquiterpenes induced by methyl jasmonate in *Aquilaria* cell culture. Journal of Natural Medicines 2016;70(3): 452-459.

2. Xu Y, Zhang Z, Wang M, et al. Identification of genes related to agarwood formation: transcriptome analysis of healthy and wounded tissues of *Aquilaria sinensis*. BMC Genomics 2013;14(1): 227.

3. Naef R. The volatile and semi- volatile constituents of agarwood, the infected heartwood of *Aquilaria* species: a review. Flavour and Fragrance Journal 2011;26(2): 73-87.

4. Liao G, Mei WL, Kong FD, et al. 5, 6, 7, 8-Tetrahydro-2-(2-phenylethyl) chromones from artificial agarwood of *Aquilaria sinensis* and their inhibitory activity against acetylcholinesterase. Phytochemistry 2017;139: 98-108.

5. Hashim Y Z H Y, Kerr P G, Abbas P, et al. *Aquilaria* spp.(agarwood) as source of health beneficial compounds: A review of traditional use, phytochemistry and pharmacology. Journal of Ethnopharmacology 2016, 189: 331-360.

6. Ma CT, Eom T, Cho E, et al. Aquilanols A and B, macrocyclic humulene-type sesquiterpenoids from the agarwood

of *Aquilaria malaccensis*. Journal of Natural Products 2017, 80(11): 3043-3048.

7. Yang L, Yang YL, Dong WH, et al. Sesquiterpenoids and 2-(2-phenylethyl) chromones respectively acting as α-glucosidase and tyrosinase inhibitors from agarwood of an *Aquilaria* plant. Journal of Enzyme Inhibition and Medicinal Chemistry 2019;34(1): 853-862.

8. Liao G, Dong W H, Yang J L, et al. Monitoring the chemical profile in agarwood formation within one year and speculating on the biosynthesis of 2-(2-phenylethyl) chromones. Molecules 2018;23(6): 1261.

9. Chhipa H, Chowdhary K, Kaushik N. Artificial production of agarwood oil in *Aquilaria* sp. by fungi: a review. Phytochemistry Reviews 2017;16(5): 835-860.

10. Azren P D, Lee S Y, Emang D, et al. History and perspectives of induction technology for agarwood production from cultivated Aquilaria in Asia: a review. Journal of forestry research 2019, 30(1): 1-11.

11. Harvey-Brown, Y. *Aquilaria sinensis*. The IUCN Red List of Threatened Species 2018. 2018; e.T32382A2817115. http://dx.doi.org/10.2305/IUCN.UK.2018-2.RLTS.T32382A2817115.en

12. Wang Y, Zhan D F, Jia X, et al. Complete chloroplast genome sequence of *Aquilaria sinensis* (Lour.) Gilg and evolution analysis within the Malvales order. Frontiers in Plant Science 2016;7: 280.

13. Wang X, Gao B, Liu X, et al. Salinity stress induces the production of 2-(2-phenylethyl) chromones and regulates novel classes of responsive genes involved in signal transduction in *Aquilaria sinensis* calli. BMC plant biology 2016;16(1): 119.

14. Wang X, Zhang Z, Dong X, et al. Identification and functional characterization of three type III polyketide synthases from *Aquilaria sinensis* calli. Biochemical and biophysical research communications 2017;486(4): 1040-1047.

15. Porebski S, Bailey LG, Baum BR. Modification of a CTAB DNA extraction protocol for plants containing high polysaccharide and polyphenol components. Plant Molecular Biology Reporter 1997;15(1): 8-15.

16. Liu B, Shi Y, Yuan J, et al. Estimation of genomic characteristics by analyzing k-mer frequency in de novo genome projects. arXiv preprint arXiv 2013: 1308.2012. https://arxiv.org/abs/1308.2012.

17. Ding X, Mei W, Huang S, et al. Genome survey sequencing for the characterization of genetic background of *Dracaena cambodiana* and its defense response during dragon's blood formation. PloS ONE 2018;13(12): e0209258.

18. Vurture G W, Sedlazeck F J, Nattestad M, et al. GenomeScope: fast reference-free genome profiling from short reads. Bioinformatics 2017; 33(14): 2202-2204.

19. Leggett RM and Clark MD. A world of opportunities with nanopore sequencing. Journal of Experimental Botany

2017;68(20): 5419-5429.
20. Schmidt MHW, Vogel A, Denton AK, et al. *De novo* assembly of a new *Solanum pennellii* accession using nanopore sequencing. The Plant Cell 2017;29(10): 2336-2348.
21. Walker B J, Abeel T, Shea T, et al. Pilon: an integrated tool for comprehensive microbial variant detection and genome assembly improvement. PloS ONE 2014;9(11): e112963.
22. Li H, Durbin R. Fast and accurate long-read alignment with Burrows-Wheeler transform. Bioinformatics 2010; 26(5): 589-595.
23. Lieberman-Aiden E, Van Berkum NL, Williams L, et al. Comprehensive mapping of long-range interactions reveals folding principles of the human genome. Science 2009;326(5950): 289-293.
24. Xu CQ, Liu H, Zhou SS, et al. Genome sequence of *Malania oleifera*, a tree with great value for nervonic acid production. GigaScience 2019;8(2): giy164.
25. Chen S, Zhou Y, Chen Y, et al. fastp: an ultra-fast all-in-one FASTQ preprocessor. Bioinformatics 2018;34(17): i884-i890.
26. Langmead B, Salzberg SL. Fast gapped-read alignment with Bowtie 2. Nature Methods 2012;9(4): 357.
27. Burton J N, Adey A, Patwardhan R P, et al. Chromosome-scale scaffolding of de novo genome assemblies based on chromatin interactions. Nature Biotechnology 2013;31(12): 1119.
28. R Core Team. R: A language and environment for statistical computing. 2019. https://www.R-project.org/.
29. Yin D, Ji C, Ma X, et al. Genome of an allotetraploid wild peanut *Arachis monticola*: a de novo assembly. GigaScience, 2018;7(6): giy066.
30. Simão FA, Waterhouse RM, Ioannidis P, et al. BUSCO: assessing genome assembly and annotation completeness with single-copy orthologs. Bioinformatics 2015;31(19): 3210-3212.
31. Waterhouse RM, Seppey M, Simão FA, et al. BUSCO applications from quality assessments to gene prediction and phylogenomics. Molecular Biology and Evolution 2017;35(3): 543-548.
32. Bedell JA, Korf I, Gish W. MaskerAid: a performance enhancement to RepeatMasker. Bioinformatics 2000;16(11): 1040-1041.
33. Allred DB, Cheng A, Sarikaya M, et al. Three-dimensional architecture of inorganic nanoarrays electrodeposited through a surface-layer protein mask. Nano Letters 2008;8(5): 1434-1438.
34. Benson G. Tandem repeats finder: a program to analyze DNA sequences. Nucleic Acids Research 1999;27(2): 573-580.
35. Thiel T, Michalek W, Varshney R, et al. Exploiting EST databases for the development and characterization of

gene-derived SSR-markers in barley (*Hordeum vulgare* L.). Theoretical and Applied Genetics 2003;106(3): 411-422.

36. Jurka J, Kapitonov V V, Pavlicek A, et al. Repbase Update, a database of eukaryotic repetitive elements[J]. Cytogenetic and Genome Research 2005;110(1-4): 462-467.

37. Yang L, Scott LA, Wichman HA. Tracing the history of LINE and SINE extinction in sigmodontine rodents. Mobile DNA 2019;10(1): 22.

38. Stanke M, Steinkamp R, Waack S, et al. AUGUSTUS: a web server for gene finding in eukaryotes. Nucleic Acids Research 2004;32(suppl_2): W309-W312.

39. Majoros WH, Pertea M, Salzberg SL. TigrScan and GlimmerHMM: two open source ab initio eukaryotic gene-finders. Bioinformatics 2004;20(16): 2878-2879.

40. Blanco E, Parra G, Guigó R. Using geneid to identify genes. Current Protocols in Bioinformatics 2007;18(1): 4.3. 1-4.3. 28.

41. Islam MS, Saito JA, Emdad EM, et al. Comparative genomics of two jute species and insight into fibre biogenesis. Nature Plants 2017;3(2): 16223.

42. Teh BT, Lim K, Yong CH, et al. The draft genome of tropical fruit durian (*Durio zibethinus*). Nature Genetics 2017;49(11): 1633.

43. Li F, Fan G, Lu C, et al. Genome sequence of cultivated Upland cotton (*Gossypium hirsutum* TM-1) provides insights into genome evolution. Nature Biotechnology 2015;33(5): 524.

44. *Herrania umbratica*. https://www.ncbi.nlm.nih.gov/genome/55117

45. Argout X, Martin G, Droc G, et al. The cacao Criollo genome v2. 0: an improved version of the genome for genetic and functional genomic studies. BMC Genomics 2017;18(1): 730.

46. Michael TP, Jupe F, Bemm F, et al. High contiguity *Arabidopsis thaliana* genome assembly with a single nanopore flow cell. Nature Communications 2018;9(1): 541.

47. Birney E, Durbin R. Using GeneWise in the Drosophila annotation experiment. Genome Research 2000;10(4): 547-548.

48. Keilwagen J, Hartung F, Grau J. GeMoMa: Homology-Based Gene Prediction Utilizing Intron Position Conservation and RNA-seq Data. Gene Prediction. Humana, New York, NY, 2019: 161-177.

49. Haas BJ, Salzberg SL, Zhu W, et al. Automated eukaryotic gene structure annotation using EVidenceModeler and the Program to Assemble Spliced Alignments. Genome Biology 2008;9(1): R7.

50. Trapnell C, Pachter L, Salzberg SL. TopHat: discovering splice junctions with RNA-Seq. Bioinformatics

2009;25(9): 1105-1111.

51. Yagi M, Kosugi S, Hirakawa H, et al. Sequence analysis of the genome of carnation (*Dianthus caryophyllus* L.). DNA Research 2013;21(3): 231-241.

52. Hu J, Uapinyoying P, Goecks J. Interactive analysis of Long-read RNA isoforms with Iso-Seq Browser. BioRxiv 2017: 102905.

53. Coordinators NR. Database resources of the national center for biotechnology information. Nucleic Acids Research 2017;45: D12.

54. Moriya Y, Itoh M, Okuda S, et al. KAAS: an automatic genome annotation and pathway reconstruction server[J]. Nucleic Acids Research 2007;35(suppl_2): W182-W185.

55. Boeckmann B, Bairoch A, Apweiler R, et al. The SWISS-PROT protein knowledgebase and its supplement TrEMBL in 2003. Nucleic Acids Research 2003;31(1): 365-370.

56. Yu K, Zhang T. Construction of customized sub-databases from NCBI-nr database for rapid annotation of huge metagenomic datasets using a combined BLAST and MEGAN approach. PLoS ONE 2013;8(4): e59831.

57. Kanehisa M, Furumichi M, Tanabe M, et al. KEGG: new perspectives on genomes, pathways, diseases and drugs. Nucleic Acids Research 2016;45(D1): D353-D361.

58. Kristensen DM, Kannan L, Coleman MK, et al. A low-polynomial algorithm for assembling clusters of orthologous groups from intergenomic symmetric best matches. Bioinformatics 2010;26(12): 1481-1487.

59. Gene Ontology Consortium. Gene ontology consortium: going forward. Nucleic Acids Research 2014;43(D1): D1049-D1056.

60. Hunter S, Apweiler R, Attwood TK, et al. InterPro: the integrative protein signature database. Nucleic Acids Research 2008;37(suppl_1): D211-D215.

61. Griffiths-Jones S, Moxon S, Marshall M, et al. Rfam: annotating non-coding RNAs in complete genomes[J]. Nucleic Acids Research 2005;33(suppl_1): D121-D124.

62. Lowe TM, Eddy SR. tRNAscan-SE: a program for improved detection of transfer RNA genes in genomic sequence. Nucleic Acids Research 1997;25(5): 955-964.

63. Lagesen K, Hallin P, Rødland E A, et al. RNAmmer: consistent and rapid annotation of ribosomal RNA genes. Nucleic Acids Research 2007;35(9): 3100-3108.

64. Li L, Stoeckert CJ, Roos DS. OrthoMCL: identification of ortholog groups for eukaryotic genomes. Genome Research 2003;13(9): 2178-2189.

65. Chase MW, Christenhusz MJM, Fay MF, et al. An update of the Angiosperm Phylogeny Group classification for

the orders and families of flowering plants: APG IV. Botanical Journal of the Linnean Society 2016;181(1): 1-20.

66. Katoh K, Standley D M. MAFFT multiple sequence alignment software version 7: improvements in performance and usability. Molecular Biology and Evolution 2013;30(4): 772-780.

67. Castresana, J. Selection of Conserved Blocks from Multiple Alignments for Their Use in Phylogenetic Analysis. Molecular Biology and Evolution 2000 17(4):540-552.

68. Stamatakis, A. RAxML-VI-HPC: maximum likelihood-based phylogenetic analyses with thousands of taxa and mixed models. Bioinformatics 2006;22(21):2688-2690.

69. Badouin H, Gouzy J, Grassa CJ, et al. The sunflower genome provides insights into oil metabolism, flowering and Asterid evolution. Nature 2017;546(7656): 148.

70. Yang Z. PAML 4: phylogenetic analysis by maximum likelihood. Molecular Biology and Evolution 2007;24(8): 1586-1591.

71. Li F, Fan G, Lu C, et al. Genome sequence of cultivated Upland cotton (*Gossypium hirsutum* TM-1) provides insights into genome evolution. Nature biotechnology 2015; 33(5): 524.

72. Teh B T, Lim K, Yong C H, et al. The draft genome of tropical fruit durian (*Durio zibethinus*). Nature genetics 2017; 49(11): 1633.

73. Denoeud F, Carretero-Paulet L, Dereeper A, et al. The coffee genome provides insight into the convergent evolution of caffeine biosynthesis. Science 2014;345(6201): 1181-1184.

74. De Bie T, Cristianini N, Demuth J P, et al. CAFE: a computational tool for the study of gene family evolution. Bioinformatics 2006;22(10): 1269-1271.

75. Wang Y, Tang H, DeBarry J D, et al. MCScanX: a toolkit for detection and evolutionary analysis of gene synteny and collinearity. Nucleic Acids Research 2012;40(7): e49-e49.

76. Edgar R C. MUSCLE: multiple sequence alignment with high accuracy and high throughput. Nucleic Acids Research 2004;32(5): 1792-1797.

77. Fawcett J A, Maere S, Van De Peer Y. Plants with double genomes might have had a better chance to survive the Cretaceous–Tertiary extinction event. Proceedings of the National Academy of Sciences 2009;106(14): 5737-5742.

78. Ding X, Mei W, Lin Q et al. Supporting data for “Genome sequence of agarwood tree *Aquilaria sinensis* (Lour.) Spreng: the first chromosome-level draft genome in the Thymelaeceae family” GigaScience Database 2019.

Figure legends

Figure 1: Morphological characteristic of *Aquilaria sinensis*. (a) mature tree; (b) flower; (c) fruit; (d) seed; (e) cracked seed; (f) agarwood generation; (g) agarwood. The pictures of numbered b, c, d and e were taken with stereoscopic fluorescence microscope (Olympus SZX16, Pittsburgh, PA) under the dark field. All the photos were taken by Dr. Jun Wang and processed by Dr. Xupo Ding.

Figure 2: Hi-C interaction matrix for *A.sinensis* genome assembly with 8 clusters.

Figure 3: Comparative genomic analysis of *Aquilaria sinensis* and other plant species. (a) Distribution of genes and gene families of 13 plant species we investigated. (b) A Venn diagram showing the distribution of shared gene families among the Malvales plants *Aquilaria sinensis* (agarwood), *Theobroma cacao* (cocoa), *Gossypium hirsutum* (cotton), *Corchorus olitorius* (jute) and the model plant *Arabidopsis thaliana* (Arabidopsis). (c) Divergence time estimation and gene families changes among 13 plant species. The black number at each node denotes estimated divergence time from present (million years ago). The blue number at the root (11885) denotes the total number of gene families predicted in the most recent common ancestor (MRCA) and the green/red numbers around each branch denote gene family gain/loss number. The red nodes indicate the known divergence time of Asterids and Rosids. (d) 4dTv distribution in selected assemblies of *A.sinensis*, *A.thaliana*, *O.sativa*, *M.truncatula* and *V.vinifera*. 4dTv, transversion substitutions at four-fold degenerate sites.

Table legends

Table 1. Statistics of the final genome assembly for *Aquilaria sinensis.*

Table 2. Statistics of transposable elements in *Aquilaria sinensis* genome sequences.

Table 1. Statistics of the final genome assembly for *Aquilaria sinensis.*

Statistics Type	Contig Length (bp)	Contig Number	Scaffold Length (bp)	Scaffold Number
N50	1,058,652	164	88,784,932	4
N60	726,407	246	86,380,100	5
N70	495,861	366	84,956,755	6
Longest	11,913,571	1	109,870,270	1
Total	720,187,708	2,015	726,587,161	9
Length>=1kb	720,187,482	2,013	726,587,161	9
Length>=2kb	720,179,880	2,008	726,587,161	9
Length>=5kb	720,112,854	1,991	726,587,161	9

Table 2. Statistics of transposable elements in Aquilaria sinensis genome sequences. Click here to access/download;Table;Table 2.docx

Table 2. Statistics of transposable elements in *Aquilaria sinensis* genome sequences.

Type	Repbase TEs		Mips-REdat TEs		TE proteins		RepeatModeler		Combined TEs	
	Length (Mb)	% in genome	Length (Mb)	%in genome	Length (Mb)	% in Genome	Length (Mb)	% in genome	Length (Mb)	% in genome
DNA	13223408	1.84	1392136	0.19	10456270	1.45	28698131	3.98	38895471	5.4
LINE	2916904	0.41	253492	0.04	7680548	1.07	6394899	0.89	12239695	1.7
LTR	73748923	10.24	22973865	3.19	75336839	10.46	138348032	19.21	192609862	26.74
SINE	2232	0	1145	0	0	0	0	0	4539	0
Other	6189190	0.86	380555	0.05	1369337	0.19	0	0	87659087	12.17
Unknown	35443	0	0	0	0	0	124331790	17.26	94460416	13.12
Total	96116100	13.35	25001193	3.47	94842994	13.17	296679047	41.19	425869070	59.13

Figure 1. Morphological characteristic of Aquilaria sinensis.

Click here to access/download;Figure;Figure 1.tif

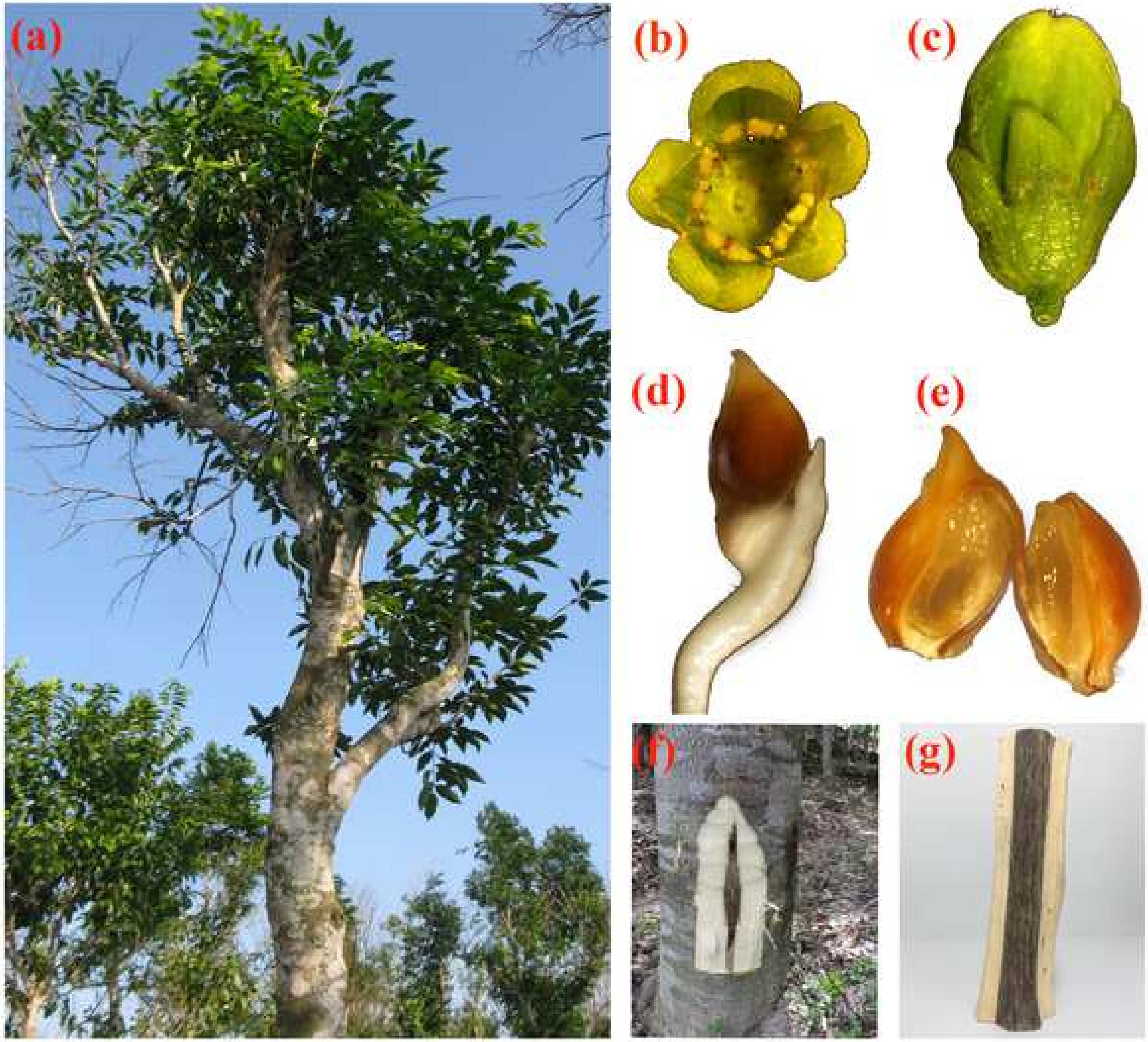

Figure 2. Hi-C interaction matrix for A.sinensis genome assembly with 8 clusters.

Click here to access/download;Figure;Figure 2.tif

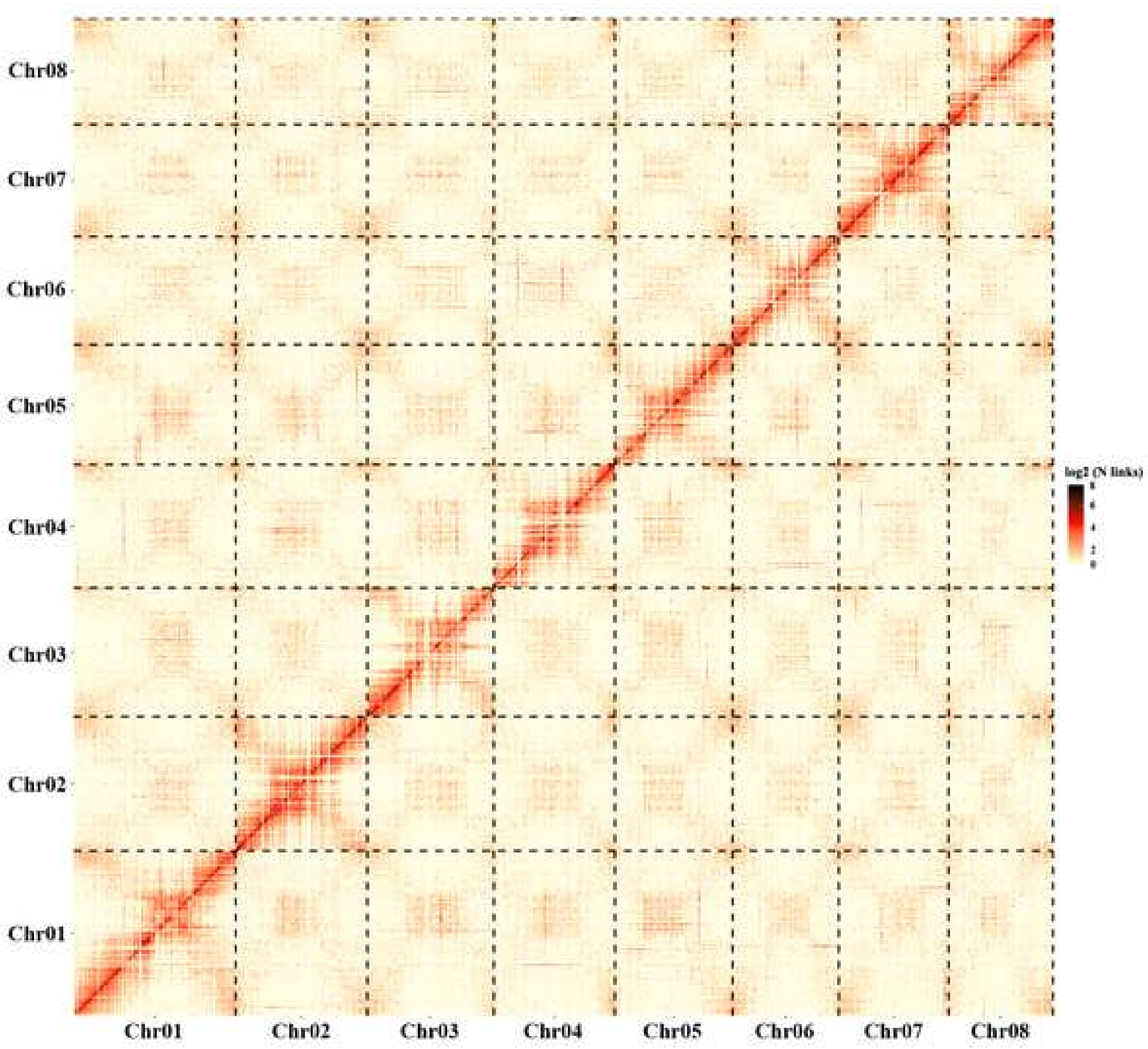

Figure 3. Comparative genomic analysis of Aquilaria sinensis and other plant species.

Click here to access/download;Figure;Figure.3.tif

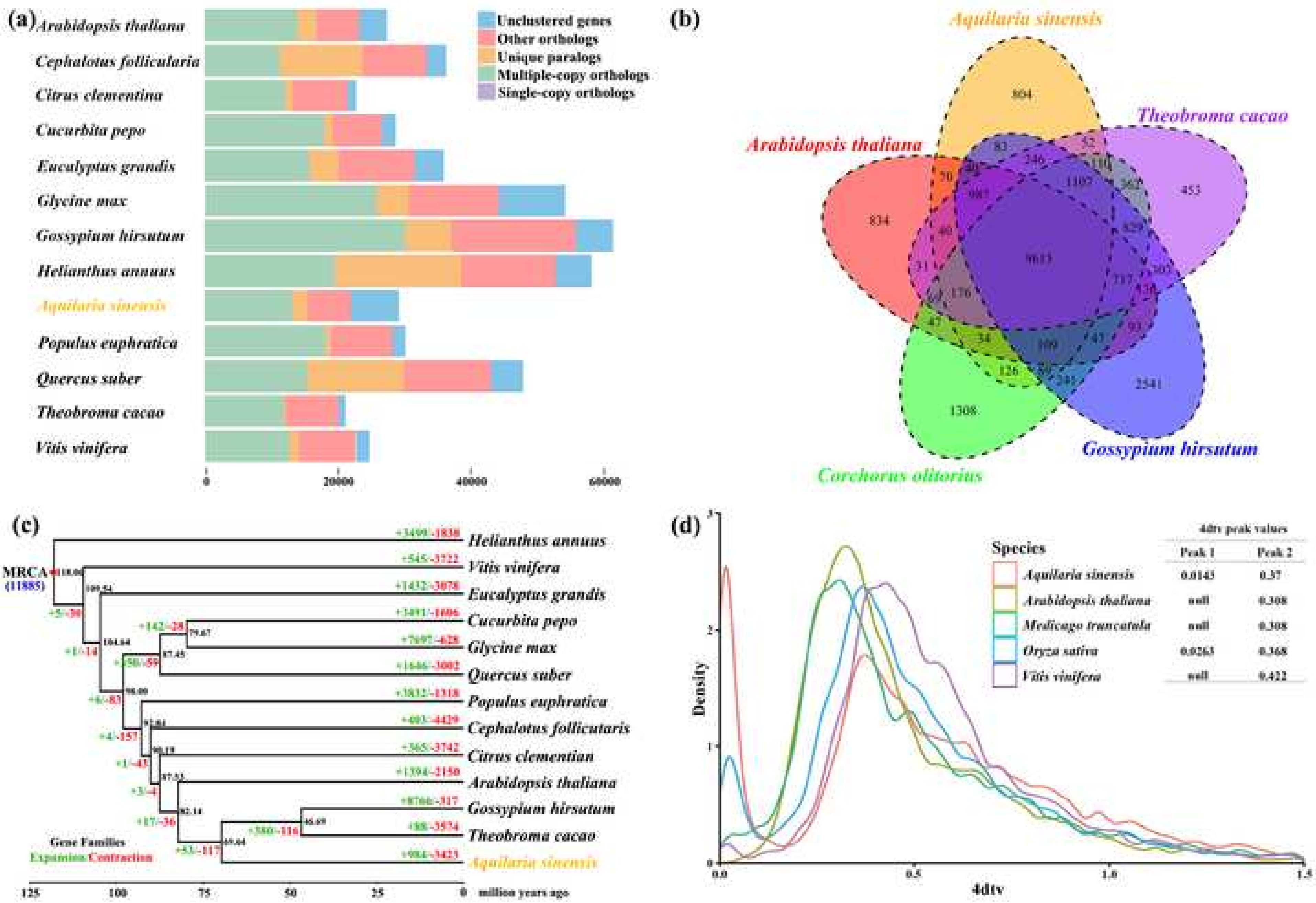

Click here to access/download
Supplementary Material
S Table1 - S Table 18.xls

S Fig.1 K-mer (k = 19) analysis for estimating the size of the Aquilaria sinensis genome.

Click here to access/download
Supplementary Material
S Fig.1.tif

S Fig.2 GC content and average sequencing depth of the Illumina sequencing data used for genome estimation.

Click here to access/download
Supplementary Material
S Fig.2.tif

S Fig.3 Distribution of sequence divergence rates of different TE types with Repbase (A) and de nove (B) methods in Aquilaria

Click here to access/download
Supplementary Material
S Fig.3.tif

S Fig.4 Distribution of gene elements in Aquilaria sinensis genome and other six plant genome.

Click here to access/download
Supplementary Material
S Fig.4.tif

S Fig.5 Phylogenetic tree of 13 plant species including Aquilaria sinensis.

Click here to access/download
Supplementary Material
S Fig.5.tif

S Fig.6 Estimation of divergence time of 13 plant species investigated in the present study.

Click here to access/download
Supplementary Material
S Fig.6.tif

S Fig.7 GO enrichment of expansion gene families in Aquilaria sinensis genome.

Click here to access/download

Supplementary Material

S Fig.7.tif

S Fig.8 GO enrichment of contraction gene families in Aquilaria sinensis genome.

Click here to access/download

Supplementary Material

S Fig.8.tif

Click here to access/download
Supplementary Material
Response to Reviewer1.docx

Click here to access/download
Supplementary Material
Response to Reviewer2.docx

Click here to access/download
Supplementary Material
Revised Manuscript with track changes.docx

 Click here to access/download;Personal Cover;Cover letter for revised manuscript.docx

Dear editor and reviewers:

Thanks for your letter and the reviewers' comments concerning our manuscript entitled "Genome sequence of agarwood tree *Aquilaria sinensis* (Lour.) Spreng: the first chromosome-level draft genome in the Thymelaeceae family" (ID: GIGA-S-19-00378). Those comments are all valuable and very helpful for revising and improving our paper, as well as the important guiding the significance to our future research. We have studied the comments carefully and have made correction which we hope meeting with approval. Especially we upload the relevant data to the NCBI-SRA and revised the language. Revised positions are marked in red in the manuscript with track changes. The main corrections in this revised manuscript and the responds to the reviewer comments are as the following:

Response to the Reviewer #1:

Comment: The authors describe the first chromosome-level genome assembly of the agarwood tree. This work is of great interest because of the intensive use of the agar tree and the need of preservation of this tree. The authors have produced a big work to assemble and annotate the genome with good results.

1. Comment: The methods are well described but in line 204, the authors introduce RNA sequencing of mixed tissues. There is no description of the generation of those data (type of tissues used, RNA extraction method, RNA sequencing libraries preparation, sequencing depth ...). The data are not available in the NCBI Sequence Read Archive under the BioProject accession number given.

Response：The description of the RNA-seq data have been added and can be tracked with red marks in the revised manuscript from line 170 to line 179. Raw data have been submitted to PRJNA556948 in the SRA database of NCBI and its run number is SRR10276652.

2. Comment: The table 1 doesn't contain the statistics of the final assembly (same table than Supplementary table S3). What is the proportion of the gaps introduced after scaffolding with Hi-C?

Response：The new table 1 about the final assembly have been upload in the revised manuscript. The proportion of gaps is roughly 0.9% (~length of 6.4 Mb) in the final genome.

3. Comment: In the figure 3d, a table with the values of the picks could be added (minor issue).

Response：The values of the peaks have been added.

4. Comment: In the line 285, the authors used the HI-C data to correct the mis-assemblies. How many mis-assemblies were found? How have they been detected?

Response：In the initial Hi-C interactive heatmap, the regions of the interactive intensity between different pseudochromosomes higher than their intensity within same pseudochromosome were determined as the mis-assemblies. Then these regions were corrected based on the interactive intensities of re-clustered after the contig divided into the equal length of 100 kb. The following table is the comparison of initial Hi-C assembly and the correct Hi-C assembly:

Chr	Initial assembly		Final assembly	
	Size	Scaf Num	Size	Scaf Num
Chr01	114,318,616	151	109,870,270	166
Chr02	108,239,053	49	99,503,772	60
Chr03	94,462,133	70	89,391,083	91
Chr04	89,950,395	72	88,784,932	86
Chr05	85,082,375	76	86,380,100	112
Chr06	80,447,689	69	84,956,755	91
Chr07	76,713,504	87	80,601,762	89
Chr08	76,239,003	94	77,134,862	110
Total	725,452,768	668	716,623,536	805

5. Comment: Finally, the Hi-C sequencing data are not available in the NCBI Sequence Read Archive under the BioProject accession number given.

Response：The raw data of Hi-C sequencing have been submitted to PRJNA556948 in the SRA database of NCBI now and its run number is SRR10362483.

Response to Reviewer #2:

Comment: The genome sequence presented in your manuscript is a highly valuable resource for sustainable commercial exploitation and to help understanding the evolution of aromatic plants. You have reached a high level of continuity, obtainng a well-annotated chromosome-level assembly. In addition, the phylogenetic analysis is consistent with previous divergence time estimates and the analysis of gene families help to define the evolutionary context of the released the genome. However, before accepting the publication, I would like you to do a major revision of the manuscript and address some of my concerns.

1. Comment: In the background you state that *A. sinensis* have been included in the IUCN red list. Although that's true you don't mention the current assessment as VULNERABLE (by Harvey Brown, 2018). I think you should let this clear and be cautious at the end when you say it is extremely endangered, etc. Avoid overstatements, the species populations have suffered a dramatic decline in the last 10 years and wild populations are threatened, that's it.

Response：We have confirmed your information about the new assessment of *A.sinensis* and the description of endangered have been instead by “the species populations of *A.sinensis* have suffered a dramatic decline in the last 10 years and its wild populations are threatened” in the revision.

2. Comment: Please clarify which method you used to run the 19-mer analysis have you used your own method or software? Is it available for the community and reproducible?

Response：Using K-mer for estimating genome size is the routine approach based on sequencing data for estimating genomic characteristics. This GCE (genomic character estimator) was described in https://arxiv.org/abs/1308.2012 and adopted in other genome projects.

3. Comment: Regarding to this analysis how did you obtained the heterozygosity estimate of 0.32%? have you compared this with the results obtained with Genomescope, GCE or similar methods?

Response：The heterozygosity was estimated by GCE method with a Poisson model according to the same paper of genome size estimation. The heterozygosity value is various with different softwares. The Genome Scope used a mixed model and shown that the heterozygosity of *A.sinensis* is 0.6% and KAT show the number of 0.34%. We replaced the number with Genomescope result according comparative description in Genomescope’s paper https://academic.oup.com/bioinformatics/article/33/14/2202/3089939.

4. Comment: I assume the HiSeq2500 was used to sequence two pair-end libraries and, by comparing sequence and k-mer coverage, the read length was 75bp. Please, specify if the library was paired or single end and what was the read length.

Response：The library was pair-end and its read length is 150 bp. This information has been revised in this current manuscript.

5. Comment: Line 122 - you wrote "We obtained 4.8 million subreads" however, nanopore produced long reads and the term subread is used only for pacbio technology. I think this is wrong and should be corrected.

Response：The subreads is instead by nanopore long reads.

6. Comment: Nanopore sequence data: I would also like to know what was the mean accuracy of the reads. The reads that pass the filter are the ones having an average quality of at least 7, right?

Response：It is right, the mean qscore is greater than 7 (>7).

7. Comment: 133 - well, the comparison with other genomes looks very good. However, I suggest adding some comparison to the contig-level assembly for *A.sinensis* obtained with Pacbio (GCA_005392925.1 SCBG_Asin_1.0) and also *A.agallocha* (GCA_000696445.1). Mentioning these assemblies will look fair and also will highlight the higher contiguity of the one you present here.

Response：Two contig level assemblies for *Aquilaria* species have been detailed in the Table S4. The assembly derived from PacBio platform have not been published and the specific details could be got from the NCBI, so we just list N50 of contig in the compared table.

8. Comment: P.129. while polishing with pilon you haven't try to fix indels, why is this? Knowing that nanopore have recurrent errors in homopolymer tracks, I think will be an obvious thing to do. I am curious to know the reason.

Response: In Pilon software, the parameter of "fix bases" included the snps and indels. We specified it in the revised manuscript and the fix parameter has been listed as following:

```
Pilon --fix parameter :
          --fix fixlist
           A comma-separated list of categories of issues to try to fix:
             "snps": try to fix individual base errors;
             "indels": try to fix small indels;
             "bases": shorthand for "snps" and "indels" (for back compatibility);
```

9. Comment: Regarding to the preprocessing of Hi-C reads. Does Fastp merely remove the

adaptors? Other protocols, such as Arima Hi-C Mapping pipeline, try to remove some sequence at the ligation junction and keep the 5' ends to avoid the inclusion of "chimeric" reads…

Response：Fastp removed low quality reads and adaptors. For "chimeric" reads, this Hi-C pipeline is built on the basis of Hi-C pro software. Hi-C pro can detect all types of reads and get valid reads for downstream Hi-C analysis.

10. Comment: The assembly has a fairly high gene completeness as estimated by BUSCO. The mapping rate of the pair end reads reflects sequence quality. However, I would like you to perform some additional evaluations of the assembly such as a KAT stacked histogram, that reflect the efficiency and completeness of the assembly process, having into account the genome architecture (https://kat.readthedocs.io/en/latest/).

Response：The estimated assembly completeness is 90.08% in following 27-mer stacked histogram by KAT tools. Black square labeled the distribution of k-mers present in illumine paired-end data but absent in the assembly. Red labeled K-mers present in the read set and once in the assembly. Mainly of black fall into the regions of low quality and heterozygous (half of homozygous peak in x-axis).

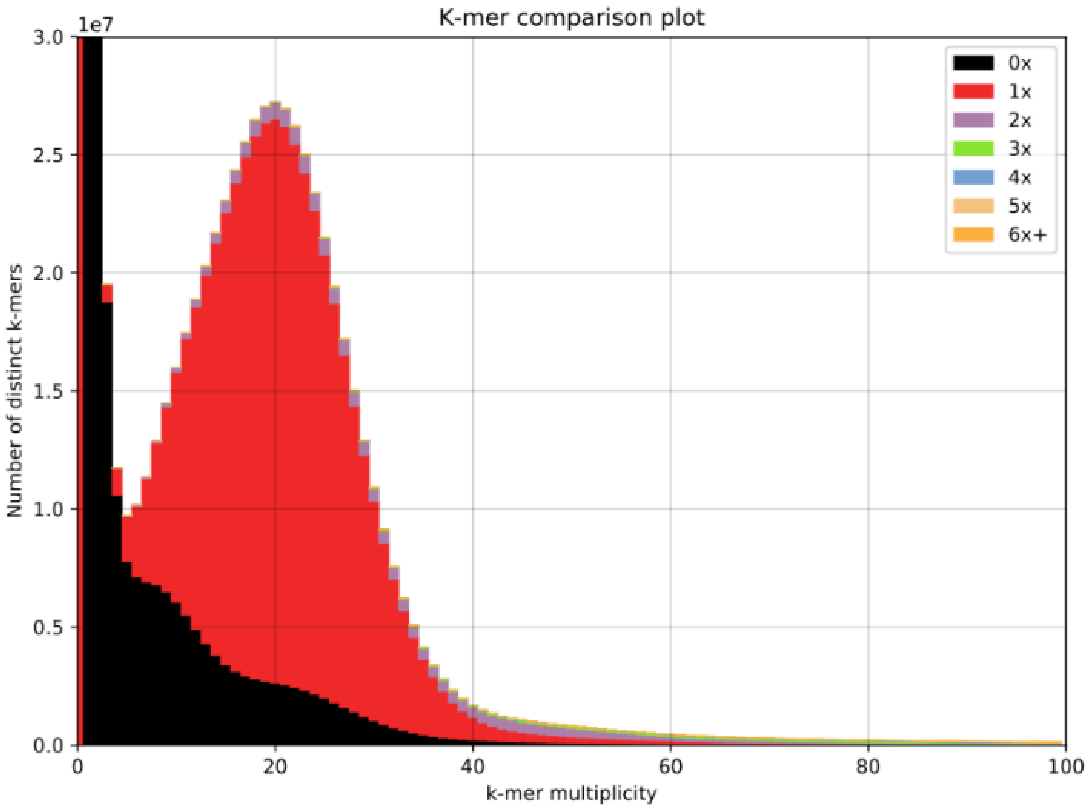

11. Comment: I would also like you to include a link to the assembly and annotation in a public repository (this will be very important for the scientific community). In fact, I would like to have a look at the genome myself. In the manuscript you merely give access to the raw reads.

Response：The assembly, annotation and other related files of *A.sinensis* genome have been uploaded to GigaDB private FTP area for reviewers within peer review stage. The Gigascience will release them to GigaDB if the manuscript were published. Actually, we are constructing a professional public repository about *Aquilaria* species, including their genomics, transcriptome and metabolism, especially for their natural products. We will let you know when our database was online in the future.

12. Comment: In the supplementary table 2: what are exactly "properly mapped" reads? Do you mean "properly paired"? After looking at ENA it seems that the illlumina is a single end library…I am a bit confused with the term "properly mapped".

Response：The illumine sequence was based on the paired-end library, we have sent this mistake to the submission staff of NCBI for correction.

13. Comment: Please correct some typos: you wrote "Predication" several times (line 193, line 216…) but the correct word is PREDICTION, (i.e. Gene Prediction). Also "sinenensis" (instead of "sinensis") or "de nove" (instead of de novo) appear later.

Response：These typos have been corrected in the revised manuscript.

14. Comment: Sentence in lines 227-235 will read much better by starting with "After removing the redundancy caused by alternative splicing variations and retaining the longest transcript of each gene, whole protein coding genes sets….OrthoMCL…[64]."

Response：This good suggestion has been adopted in the revised manuscript.

15. Comment: Please revise the English of this last pages (lines 238-296,) (specially for the last two pages, that seem to have been written on a rush)

Response：These paragraphs were revised in the revised manuscript.

16. Comment: Line 246 Gblocks filters "poorly aligned or highly divergent sites" from the alignment. I found "bias(ed) regions", as you wrote, a bit weird.

Response：We have accepted this more professional description in the revised manuscript.

17. Comment: Rather than contraction and expansion families I suggest to call them "contracted" and "expanded)

Response : All the contraction and expansion before families or gene families were replaced by contracted or expanded in the revised manuscript.

18. Comment: YOU SHOULD IMPROVE THE IMAGE RESOLUTION OF THE FIGURES the image resolution of the Figures in the manuscript (quality is very poor and numbers are often hard to read) What happened to Figure3c? in fact the supplementary images have much better quality.

Response : The image resolution of all the original figures in manuscript is 508 ×508 dpi. When they were inserted into word (.doc), their definition in generating PDF were much better than coalescing them directly after the manuscript.

19. Comment: I found the Whole genome duplication analyses weak and speculative. To my knowledge the 4DTV ratios can be used to date some evolutionary events, such as hybridization of gene family expansions/contractions. In your case the closest species to *A. sinensis* have very different genome sizes, with cocoa being almost half of sinensis (~300Mb) and Cotton with almost 3 times the sinensis genome size 2.2Gb. Have you found evidences of a ~300Mb of the agarwood genome being duplicated? Unless you have a more clear analysis of genome size evolution I suggest you to remove this from the manuscript?

Response : As your suggestion, we narrow down this section to "4DTv analysis" and shown the peak value of distribution. *A.sinensis* is the only species with chromosome-level assembly in Familia Thymelaeaceae. The cross-Familia comparison cannot fully reveal the diversity in plant genome size.

20. Comment: It would be good if you compare the divergence time estimates you have obtained with those found by other authors. By checking www.timetree.org I have confirmed that your estimate lay between the Confidence Intervals obtained by comparing several studies. It makes sense, to discuss a little bit your results or at least say that they are in concordance with previous studies.

Response : The divergence time of Asterids and Rosids, *G.hisutum* and *T.cacao* obtained in our analysis were compare with the previous studies about calibration divergence time in the revised manuscript.

21. Comment: A question about the biology of the plant. In your analyses, have you found any relationship between the immune genes and those involved in the production of agarwood or aromatic resin?

Response：This good suggestion might guide the significance to our future research. Immune gene is very important for plant defense response. But actually, we are focused on the genes about the terpene and flavonoid synthesis in the current stage, especially about the chromone synthesis, which is the sign matters for agarwood formation in *A.sinensis*. In addition, plant defense related genes were also concerned in our studies, the genes involved in abscisic acid and salicylic acid synthesis and regulation were correlative with contents of chromone in regions of agarwood formation. The ABA and SA also could induced the agarwood formation after they were injected into the stem of *A.sinensis* plants. According to the quantity, NB-LRRs and WD-40 were frequently presented in the DEG and annotation files. The expression trends of some NB-LRRs and WD-40 genes were highly correlative with agarwood formation based on time gradient. The pattern mentioned above from our previous transcriptome dataset without biological replicates (just five stages and each stage with mixture samples. because the RNA in the stem of agarwood formation was hardly accumulated), only part of them were verified with qPCR. Out further effort will focus on the LCM-based transcriptomics study.

We tried our best to improve the manuscript and made some changes in the revised manuscript. These changes will not influence the content and framework of the paper. And here we did not list the changes but marked in red in revised paper. We appreciated for you and the reviews warm work earnestly, and hope that the correction will meet with approval. Once again, thanks for your comments and suggestion.

Sincerely,

Haofu Dai

Hainan Engineering Research Center of Agarwood, Institute of Tropical Bioscience and Biotechnology, Chinese Academy of Tropical Agricultural Sciences, Rd. Xueyuan No.4, Haikou, 571101, China

Tel: +86-898-6696-1869

Email: daihaofu@itbb.org.cn
