# Supplementary material for: Genome sequence of the agarwood tree Aquilaria sinensis (Lour.) Spreng: the first chromosome-level draft genome in the Thymelaeceae family
Source: Gigascience. 2020 Mar 2;9(3):giaa013. doi: 10.1093/gigascience/giaa013 (PMC7050300; doi:10.1093/gigascience/giaa013)
Supplement: giaa013_GIGA-D-19-00288_Revision_2 [file giaa013_giga-d-19-00288_revision_2.pdf]

GigaScience

Genome sequence of agarwood tree Aquilaria sinensis (Lour.) Spreng: the first chromosome-level draft genome in the Thymelaeceae family

--Manuscript Draft--

Manuscript Number:	GIGA-D-19-00288R2
Full Title:	Genome sequence of agarwood tree Aquilaria sinensis (Lour.) Spreng: the first chromosome-level draft genome in the Thymelaeceae family
Article Type:	Data Note
Funding Information:	Central Public-interest Scientific Institution Basal Research Fund for Chinese Academy of Tropical Agricultural Sciences (17CXTD-15) — Prof. Wenli Mei
	National Natural Science Foundation of China (31870668) — Prof. Wenli Mei
	China Agriculture Research System (CARS-21) — Prof. Haofu Dai
Abstract:	Backgroud : Aquilaria sinensis (Lour.) Spreng is one of important plant resources for producing agarwood in China. The agarwood collected from the wounded Aquilaria trees have been used for aromatic or medicinal purposes in these regions from the ancient time, whereas the mechanism underlying the formation of agarwood still remained poorly understood by a lack of accurate and high-quality genetics information. Findings : We report genomic architecture of A.sinensis by an integrated strategy combining with Nanopore sequencing, Illumina sequencing and Hi-C sequencing. The final genome was approximately 726.5 Mb, which reached a high level of continuity with a contig N50 of 1.1 Mb. We combined Hi-C data with the genome assembly to generate chromosome-level scaffolds. Eight super-scaffolds corresponding to the 8 chromosomes were assembled to a final size of 716.6 Mb, with a scaffold N50 of 88.78 Mb using 1,862 contigs. Benchmarking Universal Single-Copy Orthologs evaluation reveals that the genome completeness reaches 95.27%. The repeat sequences are accounted for 59.13% and the protein-coding genes are annotated for 29,203 in the entire genome. According to phylogenetic analysis using single-copy orthologous genes, we found that A.sinensis is closely related to Gossypium hisutum and Theobroma cacao from the Malvales order, and A.sinensis was diverged from their common ancestor approximately 53.18-84.37 million years ago. Conclusions : Here, we represent the first chromosome-level genome assembly and gene annotation of A.sinensis . This study would contribute to provide valuable genetic resources for the further researches on agarwood formation mechanism, genome-assisted improvements and conservation biology of Aquilaria species.
Corresponding Author:	Haofu Dai, Ph.D<br><br>CHINA
Corresponding Author Secondary Information:	
Corresponding Author's Institution:	
Corresponding Author's Secondary Institution:	
First Author:	Xupo Ding
First Author Secondary Information:	
Order of Authors:	Xupo Ding
	Wenli Mei
	Qiang Lin
	Hao Wang

Powered by Editorial Manager® and ProduXion Manager® from Aries Systems Corporation

	Jun Wang
	Shiqing Peng
	Huiliang Li
	Jiahong Zhu
	Wei Li
	Pei Wang
	Huiqin Chen
	Wenhua Dong
	Dong Guo
	Caihong Cai
	Shengzhuo Huang
	Peng Cui
	Haofu Dai, Ph.D
**Order of Authors Secondary Information:**	
**Response to Reviewers:**	Dear editor and reviewers:<br>Thanks for your letter and the reviewers' comments concerning our manuscript entitled "Genome sequence of agarwood tree Aquilaria sinensis (Lour.) Spreng: the first chromosome-level draft genome in the Thymelaeceae family" (ID: GIGA-S-19-00378). These comments are all valuable and very helpful for improving our paper. We have studied the comments carefully and have made correction which we hope meeting with approval. Especially we upload the relevant files as supplementary materials for your reference. Revised positions are marked in red in the manuscript with track changes. The main corrections in this revised manuscript and the responds to the reviewer comments are as the following:<br><br>Response to Reviewer #1:<br>Reviewer #1: The authors have responded to all my comments.<br>The methods and the results are well described. The new version of the article is greatly improved.<br>Thanks to the authors for the improvements.<br>Response: Thanks for your previous professional comments and recommendation.<br><br>Response to Reviewer #2<br>Reviewer #2: Your first revision of the Data Note presenting the genome sequence of the Agarwood really improved the quality of the manuscript addressing most of my questions and concerns. I would be very happy to recommend it for publication of the paper if you reconsider some sentences and add some extra information to the supplementary material. Specifically, would like you to:<br>1. Comment: Add the Genome scope plot and KAT stacked histogram obtained using k=27 to the supplementary material. In their response to comment #10 you just describe the results of the KAT stacked histogram, they sound good but I would like to take a look by myself.<br>Response: The genomes cope plot and KAT stacked histogram have been upload in the supplementary material named as Genome Scope and KAT.rar for your reference.<br>2. Comment: Regarding to answer number 4 to the other reviewer (reviewer 1). I cannot really distinguish the fields properly and compare the numbers. I would like you to mention the number of breaks during the Hi-C correction in the text and add a readable table to the supplementary material.<br>Response: Breaking number indicated by Hi-C correction were added in the Supplementary Table S6. We modified the original description in the text with total breaking numbers in the Line 298.<br>3. Comment: Thanks for the clarification about the Hi-C preprocessing pipeline. Please add the reference to Servant et al. Genome Biology 2015. I cannot find it in the new revision.<br>Response: This reference has been added in the new revised manuscript.

Powered by Editorial Manager® and ProduXion Manager® from Aries Systems Corporation

	4. Comment: Line 250 at page 9: I think "comparation" should be replaced by "comparison".<br>Response: This correction is accepted in the new revised manuscript<br>5. Comment: Please reconsider the sentence at line 309 in page 11. Once again, you are mixing the vulnerability status of the species with the severely endangered one to give emphasis to a threat to the conservation of the species and the utility of the genome for management of natural populations. I suggest they could write something like: "Considering that currently, natural A.sinensis tree populations in China(?) are highly threatened due to stem heavily exploited for creating costly agarwood products, the genome assembly of A.sinensis tree presented here will provide valuable information to aid the global conservation of these precious species and contribute to understanding the mechanism of the agarwood formation, eventually will help us reveal the evolution of aromatic genes and plants." I think this sentence it is clearer, reflecting your point about the current threats without over-stating the species conservation status.<br>Response: This well description is accepted in the new revised manuscript.<br><br>We tried our best to improve the manuscript and made some changes in the revised manuscript. These changes will not influence the content and framework of the paper. And here we did not list the changes but marked in red in revised paper. We appreciated for you and the reviews warm work earnestly, and hope that the correction will meet with approval. Once again, thanks for your comments and suggestion.<br><br>Sincerely,<br><br>Haofu Dai<br><br>Hainan Engineering Research Center of Agarwood, Institute of Tropical Bioscience and Biotechnology, Chinese Academy of Tropical Agricultural Sciences, Rd. Xueyuan No.4, Haikou, 571101, China<br>Tel: +86-898-6696-1869<br>Email: daihaofu@itbb.org.cn
**Additional Information:**	
**Question**	**Response**
Are you submitting this manuscript to a special series or article collection?	No
**Experimental design and statistics**<br><br>Full details of the experimental design and statistical methods used should be given in the Methods section, as detailed in our Minimum Standards Reporting Checklist. Information essential to interpreting the data presented should be made available in the figure legends.<br><br>Have you included all the information requested in your manuscript?	Yes
**Resources**<br><br>A description of all resources used, including antibodies, cell lines, animals	Yes

and software tools, with enough information to allow them to be uniquely identified, should be included in the Methods section. Authors are strongly encouraged to cite Research Resource Identifiers (RRIDs) for antibodies, model organisms and tools, where possible.<br><br>Have you included the information requested as detailed in our Minimum Standards Reporting Checklist?	
**Availability of data and materials**<br><br>All datasets and code on which the conclusions of the paper rely must be either included in your submission or deposited in publicly available repositories (where available and ethically appropriate), referencing such data using a unique identifier in the references and in the "Availability of Data and Materials" section of your manuscript.<br><br>Have you have met the above requirement as detailed in our Minimum Standards Reporting Checklist?	Yes

Manuscript

Click here to access/download;Manuscript;Revised Manuscript_sce.docx

DATA NOTE

Genome sequence of the agarwood tree *Aquilaria sinensis* (Lour.) Spreng: the first chromosome-level draft genome in the Thymelaeaceae family

Xupo Ding[1, †], Wenli Mei[1, †], Qiang Lin[2 †], Hao Wang[1], Jun Wang[1], Shiqing Peng[3], Huiliang Li[3], Jiahong Zhu[3], Wei Li[1], Pei Wang[1], Huiqin Chen[1], Wenhua Dong[1], Dong Guo[3], Caihong Cai[1], Shengzhuo Huang[1], Peng Cui[2*], Haofu Dai[1, *]

[1] Hainan Engineering Research Center of Agarwood, Institute of Tropical Bioscience and Biotechnology, Chinese Academy of Tropical Agricultural Sciences, Rd. Xueyuan No.4, Haikou, 571101, China,

[2] Guangdong Laboratory of Lingnan Modern Agriculture, Shenzhen; Genome Analysis Laboratory of the Ministry of Agriculture; Agricultural Genomics Institute at Shenzhen, Chinese Academy of Agricultural Sciences, Rd. Pengfei No. 7, Shenzhen, 518120, China

[3] Key Laboratory of Biology and Genetic Resources of Tropical Crops of Ministry of Agriculture and Rural Affairs, Institute of Tropical Bioscience and Biotechnology; Chinese Academy of Tropical Agriculture Sciences, Rd. Xueyuan No.4, Haikou, 571101, China

Correspondence address.

Peng Cui, Agricultural Genomics Institute at Shenzhen, Chinese Academy of Agricultural Sciences, Rd. Pengfei No. 7, Shenzhen, 518120, China; Tel: +86-13828743816; E-mail: cuipeng@caas.cn;

Haofu Dai, Institute of Tropical Bioscience and Biotechnology, Chinese Academy of Tropical Agricultural Sciences, Rd. Xueyuan No.4, Haikou, 571101, China. Tel: +86-89866961869; E-mail: daihaofu@itbb.org.cn

[†]Contributed equally to this work.

ORCIDs:

Xupo Ding, 0000-0001-9195-7112

Wenli Mei, 0000-0002-4076-3497

Qiang Lin, 0000-0003-2882-7758

Hao Wang, 0000-0001-6838-6550

Jun Wang, 0000-0003-2699-5037

Shiqing Peng, 0000-0002-7279-516X
Huiliang Li, 0000-0001-9531-5504
Jiahong Zhu, 0000-0002-2201-9250
Wei Li, 0000-0001-5650-1304
Pei Wang, 0000-0002-0221-7858
Huiqing Chen, 0000-0002-7028-7858
Wenhua Dong, 0000-0002-3583-3884
Dong Guo, 0000-0001-6132-6758
Caihong Cai, 0000-0001-5249-8945
Shengzhuo Huang, 0000-0002-1559-9824
Peng Cui, 0000-0003-3076-0070
Haofu Dai, 0000-0002-5422-8137

Abstract

Backgroud: *Aquilaria sinensis* (Lour.) Spreng is one of the important plant resources involved in the production of agarwood in China. The agarwood resin collected from wounded *Aquilaria* trees has been used in Asia for aromatic or medicinal purposes from ancient times, although the mechanism underlying the formation of agarwood still remains poorly understood due to a lack of accurate and high-quality genetic information. **Findings**: We report the genomic architecture of *A.sinensis* by using an integrated strategy combining Nanopore, Illumina and Hi-C sequencing. The final genome was approximately 726.5 Mb in size, which reached a high level of continuity and a contig N50 of 1.1 Mb. We combined Hi-C data with the genome assembly to generate chromosome-level scaffolds. Eight super-scaffolds corresponding to the 8 chromosomes were assembled to a final size of 716.6 Mb, with a scaffold N50 of 88.78 Mb using 1,862 contigs. Benchmarking Universal Single-Copy Orthologs evaluation reveals that the genome completeness reached 95.27%. The repeat sequences accounted for 59.13% and 29,203 protein-coding genes were annotated in the genome. According to phylogenetic analysis using single-copy orthologous genes, we found that *A.sinensis* is closely related to *Gossypium hisutum* and *Theobroma cacao* from the Malvales order, and *A.sinensis* diverged from their common ancestor approximately 53.18-84.37 million years ago. **Conclusions**: Here, we present the first chromosome-level genome assembly and gene annotation

of *A.sinensis*. This study should contribute to valuable genetic resources for the further researches on agarwood formation mechanism, genome-assisted improvements and conservation biology of *Aquilaria* species.

Keywods: *Aquilaria sinensis*; agarwood; chromosome-level genome assembly; Hi-C sequencing; annotation

Background information

Agarwood is the fragrant resin-filled heartwood from the trees of the *Aquilaria* or *Gyrinops* genus, high-quality preparations of which are more costly than gold in the international market [1,2]. Agarwood has been used as precious incense in Buddhist, Islamic and Hindu ceremonies, and also as a traditional medicine in Chinese therapies and Ayurveda [3]. Modern pharmacological and chemical studies have indicated that sesquiterpenoid and phenylethyl chromone derivatives are the principal compounds in agarwood, many of which have been studied for potential pharmacological activities including neuroprotection, sedative, acetylcholinesterase inhibition, antioxidant, anti-bacterial and anti-inflammatory activities [4,5,6,7]. However, healthy *Aquilaria* trees generate very little agarwood unless they have been stimulated by various forms of injury or microbial infestation. In the wild, agarwood formation is usually related to natural factors such as wounding by wind or lighting damage, or gnawing by insects and fungi. [8,9]. Due to agarwood's potential medicinal and economic importance, traditional methods used for producing agarwood in Asia include chopping, nailing, boring holes, burning the stem of *Aquilaria* trees or pruning the partial trunk [10]. This has resulted in wild *Aquilaria* plants being excessively exploited, and many species are now decreasing or endangered [11].

Aquilaria sinensis has been harvested and cultured for producing agarwood, which has been used as Traditional Chinese Medicine (TCM) in China as early as the 7th century [11]. The morphological characteristics and agarwood of the *A.sinensis* are shown in Fig. 1. As the largest producer of agarwood in China, the population of *A.sinensis* has suffered a dramatic decline in the last decade and its wild populations are threatened [11, 12]. The availability of agarwood is limited by the exhaustion of its time-consuming preparation and its plant sources. Although the expression of

genes related to terpene synthesis or stress responses during agarwood formation have been described via transcriptome sequencing [2, 13, 14], the molecular mechanism of agarwood formation has remained unclear on account of lacking accurate genome information and genetic resources. Recently it has been discovered that 2-(2-phenylethyl) chromone and its derivatives were the key markers for agarwood formation in *A.sinensis* and their hypothetical biosynthetic pathway has been elucidate [8]. With reduction of *A.sinensis* plants in the wild and increasing demand in the agarwood market, it is important to interrogate the genomic background to explore the mechanism of agarwood formation and to accelerate genome-assisted improvement in breeding systems.

Herein, we sequenced and assembled the genome of *Aquilaria sinensis* (NCBI:txid210372) by a hybrid approach using Illumina short reads, Oxford Nanopore long reads and Hi-C data. We reveal the genomic features of *Aquilaria sinensis*, including repeat sequences, gene annotation and evolution. This reference genome will provide the fundamental genetic information to elucidate the metabolic formation of agarwood and facilitate the genetic research of *Aquilaria* tree.

Data Description

Genomic DNA extraction and genome size estimation

An individual plant of cultivar *Aquilaria sinensis* (Lour.) Spreng was collected from Chengxi district (110°19′24.47″E, 19°59′7.57″N), Haikou, China. After collection healthy, fresh leaves were snap frozen in liquid nitrogen, followed by preservation at -80℃in the laboratory prior to DNA extraction. High-molecular-weight plant genomic DNA was extracted from these leaves using a modified CTAB method [15]. The quality and quantity of the isolated DNA were checked by electrophoresis on a 0.75% agarose gel and a NanoDrop D-1000 spectrophotometer (NanoDrop Technologies, Wilmington, DE), and were then accurately quantified using Qubit technology. Subsequently, 150bp paired-end (PE) libraries with insert lengths of 270 bp were constructed and 49.84 Gb raw data were generated on the Illumina Hiseq2500 platform (Illumina HiSeq 2500 System, RRID:SCR_016383) using standard protocols , which were used for estimating the genome size of *A.sinensis* by using the formula: genome size= [Num (total k-mer)-Num (erro k-mer)]/ average depth of k-mer [16, 17]. Finally, the genome size of A.*sinensis* was estimated as 773.3 Mb with the total number of 19-mer approximately 3.71×10^{10} and the peak of 19-mer at the depth of 48 (Supplementary Fig. S1). The GC content of *A.sinensis* genome was 39.23%, which is considered a moderate GC level

(Supplementary Fig. S2). Meanwhile, the heterozygosity of 0.6% and repeat content of 53.12% for *A.sinensis* genome were also estimated [18].

Genomic sequencing and assembly using Nanopore long reads

One Nanopore 1D library was prepared following the Oxford Nanopore SQK-LSK 108 kit and GridION protocol [19]. Genomic DNA was first repaired and end prepped with NEBNext FFPE Repair Mix (New England Biolabs) and the NEBNext Ultra II End Repair/dA-Tailing Module (NEB). The DNA was then purified with AMPure XP beads (Beckmann Coulter) and ligated with sequencing adapters provided by ONT using concentrated T4 DNA ligase 2 M U ml^{-1} (NEB). After purification with AMPure XP beads (Beckman Coulter) using dilution buffer (ONT) and wash buffer (ONT), the library was mixed with sequencing buffer (ONT) and library loading beads (ONT) and loaded on 16 flow cells (R9.4) of GridION X5 platform (RRID:SCR_017986) [20], generating 71.3 Gb raw DNA reads (roughly 100× coverage of the genome assembly). We obtained 4.8 million nanopore long reads (67.7 Gb in total) with an N50 read length of 21.29 kb and the longest read length of 935.06 kb after removing adaptor (Supplementary Table S1).

The clean long reads obtained from Nanopore were initially assembled by wtdbg (RRID:SCR_017225) version 1.3 [21] with parameters: wtdbg -t 60 -i Passed.fastq -o Sample -H -k 17 -S 1.01 -e 4. The iterative polishing was conducted thrice by Pilon version 1.22 (RRID:SCR_014731) [22] and BWA (RRID:SCR_010910) [23] with the default parameters. The Pilon program was also run with default parameters to fill gaps, fix bases (including SNPs and indels), and correct local mis-assemblies. 99.26% of Illumina short reads were able to align to the assembled genome (Supplementary Table S2). The primary draft genome assembly was 720 Mb with a contig N50 length of 1.1 Mb and the longest contig length of 11.9 Mb (Supplementary Table S3). The contig N50 of *A.sinensis* genome was much higher than other published medicinal plants genome assemblies (Supplementary Table S4).

Hi-C library construction and chromosome-scale assembly

Hi-C, derived from chromosome conformation capture technology, is a method that probes the three-dimensional architecture of whole genomes by coupling proximity-based ligation with massively parallel sequencing [24]. The Hi-C contract matrix has been widely used for assembly correction to generate chromosome-scale scaffolds. In this work, the genomic DNA used for Hi-C

library was extracted from a fresh leaf sample of *A.sinensis* using standard methods. The crosslinked DNA from lysed cells was digested with Dpn II after cells fixed with formaldehyde. Sticky ends were biotin labeled and proximity ligated to form chimeric junctions and then physically sheared to a size of 300-500 bp. Chimeric fragments representing the original cross-linked and long-distance physical interactions were then processed into paired-end sequencing libraries after the polymerase chain reaction (PCR) amplification. The PCR cycling protocol was as following: with 95℃ for 5 minutes; cycled 18×; 4℃ for 30 seconds, 45℃ for 1 second, 70℃ for 20 seconds, and 98℃ for 30 seconds; and then held at 4℃. The products of PCR were purified according to the Hi-C protocol and then the purified DNA was sheared, end-repaired, adenylation tailed, and universal adapter ligated, and samples were indexed as described in the manufacturer's recommendations [25].

The whole genome Hi-C library was sequenced with 150bp paired-end (PE) sequencing on Illumina Hiseq 2500. A total of 714.27 million clean PE reads (~103.07 Gb, roughly 142× coverage of assembled genome) were generated after filtering adapters and low quantity reads with Fastp (version 0.12.6) and HiC-Pro (RRID:SCR_017643) [26, 27]. By mapping the Hi-C data to the Nanopore-based assembly using bowtie2 (RRID:SCR_005476) [28], we found 93.49 million unique mapped paired-end reads and 62.89 million valid interaction pairs, which respectively accounted for 26.18% and 17.61% in the clean data (Supplementary Table S5). We employed BWA and Lachesis (RRID:SCR_017644) software to align paired end reads and retain the reads aligned to 500 bp away from each restriction site [29]. According to the methods of clustering, ordering and orienting to the assembly contigs, these sequences were divided into 8 chromosome clusters and scaffolded by using Lachesis software with tuned parameters (Supplementary Table S6, Fig. 2). Finally, a heatmap of Hi-C interaction for finally assembly was produced using R (Version 3.5.3) [30, 31].

A total of 1,862 contigs were used for scaffolding by Hi-C data, which consequently generated 805 scaffolds. The Hi-C assisted chromosome-length scaffolds resulted in a final size of 716.6 Mb accounting for the 99.85% draft genome, which showed a high level of continuity with a contig N50 of 1.1 Mb and a scaffold N50 of 88.78 Mb. The final draft genome assembly of *A.sinensis* was 726.5 Mb (Supplementary Table S3). The anchor rate of contigs (>100 kb) to pseudochromosomes was

attained up to the 98.63% level based on the Hi-C assembly (Table 1). The scaffold N50 of *A.sinensis* genome was also superior to other published medicinal plant genome assemblies (Supplementary Table S4).

RNA preparation and sequencing

Iso-seq was performed for genome assembly and annotation. The sample mixe root, stem and leaf using for RNA extraction was obtained from the same plant used for Oxford Nanopore DNA sequencing and immediately snap frozen in liquid nitrogen. Total RNA was extracted from the frozen tissue using a Qiagen RNA extraction kit and the sequencing library was then prepared with SMRTbell^{TM} template prep kit 1.0 (Pacific Biosciences, Menlo Park, CA, USA) after RNA reverse transcription with SMARTer^{TM} PCR cDNA Synthesis kit and cDNA amplification with KAPA HiFi PCR kits.. Full-length transcriptome sequencing was subsequently performed using the PacBio Sequel System (RRID:SCR_017989). A total of 18,411,342 subreads were obtained from Iso-seq after raw data filtering with SMRTLING 5.1 and derived 136,050 consensus sequences, of which 94.70% (128, 854) can be aligned to the final genome of *A.sinensis* (Supplementary Table S7).

Genome quality evaluation

To evaluate the completeness of our assembly, we subjected the final assembled genome sequences to Benchmarking Universal Single-Copy Orthologs (BUSCO) version 3 (BUSCO. *Embryophyta* odb 10, RRID:SCR_015008) (BUSCO, *Embryophyta* odb 10) [32,33]. Overall, 95.27% of 1375 expected embryophyta genes were identified in our genome assembly as the complete and partial BUSCO profiles. Among these identified 1310 complete expected embryophyta genes, 1202 and 108 were identified as single copy and duplicated copies respectively (Supplementary Table S8).

Repeat sequences within the *A.sinensis* genome assembly

Transposable elements (TEs) and tandem repeats were identified with both homology-based annotation and *de novo* methods. Consensus sequences of repetitive elements were *de novo* identified and classified using the software package RepeatModeler version 1.04 (RepeatModeler, RRID:SCR 015027) [34]. RepeatMask version 3.2.9 (RepeatMasker, RRID:SCR 012954) [34], RepeatProteinMasker [35] and TRF [36] were used to discover and identify repeats within the respective genomes. Furthermore, simple sequence repeat (SSR) in the *A.sinensis* genome were also classified with MISA (MISA, RRID:SCR 010765) [37]. The results showed that *de novo* predicted repeats were more recently active than Repbase [38] predicted repeats (Supplementary Fig. S3). The

identified repeat sequences in the *A.sinensis* genome assembly accounted for 59.13% and total length of those accounted for 425.87 Mb (Supplementary Table S9). In particular, the details showed that long terminal repeats (LTR) were the most abundant repeat type and that two non-LTR retrotransposons, short interspersed nuclear element (SINE) and long interspersed nuclear element (LINE) [39], had the lowest proportions in the final assemblies. In addition, 13.12% of repeat sequences could not be classified (Table 2). Total 367,251 SSRs are identified from the draft assembly in 675 scaffolds. Mononucleotides (64.71%), dinucleotides (18.19%), and trinucleotides (12.46%) comprised nearly 96% of SSRs in our assembly (Supplementary Table S10).

Gene prediction and annotation

Three strategies were used for gene prediction. Augustus version 3.2.3 (Augustus, RRID:SCR 008417) [40], GlimmHmm [41] and GeneID (GeneID, RRID:SCR 002473) [42] were used for *ab initio* gene prediction, using model training based on CDS from *Corchorus olitorius* (COLO4_1.0) [43], *Durio zibethinus* (Duzib1.0) [44], *Gossypium hirsutum* (ASM98774v1) [45], *Herrania umbratica* (ASM216827v2) [46], *Theobroma cacao* (Cirollo_cocoa_geneoe_v2) [47] and *Arabidopsis thaliana* (TAIR10) [48]. GeneWise (GeneWise, RRID:SCR 015054) [49] and GeMoMa [50] were used for homology prediction. PASA (PASA, RRID:SCR 014656) [51] and Tophat (TopHat, RRID:SCR 013035) [52] were used for gene structural prediction based on EST and cDNA sequences. Finally, the total gene prediction was obtained from the union of these three strategies with EVM [51] and filtering the transposable elements with Transposon PSI (Transposon, RRID:SCR 001159) [53]. RNA-seq data of mixed tissues was mapped with the annotation of the reference genome using MatchAnnot [54], respectively.

The final annotation was composed of 29,203 genes models with an average of 3,177.62 bp transcripts and 1,114.16 bp coding sequence, each gene containing 5.02 exons with the average length of 222.09 bp. The comparative information of genes from *A.sinensis* and six closely related plants was also calculated (Supplementary Table S11), including their distributions of CDS and gene length, exon and intron length, exon and intron number (Supplementary Fig. S4). Genes were characterized for their putative function by performing the Blastall [55] and KAAS [56] search of the peptide sequences against the Swiss-Prot (Swiss-Prot, RRID:SCR_002380) [57], NR [58], TrEMBL (TrEMBL, RRID:SCR_002380) [57], KEGG (the Kyoto Encyclopedia of Genes and Genomes, Orthology) database (KEGG, RRID:SCR_012773) [59], COG (Clusters of Orthologous

Groups) database (COG, RRID:SCR_007273) [60] and the Gene Ontology (GO) database (GO, RRID:SCR_002811) [61]. Protein conservative models and motifs prediction were used InterProScan version 5.2 (InterproScan, RRID:SCR_005829) [62]. Of these 29,203 protein-coding genes, 82.64% have functional annotation. Summary of hits of database research was as the following: Swiss-Prot (19,586; 67.07%), NR (24,097; 82.52%), TrEMBL(23,455; 80.32%), KEGG (8,494; 29.09%), COG (13,592; 46.54%), GO (14,019, 78.70%) and InterProScan (20,031; 68.59%) (Supplementary Table S12). In addition, we also identified 207 miRNAs, 34 rRNAs, 173 tRNAs and 1,173 snRNAs via Rfam non-coding RNA (ncRNA) database (Rfam, RRID:SCR 007891) [63], tRNAscan-SE (tRNAscan-SE, RRID:SCR 010835) [64] and RNAmmer [65]. The average length, total length and percentage of ncRNAs in *A.sinensis* genome were further assessed (Supplementary Table S13). In addition, 48.61% of predicted genes (14,197) were supported by Iso-seq transcripts (Supplementary Table S14).

Gene family identification and phylogenetic tree construction

By keeping the longest transcript for each gene, whole protein coding genes sets from *A.sinensis* genome and other 12 representative plant genomes including *G.hirutum* (ASM98774v1), *A.thaliana* (TAIR10), *T.cacao* (Cirollo_cocoa_geneoe_v2), *Cephalotus follicularis* (Cfol_1.0), *Citrus clementina* (Citrus_clementina_v1.0), *Cucurbita pepo* (ASM280686v2), *Eucalyptus grandis* (Egrandis1_0), *Glycine max* (Glycine_max_v2.1), *Helianthus annuus* (HanXRQr 1.0), *Populus euphratica* (PopEup_1.0), *Quercus suber* (CorkOak 1.0),and *Vitis vinifera* (assembly 12X) were used to construct a global gene family classification with all-vs-all BLASTP(1e^{-5} cutoff, Blast+ v2.3.056) and OrthoMCL version 2.0.9 (Ortholog Groups of Protein Sequences, RRID: SCR_007839) [66]. The default settings were used for BLASTP and OrthoMCL. In our assembly, 21,955 genes were clustered into 13,713 gene families. Gene family analysis also revealed that 789 gene families and 7,248 genes were unique to *A.sinensis* in above comparison (Fig.3a and Supplementary Table S15). Of these, 9,615 gene families were shared among *A.sinensis* and four representative species (*G.hirsutum* from Malvaceae, *C.olitorius* from Tiliaceae, *T.cacao* from Sterculiaceae and *A.thaliana* as the model plant from Cruciferae), whereas 804 gene families were unique to *A.sinensis* genome (Fig. 3b). Malvaceae, Tiliaceae and Sterculiaceae are beyond the order Malvales and the Thymelaeceae family is also divided into order Malvales in APG IV [67].

Single-copy genes or the orphan genes with only single copy in genome during duplication and

evolution of species are highly conserved, which are generally used for establishing genetic relationship and origin of species. Alignment of single-copy gene was performed with protein sequences by Mafft (Mafft, RRID: SCR_011811) [68], then poorly aligned and highly divergent sites were filtering with the Gblocks (Gblocks, RRID: SCR_015945) [69] and the final CDSs were used for evolutional analyses by RaxML with GTRGAMMA model (RaxML, RRID: SCR_006086) [70]. The bootstrap was 100 and *Helianthus annuus* from the Asterids was the outgroup [71]. We constructed a phylogenetic tree and estimated the divergence time of 13 plants by 89 single-copy gene families with the MCMCTREE of PAML [72] (Supplementary Fig. S5) (Parameters: clock = 2, RootAge = <100.6, model = 7, BDparas = 1 1 0 , kappa gamma = 6 2 , alpha gamma = 1 1, rgene gamma = 2 3.18, sigma2 gamma = 1 1.3; Divergence time of Asterids and Rosids (~118 Mya) was used for calibration [71]). The divergence time between *A.sinensis* and *A.thaliana* was estimated as 82.14 (67.63-93.99) million years ago (Mya), and the divergence time between *A.sinensis* and the common ancestor of *G.hisutum* and *T.cacao* from Malvales order was approximately 69.64 (53.18-84.37) million years ago (Fig. 3c and Supplementary Fig. S6), whereas the divergence time between *G.hisutum* and *T.cacao* were determined as 31.33-69.23 million years ago in our analysis and this is concordance with the previous studies [73, 74].

Gene family expansion and contraction

Expansion and contraction of defining gene family is an important driver of metabolite variation and species adaptation during plant evolution [75]. We determined the expansion and contraction of orthologous gene families in *A.sinensis* genome by CAFÉ 2.2 (CAFÉ, RRID: SCR_005983) with default parameters[76]. We inferred 53 expanded families and 117 contracted families with *A.sinensis* genome after comparing 11,855 gene families across all 13 species (Fig.3c and Supplementary Table S16). Using Blast2GO (B2G, RRID: SCR_005828) to enrich the ontology categories (GO and KEGG terms), The expanded gene families involved in the pathways of plant circadian rhythm, tricarboxylic acid cycle, propanoate metabolism, Ribosome biogenesis and aminoacyl-tRNA biosynthesis (Supplementary Table S17 and Fig. S7), and the contracted gene families mapped pathways of starch/sucrose metabolism, sesquiterpenoid and triterpenoid biosynthesis and linoleic acid metabolism (Supplementary Table S18 and Fig. S8).

4DTv Distribution

We used MCScanX to identify the syntenic regions [77], with the longest isoform for each gene selected for this exercise. The top five mutual hits of the BLASTP results in gene family analysis were used as input. Only the syntenic segments that have more than five gene pairs were considered for four-fold degenerate synonymous sites (4DTv) calculation. Pairwise sequence was aligned using MUSCLE [78]. Raw 4DTv values were corrected for possible multiple transversions at the same site. Based on 4DTv distribution, a large accumulation of gene duplications is evident in the *A.sinensis* genome and distinct from the scenarios in *A.thaliana*, *M.truncatula* and *V.vinifera* (Fig. 3d).

Conclusions

In summary, a high-quality *de novo* genome assembly and in-depth characterization of *A.sinensis*, combining Nanopore single-molecule long reads and Hi-C has been provided in this study. The final assembly was approximately 726.5 Mb in size, which was slightly smaller than the k-mer estimated genome size of 773.3 Mb. The Hi-C data was used to identify and revise 230 mis-assemblies and assign the contigs into chromosome-scale scaffolds. This consequently generated an assembly with a high level of continuity with a contig N50 of 1.1 Mb and a scaffold N50 of 88.78 Mb. We also predicted 29,203 protein-coding genes from the final assembly and 82.64% (24,133 genes) of all protein-coding genes were annotated. We estimated that the divergence time between *A.sinensis* and its common ancestor *G.hisutum* and *T.cacao* from the Malvales order was approximately 53.18-84.37 million years ago. The genome of *A.sinensis* seems to have experienced a recent whole-genome duplication event after the K-T boundary [79]. The chromosome-level genome assembly of *A.sinensis* is also the first high-quality genome in the Thymelaeceae family. Considering that wild *A.sinensis* tree populations are currently highly threatened due to heavily exploition for the production of commercial valuable agarwood products, the genome assembly of *A.sinensis* tree presented here will provide valuable information to aid the global conservation of these precious biological resources. And contributing to the understanding the mechanism of agarwood formation, and assisting will help us reveal the evolution of aromatic genes and plants.

Availability of supporting data

Supporting data and materials are available in the *GigaScience* GigaDB database (GigaDB, RRID:SCR 004002) [80], with the raw genomics and transcriptome sequences deposited in the NCBI Sequence Read Archive (SRA) database under the BioProject accession number

PRJNA556948 and BioSample accession number SAMN12385133.

Additional files

S Fig.1 K-mer (k = 19) analysis for estimating the size of the *Aquilaria sinensis* genome.

S Fig.2 GC content and average sequencing depth of the Illumina sequencing data used for genome estimation.

S Fig.3 Distribution of sequence divergence rates of different TE types with Repbase (A) and *de novo* (B) methods in *Aquilaria sinensis* genome.

S Fig.4 Distribution of gene elements in *Aquilaria sinensis* genome and other six plant genome.

S Fig.5 Phylogenetic tree of 13 plant species including *Aquilaria sinensis.*

S Fig.6 Estimation of divergence time of 13 plant species investigated in the present study. The colored numbers on the nodes are the divergence time from present (million years ago). Numbers in the bracket indicate the 95% confidence interval of the divergence time.

S Fig.7 GO enrichment of expansion gene families in *Aquilaria sinensis* genome.

S Fig.8 GO enrichment of contraction gene families in *Aquilaria sinensis* genome.

S Table 1 Summary of Nanopore sequencing for *Aquilaria sinensis* genome.

S Table 2 Supporting of Illumina data for Nanopore data in *Aquilaria sinensis* genome assembled.

S Table 3 Statistics of the results of *Aquilaria sinensis* genome assembly before Hi-C mapping.

S Table 4 Comparisons of genome assemblies of medicinal plants based on descending Contig N50.

S Table 5 Summary of mapping status of Hi-C data.

S Table 6 Statistics of initial and final assembly with Hi-C.

S Table 7. Mapping result of Iso-seq from *Aquilaria sinensis*.

S Table 8 Statistics of BUSCO evolution for *Aquilaria sinensis* genome.

S Table 9 Statistics of repeat sequence in *Aquilaria sinensis* genome via different methods.

S Table 10 Statistics of SSRs in *Aquilaria sinensis* genome sequences.

S Table 11 Statistics of characters of gene models in *Aquilaria sinensis* and other six plant genome.

S Table 12 The annotated genes of *Aquilaria sinensis* which can be functionally classified in each corresponding database.

S Table 13 Noncoding RNA annotation in the *Aquilaria sinensis* genome.

S Table 14. Annotation of Iso-seq and compared with genome annotation of the *Aquilaria sinensis* genome.

S Table 15 Summary of gene families among 13 plant species.

S Table 16 Summary of gene families changes among 13 species.

S Table 17 KEGG mapping of expansion gene families in *Aquilaria sinensis* genome.

S Table 18 KEGG mapping of contraction gene families in *Aquilaria sinensis* genome.

Abbreviation

IUCN: International Union for Conservation of Nature and Natural resources; SMRT: single molecular real time; Iso-seq: Isoform sequencing; BUSCO: Benchmarking Universal Single-Copy Orthologs; Hi-C: high-throughput chromosome conformation capture; TE: transposable element; EVM: EVidenceModeler;NR: PASA: Program to Assemble Spliced Alignments; Nr: NCBI non-redundant protein database; PCR: polymerase chain reaction; Pfam: protein families; TrEMBL: Translated EMBL-Bank; MYA: million years ago. CAFÉ: Computational Analysis of gene Family Evolution; MRCA: most recent common ancestor; K-T: Cretaceous-Tertiary.

Competing interests

The authors declare that they have no competing interests.

Funding

This work was supported by the Central Public-interest Scientific Institution Basal Research Fund for Chinese Academy of Tropical Agricultural Sciences (17CXTD-15), the National Natural Science Foundation of China (31870668) and the China Agriculture Research System (CARS-21). We are grateful to NextOmics Co., Ltd. (Wuhan, China) for providing technical help.

Author contributions

H.F.D., P.C. and W.L.M. conceptualized the research program. X.P.D., W.L.M. and S.Q.P. designed experiments and coordinated the program. S.Z.H. collected the specimens and J.W. took the photos. H.L.L and J.H.Z. extracted the DNA. X.P.D., Q.L., P.W., P.C., W.L., H.Q.C., W.H.D., D.G. and C.H.C were partially involved either experiments or data analysis. X.P.D. and Q.L. wrote the manuscript. All authors read and approved the final manuscript.

References

1. Kumeta Y and Ito M. Characterization of α-humulene synthases responsible for the production of sesquiterpenes

induced by methyl jasmonate in *Aquilaria* cell culture. Journal of Natural Medicines 2016;70(3): 452-459.

2. Xu Y, Zhang Z, Wang M, et al. Identification of genes related to agarwood formation: transcriptome analysis of healthy and wounded tissues of *Aquilaria sinensis*. BMC Genomics 2013;14(1): 227.

3. Naef R. The volatile and semi- volatile constituents of agarwood, the infected heartwood of *Aquilaria* species: a review. Flavour and Fragrance Journal 2011;26(2): 73-87.

4. Liao G, Mei WL, Kong FD, et al. 5, 6, 7, 8-Tetrahydro-2-(2-phenylethyl) chromones from artificial agarwood of *Aquilaria sinensis* and their inhibitory activity against acetylcholinesterase. Phytochemistry 2017;139: 98-108.

5. Hashim Y Z H Y, Kerr P G, Abbas P, et al. *Aquilaria* spp.(agarwood) as source of health beneficial compounds: A review of traditional use, phytochemistry and pharmacology. Journal of Ethnopharmacology 2016, 189: 331-360.

6. Ma CT, Eom T, Cho E, et al. Aquilanols A and B, macrocyclic humulene-type sesquiterpenoids from the agarwood of *Aquilaria malaccensis*. Journal of Natural Products 2017, 80(11): 3043-3048.

7. Yang L, Yang YL, Dong WH, et al. Sesquiterpenoids and 2-(2-phenylethyl) chromones respectively acting as α-glucosidase and tyrosinase inhibitors from agarwood of an *Aquilaria* plant. Journal of Enzyme Inhibition and Medicinal Chemistry 2019;34(1): 853-862.

8. Liao G, Dong W H, Yang J L, et al. Monitoring the chemical profile in agarwood formation within one year and speculating on the biosynthesis of 2-(2-phenylethyl) chromones. Molecules 2018;23(6): 1261.

9. Chhipa H, Chowdhary K, Kaushik N. Artificial production of agarwood oil in *Aquilaria* sp. by fungi: a review. Phytochemistry Reviews 2017;16(5): 835-860.

10. Azren P D, Lee S Y, Emang D, et al. History and perspectives of induction technology for agarwood production from cultivated Aquilaria in Asia: a review. Journal of forestry research 2019, 30(1): 1-11.

11. Harvey-Brown, Y. *Aquilaria sinensis*. The IUCN Red List of Threatened Species 2018. 2018; e.T32382A2817115. http://dx.doi.org/10.2305/IUCN.UK.2018-2.RLTS.T32382A2817115.en

12. Wang Y, Zhan D F, Jia X, et al. Complete chloroplast genome sequence of *Aquilaria sinensis* (Lour.) Gilg and evolution analysis within the Malvales order. Frontiers in Plant Science 2016;7: 280.

13. Wang X, Gao B, Liu X, et al. Salinity stress induces the production of 2-(2-phenylethyl) chromones and regulates novel classes of responsive genes involved in signal transduction in *Aquilaria sinensis* calli. BMC plant biology 2016;16(1): 119.

14. Wang X, Zhang Z, Dong X, et al. Identification and functional characterization of three type III polyketide synthases from *Aquilaria sinensis* calli. Biochemical and biophysical research communications 2017;486(4):

1040-1047.

15. Porebski S, Bailey LG, Baum BR. Modification of a CTAB DNA extraction protocol for plants containing high polysaccharide and polyphenol components. Plant Molecular Biology Reporter 1997;15(1): 8-15.

16. Liu B, Shi Y, Yuan J, et al. Estimation of genomic characteristics by analyzing k-mer frequency in de novo genome projects. arXiv preprint arXiv 2013: 1308.2012. https://arxiv.org/abs/1308.2012.

17. Ding X, Mei W, Huang S, et al. Genome survey sequencing for the characterization of genetic background of *Dracaena cambodiana* and its defense response during dragon’s blood formation. PloS ONE 2018;13(12): e0209258.

18. Vurture G W, Sedlazeck F J, Nattestad M, et al. GenomeScope: fast reference-free genome profiling from short reads. Bioinformatics 2017; 33(14): 2202-2204.

19. Leggett RM and Clark MD. A world of opportunities with nanopore sequencing. Journal of Experimental Botany 2017;68(20): 5419-5429.

20. Schmidt MHW, Vogel A, Denton AK, et al. *De novo* assembly of a new *Solanum pennellii* accession using nanopore sequencing. The Plant Cell 2017;29(10): 2336-2348.

21. Ruan, J., Li, H. Fast and accurate long-read assembly with wtdbg2. Nat Methods (2019). https://doi.org/10.1038/s41592-019-0669-3.

22. Walker B J, Abeel T, Shea T, et al. Pilon: an integrated tool for comprehensive microbial variant detection and genome assembly improvement. PloS ONE 2014;9(11): e112963.

23. Li H, Durbin R. Fast and accurate long-read alignment with Burrows-Wheeler transform. Bioinformatics 2010; 26(5): 589-595.

24. Lieberman-Aiden E, Van Berkum NL, Williams L, et al. Comprehensive mapping of long-range interactions reveals folding principles of the human genome. Science 2009;326(5950): 289-293.

25. Xu CQ, Liu H, Zhou SS, et al. Genome sequence of *Malania oleifera*, a tree with great value for nervonic acid production. GigaScience 2019;8(2): giy164.

26. Chen S, Zhou Y, Chen Y, et al. fastp: an ultra-fast all-in-one FASTQ preprocessor. Bioinformatics 2018;34(17): i884-i890.

27. Servant N, Varoquaux N, Lajoie BR, et al. (2015). HiC-Pro: an optimized and flexible pipeline for Hi-C data processing. Genome biology 2015;16(1), 259.

28. Langmead B, Salzberg SL. Fast gapped-read alignment with Bowtie 2. Nature Methods 2012;9(4): 357.

29. Burton J N, Adey A, Patwardhan R P, et al. Chromosome-scale scaffolding of de novo genome assemblies based

on chromatin interactions. Nature Biotechnology 2013;31(12): 1119.

30. R Core Team. R: A language and environment for statistical computing. 2019. https://www.R-project.org/.

31. Yin D, Ji C, Ma X, et al. Genome of an allotetraploid wild peanut *Arachis monticola*: a de novo assembly. GigaScience, 2018;7(6): giy066.

32. Simão FA, Waterhouse RM, Ioannidis P, et al. BUSCO: assessing genome assembly and annotation completeness with single-copy orthologs. Bioinformatics 2015;31(19): 3210-3212.

33. Waterhouse RM, Seppey M, Simão FA, et al. BUSCO applications from quality assessments to gene prediction and phylogenomics. Molecular Biology and Evolution 2017;35(3): 543-548.

34. Bedell JA, Korf I, Gish W. MaskerAid: a performance enhancement to RepeatMasker. Bioinformatics 2000;16(11): 1040-1041.

35. Allred DB, Cheng A, Sarikaya M, et al. Three-dimensional architecture of inorganic nanoarrays electrodeposited through a surface-layer protein mask. Nano Letters 2008;8(5): 1434-1438.

36. Benson G. Tandem repeats finder: a program to analyze DNA sequences. Nucleic Acids Research 1999;27(2): 573-580.

37. Thiel T, Michalek W, Varshney R, et al. Exploiting EST databases for the development and characterization of gene-derived SSR-markers in barley (*Hordeum vulgare* L.). Theoretical and Applied Genetics 2003;106(3): 411-422.

38. Jurka J, Kapitonov V V, Pavlicek A, et al. Repbase Update, a database of eukaryotic repetitive elements[J]. Cytogenetic and Genome Research 2005;110(1-4): 462-467.

39. Yang L, Scott LA, Wichman HA. Tracing the history of LINE and SINE extinction in sigmodontine rodents. Mobile DNA 2019;10(1): 22.

40. Stanke M, Steinkamp R, Waack S, et al. AUGUSTUS: a web server for gene finding in eukaryotes. Nucleic Acids Research 2004;32(suppl_2): W309-W312.

41. Majoros WH, Pertea M, Salzberg SL. TigrScan and GlimmerHMM: two open source ab initio eukaryotic gene-finders. Bioinformatics 2004;20(16): 2878-2879.

42. Blanco E, Parra G, Guigó R. Using geneid to identify genes. Current Protocols in Bioinformatics 2007;18(1): 4.3. 1-4.3. 28.

43. Islam MS, Saito JA, Emdad EM, et al. Comparative genomics of two jute species and insight into fibre biogenesis. Nature Plants 2017;3(2): 16223.

44. Teh BT, Lim K, Yong CH, et al. The draft genome of tropical fruit durian (*Durio zibethinus*). Nature Genetics

2017;49(11): 1633.

45. Li F, Fan G, Lu C, et al. Genome sequence of cultivated Upland cotton (*Gossypium hirsutum* TM-1) provides insights into genome evolution. Nature Biotechnology 2015;33(5): 524.

46. *Herrania umbratica*. https://www.ncbi.nlm.nih.gov/genome/55117

47. Argout X, Martin G, Droc G, et al. The cacao Criollo genome v2. 0: an improved version of the genome for genetic and functional genomic studies. BMC Genomics 2017;18(1): 730.

48. Michael TP, Jupe F, Bemm F, et al. High contiguity *Arabidopsis thaliana* genome assembly with a single nanopore flow cell. Nature Communications 2018;9(1): 541.

49. Birney E, Durbin R. Using GeneWise in the Drosophila annotation experiment. Genome Research 2000;10(4): 547-548.

50. Keilwagen J, Hartung F, Grau J. GeMoMa: Homology-Based Gene Prediction Utilizing Intron Position Conservation and RNA-seq Data. Gene Prediction. Humana, New York, NY, 2019: 161-177.

51. Haas BJ, Salzberg SL, Zhu W, et al. Automated eukaryotic gene structure annotation using EVidenceModeler and the Program to Assemble Spliced Alignments. Genome Biology 2008;9(1): R7.

52. Trapnell C, Pachter L, Salzberg SL. TopHat: discovering splice junctions with RNA-Seq. Bioinformatics 2009;25(9): 1105-1111.

53. Yagi M, Kosugi S, Hirakawa H, et al. Sequence analysis of the genome of carnation (*Dianthus caryophyllus* L.). DNA Research 2013;21(3): 231-241.

54. Hu J, Uapinyoying P, Goecks J. Interactive analysis of Long-read RNA isoforms with Iso-Seq Browser. BioRxiv 2017: 102905.

55. Coordinators NR. Database resources of the national center for biotechnology information. Nucleic Acids Research 2017;45: D12.

56. Moriya Y, Itoh M, Okuda S, et al. KAAS: an automatic genome annotation and pathway reconstruction server[J]. Nucleic Acids Research 2007;35(suppl_2): W182-W185.

57. Boeckmann B, Bairoch A, Apweiler R, et al. The SWISS-PROT protein knowledgebase and its supplement TrEMBL in 2003. Nucleic Acids Research 2003;31(1): 365-370.

58. Yu K, Zhang T. Construction of customized sub-databases from NCBI-nr database for rapid annotation of huge metagenomic datasets using a combined BLAST and MEGAN approach. PLoS ONE 2013;8(4): e59831.

59. Kanehisa M, Furumichi M, Tanabe M, et al. KEGG: new perspectives on genomes, pathways, diseases and drugs. Nucleic Acids Research 2016;45(D1): D353-D361.

60. Kristensen DM, Kannan L, Coleman MK, et al. A low-polynomial algorithm for assembling clusters of orthologous groups from intergenomic symmetric best matches. Bioinformatics 2010;26(12): 1481-1487.

61. Gene Ontology Consortium. Gene ontology consortium: going forward. Nucleic Acids Research 2014;43(D1): D1049-D1056.

62. Hunter S, Apweiler R, Attwood TK, et al. InterPro: the integrative protein signature database. Nucleic Acids Research 2008;37(suppl_1): D211-D215.

63. Griffiths-Jones S, Moxon S, Marshall M, et al. Rfam: annotating non-coding RNAs in complete genomes[J]. Nucleic Acids Research 2005;33(suppl_1): D121-D124.

64. Lowe TM, Eddy SR. tRNAscan-SE: a program for improved detection of transfer RNA genes in genomic sequence. Nucleic Acids Research 1997;25(5): 955-964.

65. Lagesen K, Hallin P, Rødland E A, et al. RNAmmer: consistent and rapid annotation of ribosomal RNA genes. Nucleic Acids Research 2007;35(9): 3100-3108.

66. Li L, Stoeckert CJ, Roos DS. OrthoMCL: identification of ortholog groups for eukaryotic genomes. Genome Research 2003;13(9): 2178-2189.

67. Chase MW, Christenhusz MJM, Fay MF, et al. An update of the Angiosperm Phylogeny Group classification for the orders and families of flowering plants: APG IV. Botanical Journal of the Linnean Society 2016;181(1): 1-20.

68. Katoh K, Standley D M. MAFFT multiple sequence alignment software version 7: improvements in performance and usability. Molecular Biology and Evolution 2013;30(4): 772-780.

69. Castresana, J. Selection of Conserved Blocks from Multiple Alignments for Their Use in Phylogenetic Analysis. Molecular Biology and Evolution 2000 17(4):540-552.

70. Stamatakis, A. RAxML-VI-HPC: maximum likelihood-based phylogenetic analyses with thousands of taxa and mixed models. Bioinformatics 2006;22(21):2688-2690.

71. Badouin H, Gouzy J, Grassa CJ, et al. The sunflower genome provides insights into oil metabolism, flowering and Asterid evolution. Nature 2017;546(7656): 148.

72. Yang Z. PAML 4: phylogenetic analysis by maximum likelihood. Molecular Biology and Evolution 2007;24(8): 1586-1591.

73. Li F, Fan G, Lu C, et al. Genome sequence of cultivated Upland cotton (*Gossypium hirsutum* TM-1) provides insights into genome evolution. Nature biotechnology 2015; 33(5): 524.

74. Teh B T, Lim K, Yong C H, et al. The draft genome of tropical fruit durian (*Durio zibethinus*). Nature genetics

2017; 49(11): 1633.

75. Denoeud F, Carretero-Paulet L, Dereeper A, et al. The coffee genome provides insight into the convergent evolution of caffeine biosynthesis. Science 2014;345(6201): 1181-1184.

76. De Bie T, Cristianini N, Demuth J P, et al. CAFE: a computational tool for the study of gene family evolution. Bioinformatics 2006;22(10): 1269-1271.

77. Wang Y, Tang H, DeBarry J D, et al. MCScanX: a toolkit for detection and evolutionary analysis of gene synteny and collinearity. Nucleic Acids Research 2012;40(7): e49-e49.

78. Edgar R C. MUSCLE: multiple sequence alignment with high accuracy and high throughput. Nucleic Acids Research 2004;32(5): 1792-1797.

79. Fawcett J A, Maere S, Van De Peer Y. Plants with double genomes might have had a better chance to survive the Cretaceous–Tertiary extinction event. Proceedings of the National Academy of Sciences 2009;106(14): 5737-5742.

80. Ding X; Mei W; Lin Q; Wang H; Wang J; Peng S; Li H; Zhu J; Li W; Wang P; Chen H; Dong W; Guo D; Cai C; Huang S; Cui P; Dai H (2020): Supporting data for "Genome sequence of agarwood tree *Aquilaria sinensis* (Lour.) Spreng: the first chromosome-level draft genome in the Thymelaeceae family" GigaScience Database. http://dx.doi.org/10.5524/100702

Figure legends

Figure 1: Morphological characteristic of *Aquilaria sinensis*. (a) mature tree; (b) flower; (c) fruit; (d) seed; (e) cracked seed; (f) agarwood generation; (g) agarwood. The images b, c, d and e were cpatured using a stereoscopic fluorescence microscope (Olympus SZX16, Pittsburgh, PA) under the dark field. All the photos were taken by Dr. Jun Wang and processed by Dr. Xupo Ding.

Figure 2: Hi-C interaction matrix for *A.sinensis* genome assembly using 8 clusters.

Figure 3: Comparative genomic analysis of *Aquilaria sinensis* and other plant species. (a) Distribution of genes and gene families of 13 plant species we investigated. (b) A Venn diagram showing the distribution of shared gene families among the Malvales plants *Aquilaria sinensis* (agarwood), *Theobroma cacao* (cocoa), *Gossypium hirsutum* (cotton), *Corchorus olitorius* (jute) and the model plant *Arabidopsis thaliana* (Arabidopsis). (c) Divergence time estimation and gene families changes among 13 plant species. The black number at each node denotes estimated

divergence time from present (million years ago). The blue number at the root (11885) denotes the total number of gene families predicted in the most recent common ancestor (MRCA) and the green/red numbers around each branch denote gene family gain/loss number. The red nodes indicate the known divergence time of Asterids and Rosids. (d) 4dTv distribution in selected assemblies of *A.sinensis*, *A.thaliana*, *O.sativa*, *M.truncatula* and *V.vinifera*. 4dTv, transversion substitutions at four-fold degenerate sites.

Table legends

Table 1. Statistics of the final genome assembly for *Aquilaria sinensis.*

Table 2. Statistics of transposable elements in *Aquilaria sinensis* genome sequences.

Table 1. Statistics of the final genome assembly for Aquilaria sinensis.
Click here to access/download;Table;Table 1.docx

Table 1. Statistics of the final genome assembly for *Aquilaria sinensis.*

Statistics Type	Contig Length (bp)	Contig Number	Scaffold Length (bp)	Scaffold Number
N50	1,058,652	164	88,784,932	4
N60	726,407	246	86,380,100	5
N70	495,861	366	84,956,755	6
Longest	11,913,571	1	109,870,270	1
Total	720,187,708	2,015	726,587,161	9
Length>=1kb	720,187,482	2,013	726,587,161	9
Length>=2kb	720,179,880	2,008	726,587,161	9
Length>=5kb	720,112,854	1,991	726,587,161	9

Table 2. Statistics of transposable elements in *Aquilaria sinensis* genome sequences.

	Repbase TEs		Mips-REdat TEs		TE proteins		RepeatModeler		Combined TEs	
Type	Length (Mb)	% in genome	Length (Mb)	%in genome	Length (Mb)	% in Genome	Length (Mb)	% in genome	Length (Mb)	% in genome
DNA	13223408	1.84	1392136	0.19	10456270	1.45	28698131	3.98	38895471	5.4
LINE	2916904	0.41	253492	0.04	7680548	1.07	6394899	0.89	12239695	1.7
LTR	73748923	10.24	22973865	3.19	75336839	10.46	138348032	19.21	192609862	26.74
SINE	2232	0	1145	0	0	0	0	0	4539	0
Other	6189190	0.86	380555	0.05	1369337	0.19	0	0	87659087	12.17
Unknown	35443	0	0	0	0	0	124331790	17.26	94460416	13.12
Total	96116100	13.35	25001193	3.47	94842994	13.17	296679047	41.19	425869070	59.13

Figure 1. Morphological characteristic of Aquilaria sinensis.

Click here to access/download;Figure;Figure 1.tif

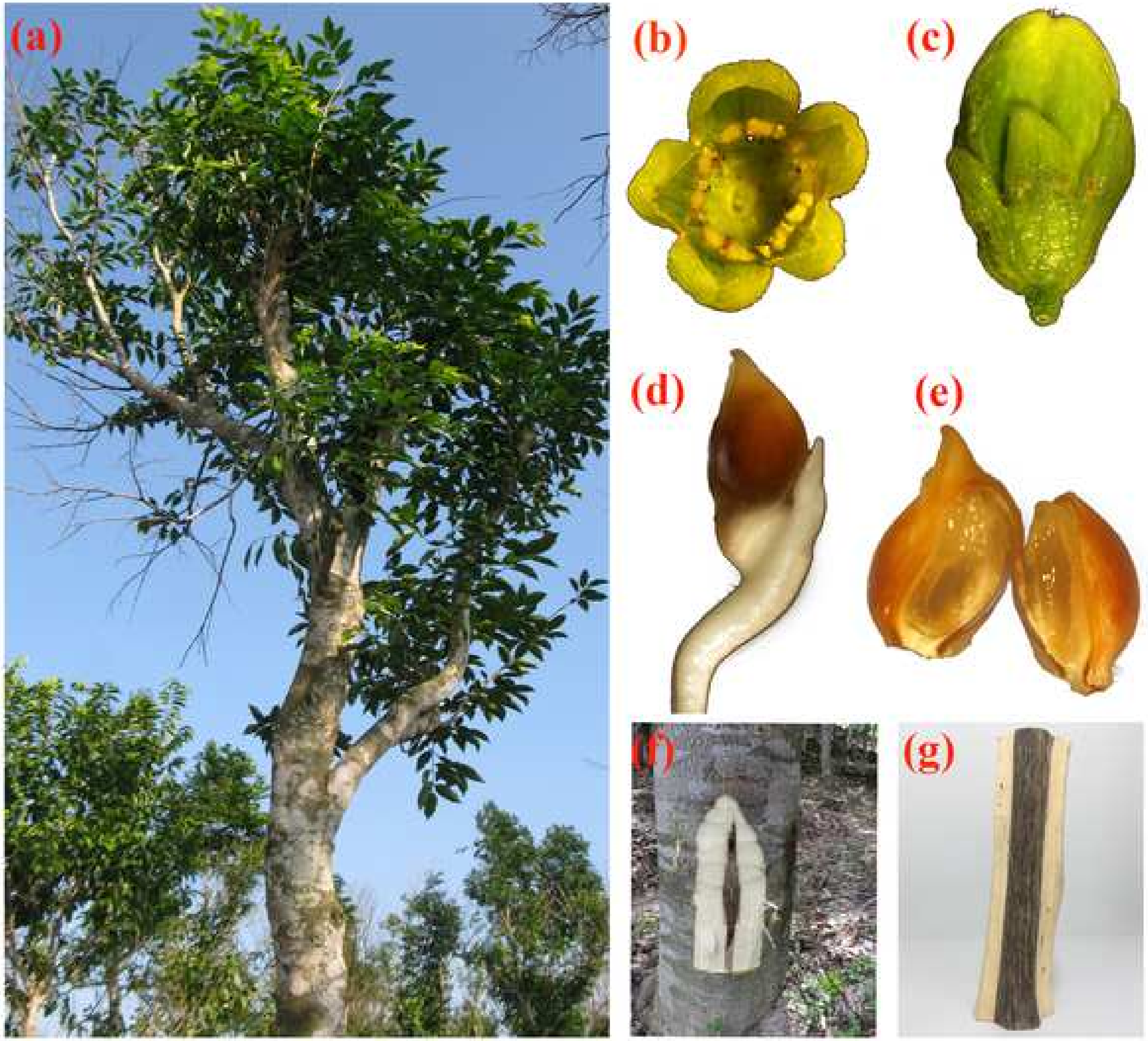

Figure 2. Hi-C interaction matrix for A.sinensis genome assembly with 8 clusters.

Click here to access/download;Figure;Figure 2.tif

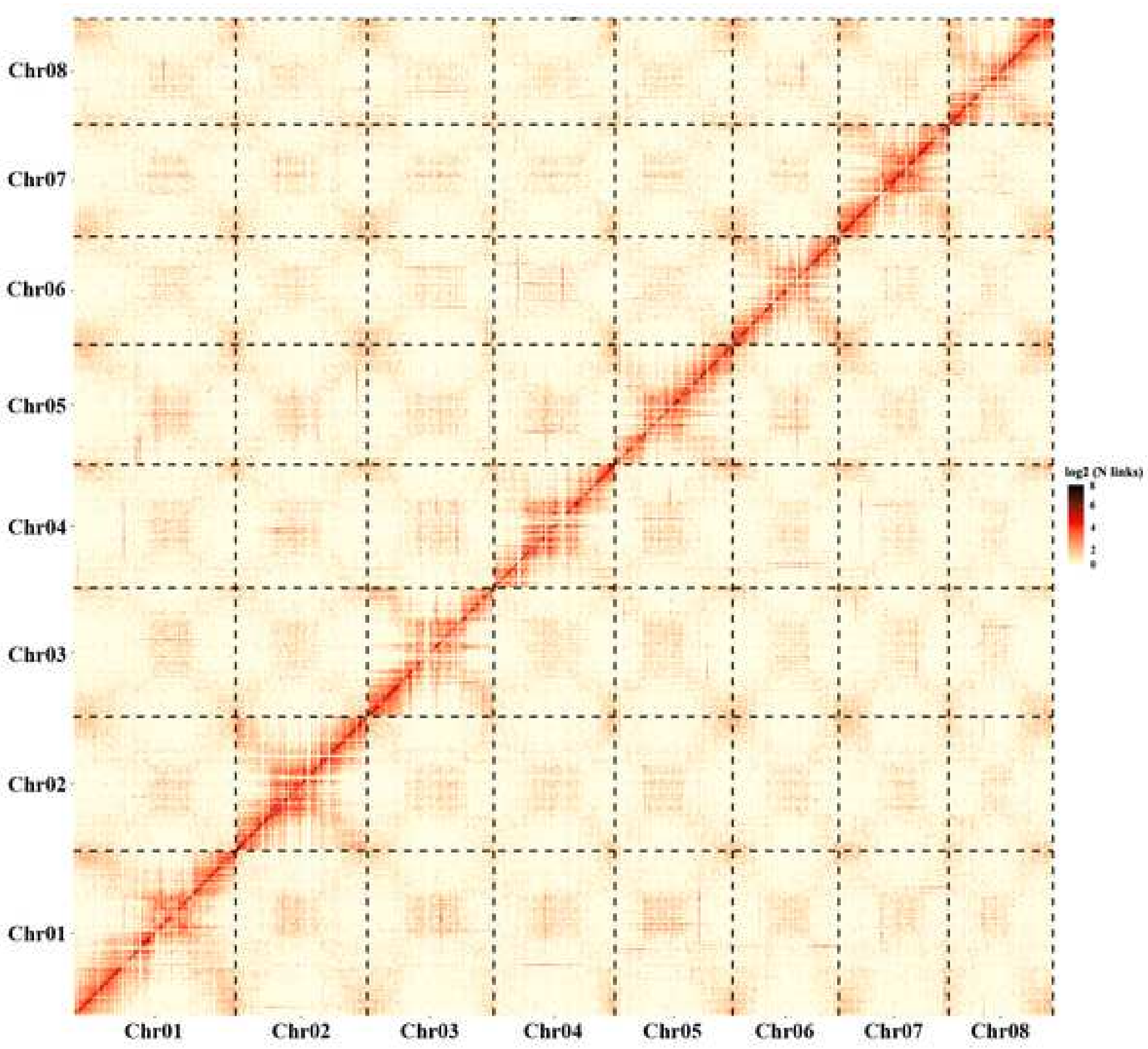

Figure 3. Comparative genomic analysis of Aquilaria sinensis and other plant species.

Click here to access/download;Figure;Figure.3.tif

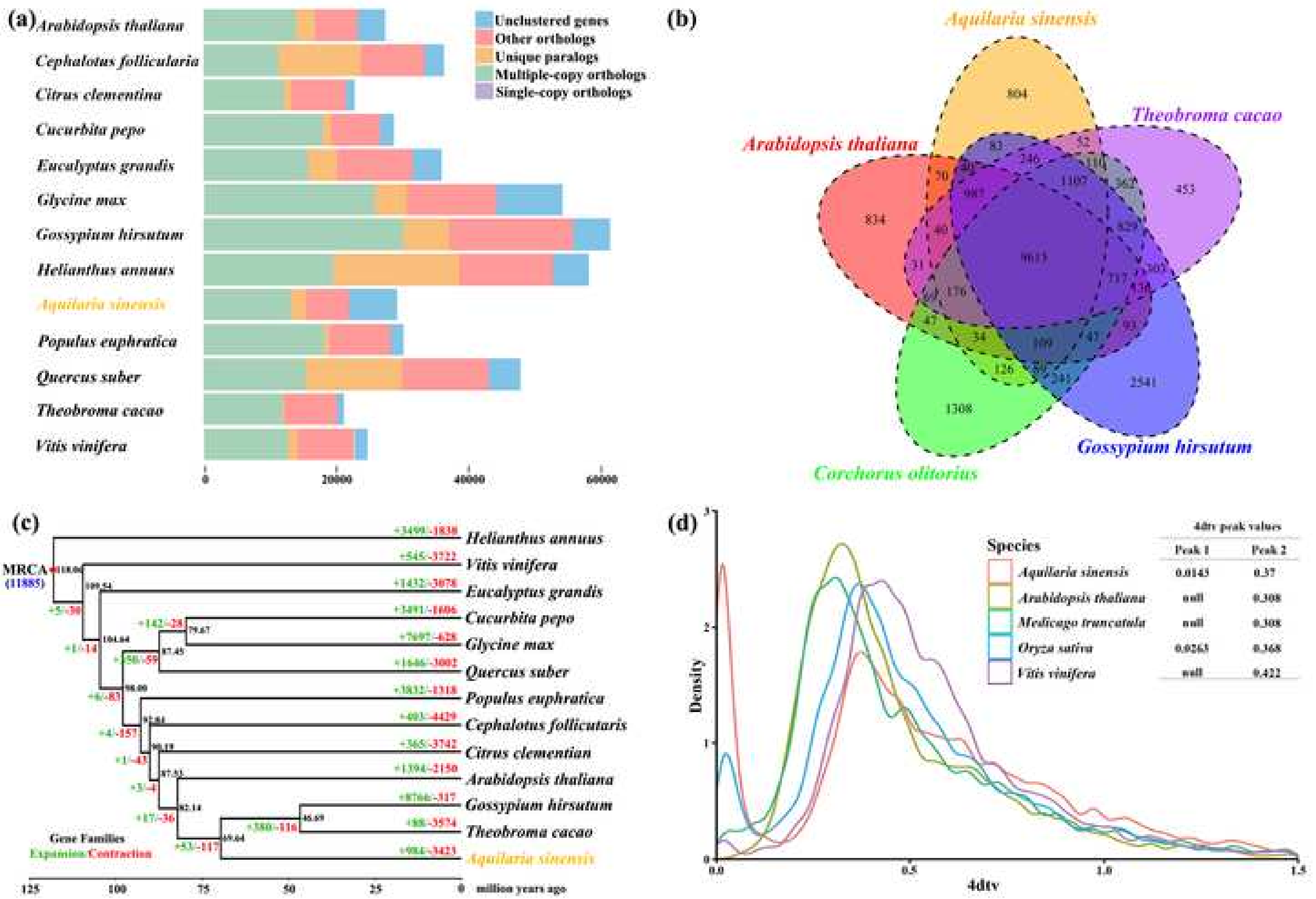

S Fig.1 K-mer (k = 19) analysis for estimating the size of the Aquilaria sinensis genome.

Click here to access/download
Supplementary Material
S Fig.1.tif

S Fig.2 GC content and average sequencing depth of the Illumina sequencing data used for genome estimation.

Click here to access/download
Supplementary Material
S Fig.2.tif

S Fig.3 Distribution of sequence divergence rates of different TE types with Repbase (A) and de nove (B) methods in Aquilaria

Click here to access/download
Supplementary Material
S Fig.3.tif

S Fig.4 Distribution of gene elements in Aquilaria sinensis genome and other six plant genome.

Click here to access/download
Supplementary Material
S Fig.4.tif

S Fig.5 Phylogenetic tree of 13 plant species including Aquilaria sinensis.

Click here to access/download
Supplementary Material
S Fig.5.tif

S Fig.6 Estimation of divergence time of 13 plant species investigated in the present study.

Click here to access/download
Supplementary Material
S Fig.6.tif

S Fig.7 GO enrichment of expansion gene families in Aquilaria sinensis genome.

Click here to access/download
Supplementary Material
S Fig.7.tif

S Fig.8 GO enrichment of contraction gene families in Aquilaria sinensis genome.

Click here to access/download
Supplementary Material
S Fig.8.tif

Click here to access/download
Supplementary Material
S Table1 - S Table 18.xls

Click here to access/download
Supplementary Material
Response to Reviewer #2.docx

Click here to access/download
Supplementary Material
Genome Scope and KAT.rar

Click here to access/download
Supplementary Material
Revised Manuscript with track changes.docx

 Click here to access/download;Personal Cover;Cover letter for revised manuscript.docx

Dear editor and reviewers:

Thanks for your letter and the reviewers' comments concerning our manuscript entitled "Genome sequence of agarwood tree *Aquilaria sinensis* (Lour.) Spreng: the first chromosome-level draft genome in the Thymelaeceae family" (ID: GIGA-S-19-00378). These comments are all valuable and very helpful for improving our paper. We have studied the comments carefully and have made correction which we hope meeting with approval. Especially we upload the relevant files as supplementary materials for your reference. Revised positions are marked in red in the manuscript with track changes. The main corrections in this revised manuscript and the responds to the reviewer comments are as the following:

Response to Reviewer #1:

Reviewer #1: The authors have responded to all my comments.

The methods and the results are well described. The new version of the article is greatly improved.

Thanks to the authors for the improvements.

Response: Thanks for your previous professional comments and recommendation.

Response to Reviewer #2

Reviewer #2: Your first revision of the Data Note presenting the genome sequence of the Agarwood really improved the quality of the manuscript addressing most of my questions and concerns. I would be very happy to recommend it for publication of the paper if you reconsider some sentences and add some extra information to the supplementary material. Specifically, would like you to:

1. Comment: Add the Genome scope plot and KAT stacked histogram obtained using k=27 to the supplementary material. In their response to comment #10 you just describe the results of the KAT stacked histogram, they sound good but I would like to take a look by myself.

Response: The genomes cope plot and KAT stacked histogram have been upload in the supplementary material named as Genome Scope and KAT.rar for your reference.

2. Comment: Regarding to answer number 4 to the other reviewer (reviewer 1). I cannot really distinguish the fields properly and compare the numbers. I would like you to mention the number of breaks during the Hi-C correction in the text and add a readable table to the supplementary material.

Response: Breaking number indicated by Hi-C correction were added in the Supplementary Table S6. We modified the original description in the text with total breaking numbers in the Line 298.

3. Comment: Thanks for the clarification about the Hi-C preprocessing pipeline. Please add the

reference to Servant et al. Genome Biology 2015. I cannot find it in the new revision.

Response: This reference has been added in the new revised manuscript.

4. Comment: Line 250 at page 9: I think "comparation" should be replaced by "comparison".

Response: This correction is accepted in the new revised manuscript

5. Comment: Please reconsider the sentence at line 309 in page 11. Once again, you are mixing the vulnerability status of the species with the severely endangered one to give emphasis to a threat to the conservation of the species and the utility of the genome for management of natural populations. I suggest they could write something like: "Considering that currently, natural *A.sinensis* tree populations in China(?) are highly threatened due to stem heavily exploited for creating costly agarwood products, the genome assembly of *A.sinensis* tree presented here will provide valuable information to aid the global conservation of these precious species and contribute to understanding the mechanism of the agarwood formation, eventually will help us reveal the evolution of aromatic genes and plants." I think this sentence it is clearer, reflecting your point about the current threats without over-stating the species conservation status.

Response: This well description is accepted in the new revised manuscript.

We tried our best to improve the manuscript and made some changes in the revised manuscript. These changes will not influence the content and framework of the paper. And here we did not list the changes but marked in red in revised paper. We appreciated for you and the reviews warm work earnestly, and hope that the correction will meet with approval. Once again, thanks for your comments and suggestion.

Sincerely,

Haofu Dai

Hainan Engineering Research Center of Agarwood, Institute of Tropical Bioscience and Biotechnology, Chinese Academy of Tropical Agricultural Sciences, Rd. Xueyuan No.4, Haikou, 571101, China

Tel: +86-898-6696-1869

Email: daihaofu@itbb.org.cn
