# Supplementary figures and images for: Genome sequence of the agarwood tree Aquilaria sinensis (Lour.) Spreng: the first chromosome-level draft genome in the Thymelaeceae family
Source: Gigascience. 2020 Mar 2;9(3):giaa013. doi: 10.1093/gigascience/giaa013 (PMC7050300; doi:10.1093/gigascience/giaa013)

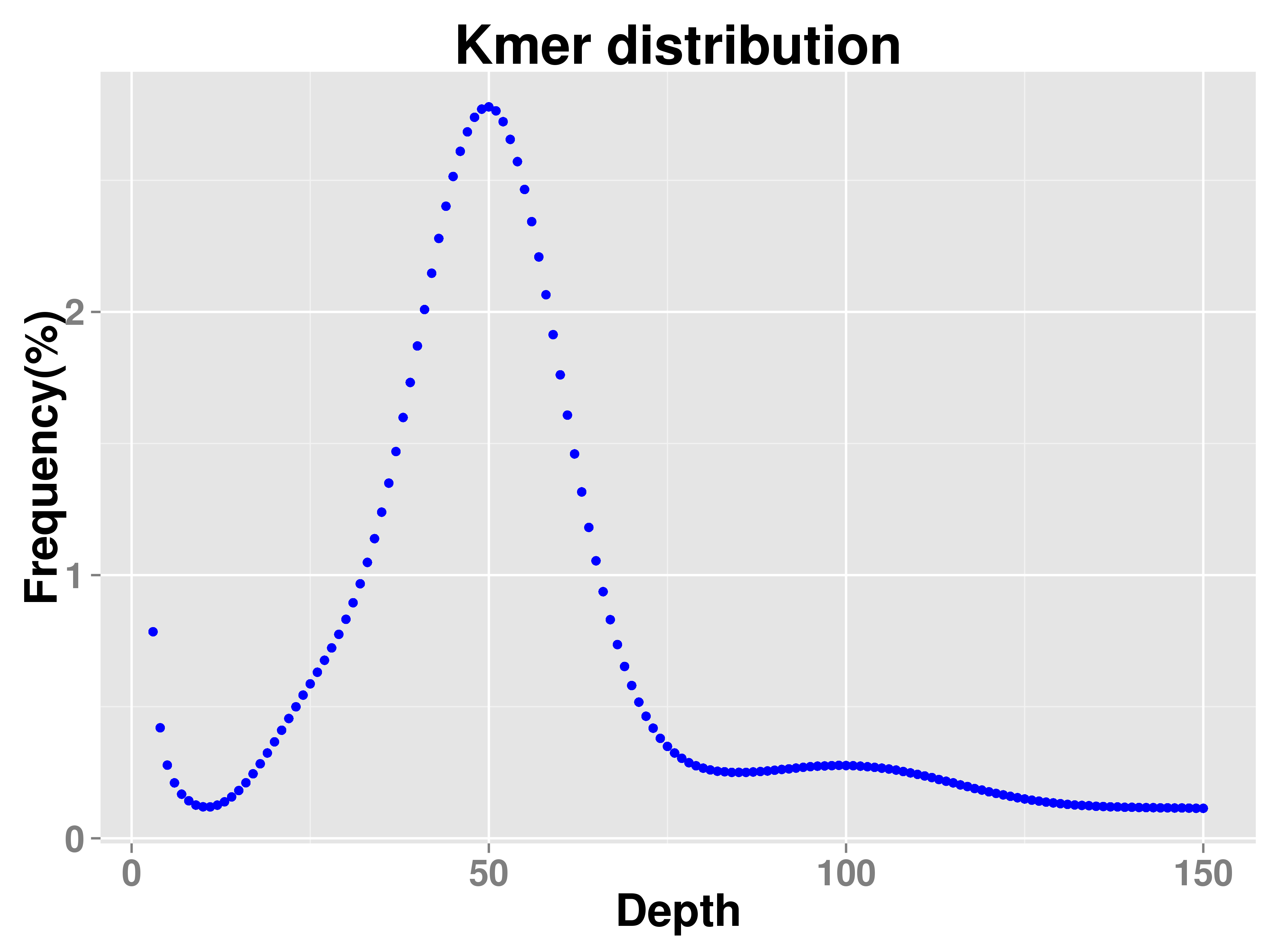

Supplement: giaa013_Supplemental_Files [file giaa013_supplemental_files.zip › S Fig.1.tif]

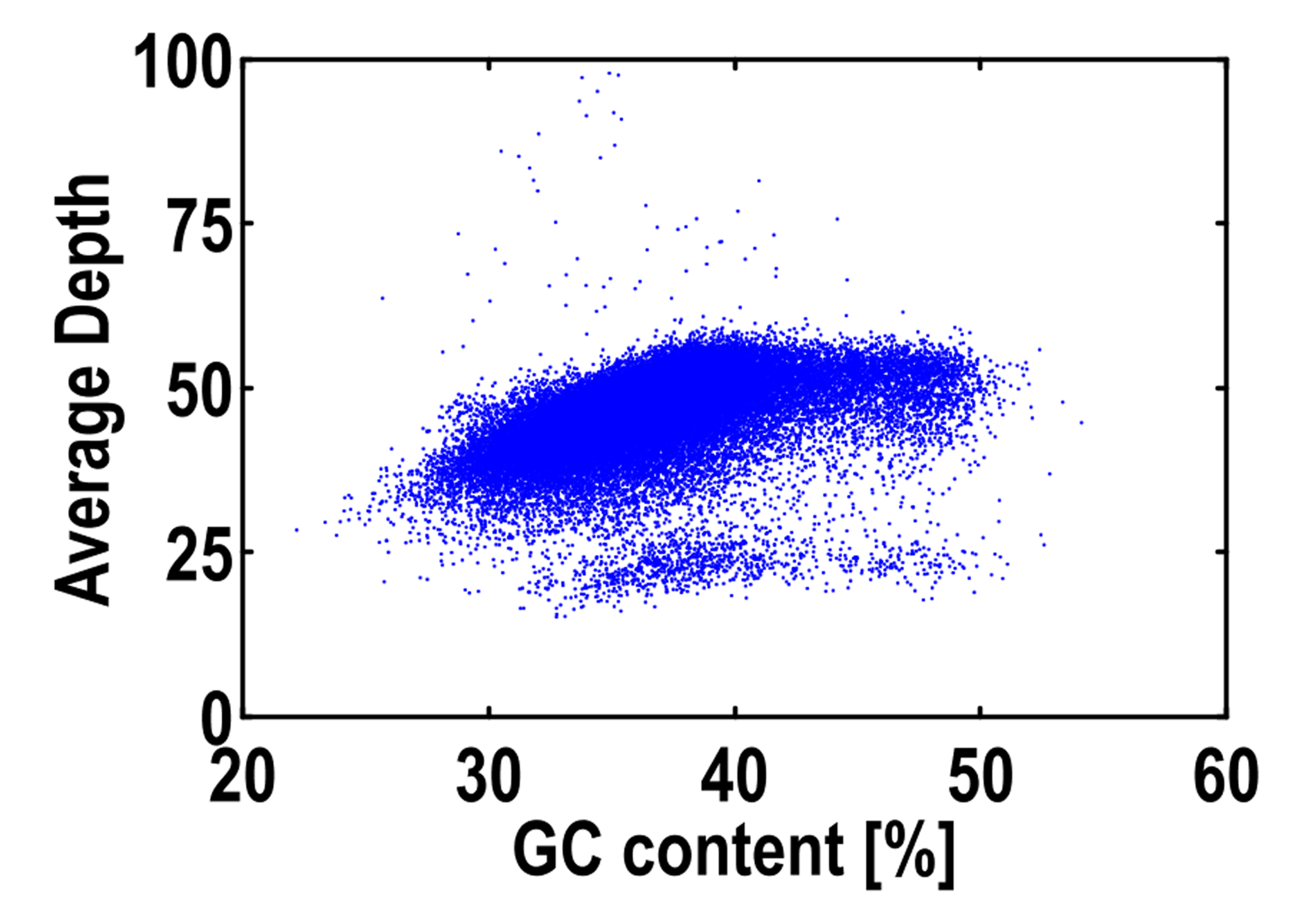

Supplement: giaa013_Supplemental_Files [file giaa013_supplemental_files.zip › S Fig.2.tif]

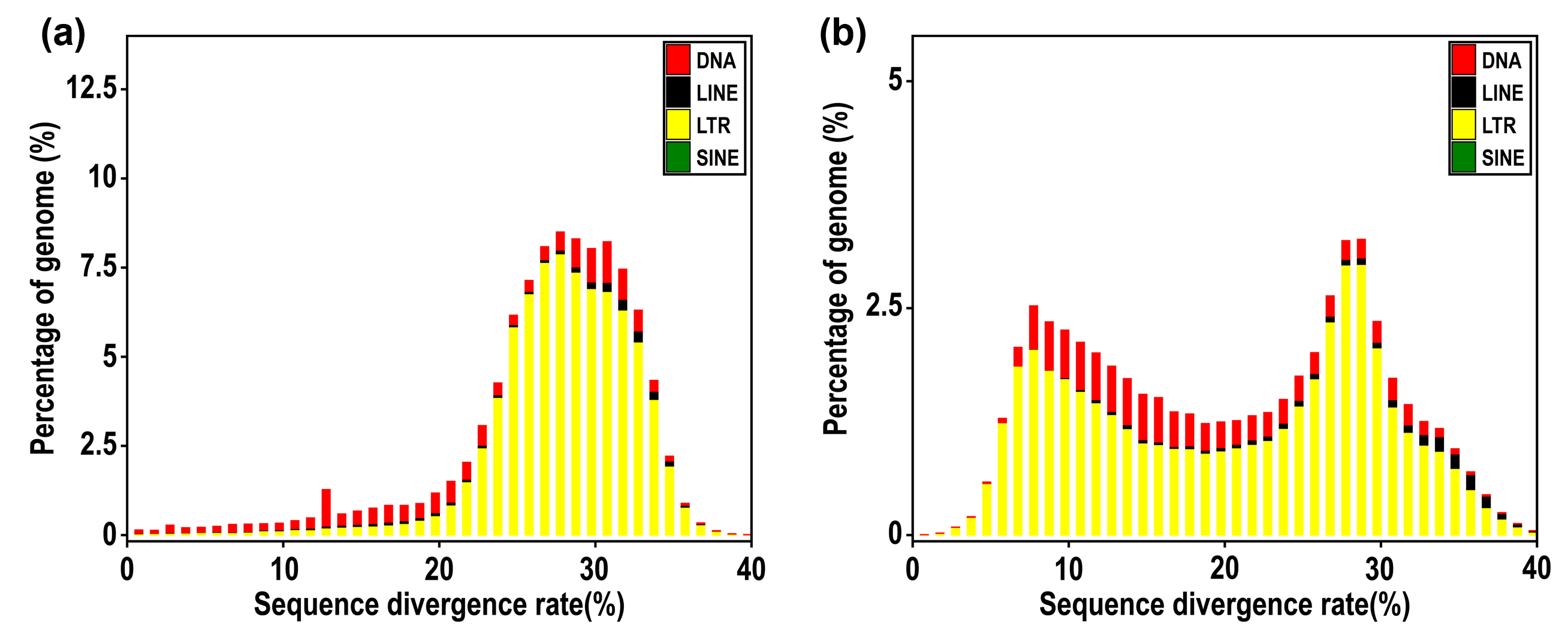

Supplement: giaa013_Supplemental_Files [file giaa013_supplemental_files.zip › S Fig.3.tif]

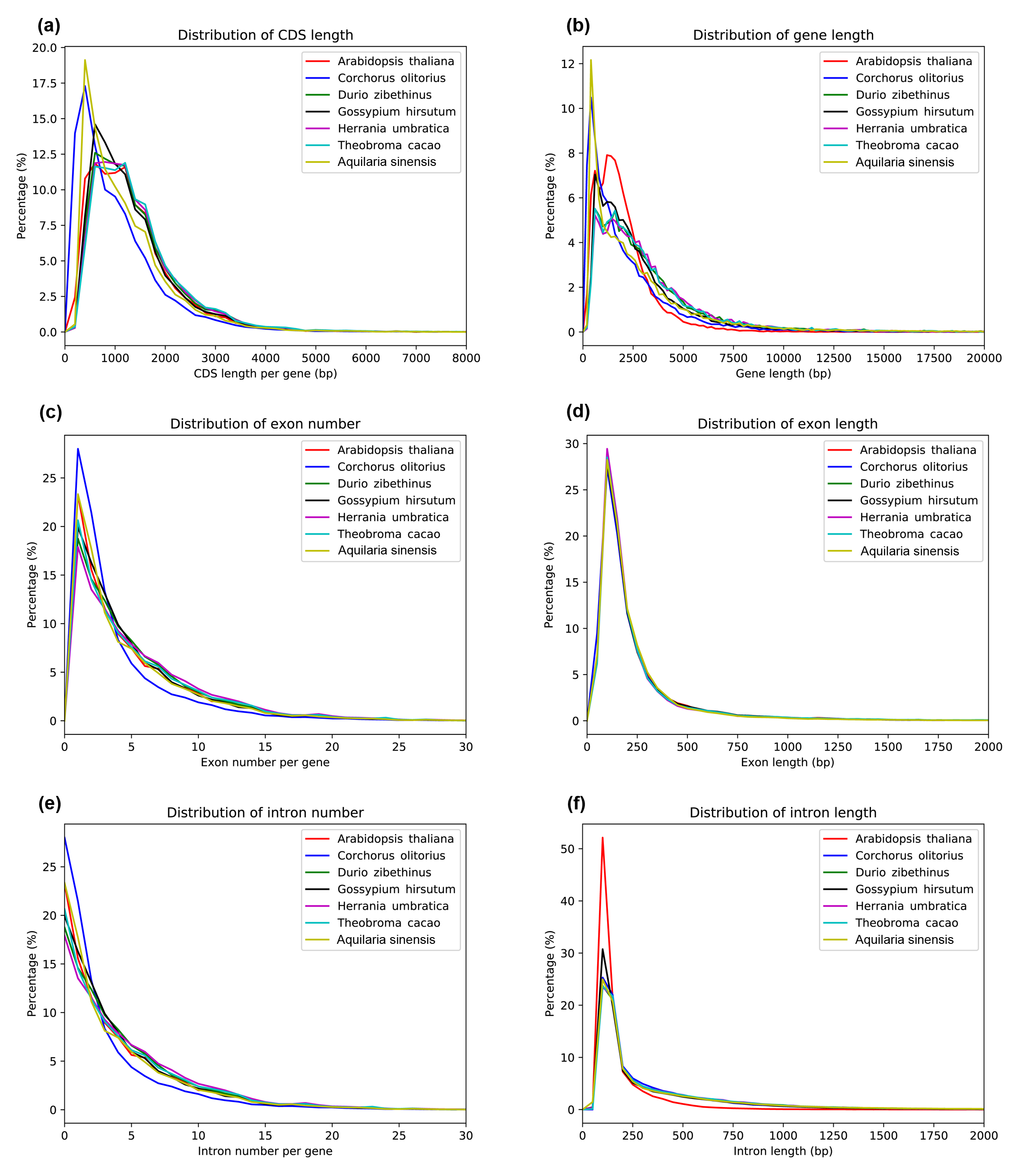

Supplement: giaa013_Supplemental_Files [file giaa013_supplemental_files.zip › S Fig.4.tif]

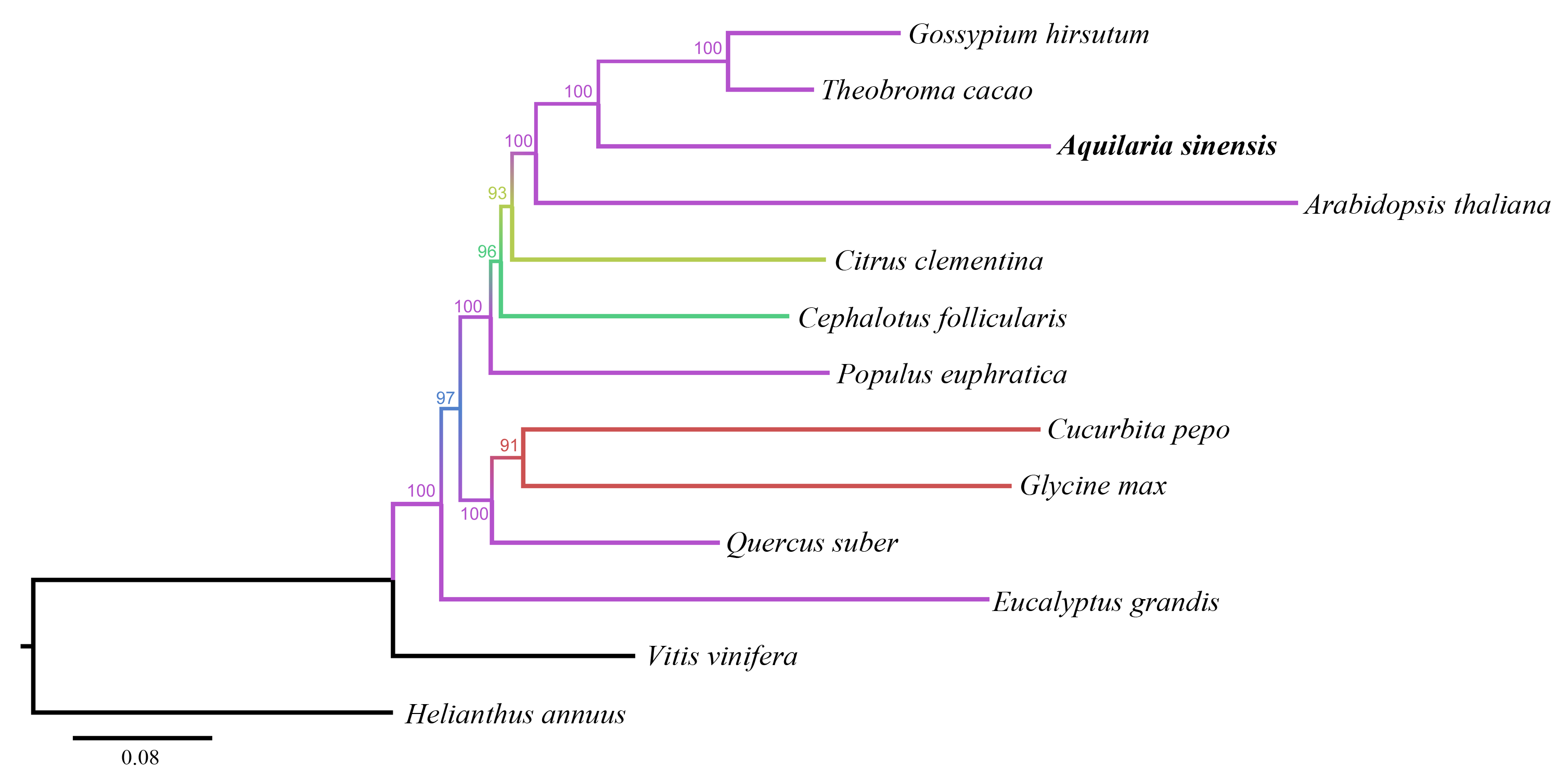

Supplement: giaa013_Supplemental_Files [file giaa013_supplemental_files.zip › S Fig.5.tif]

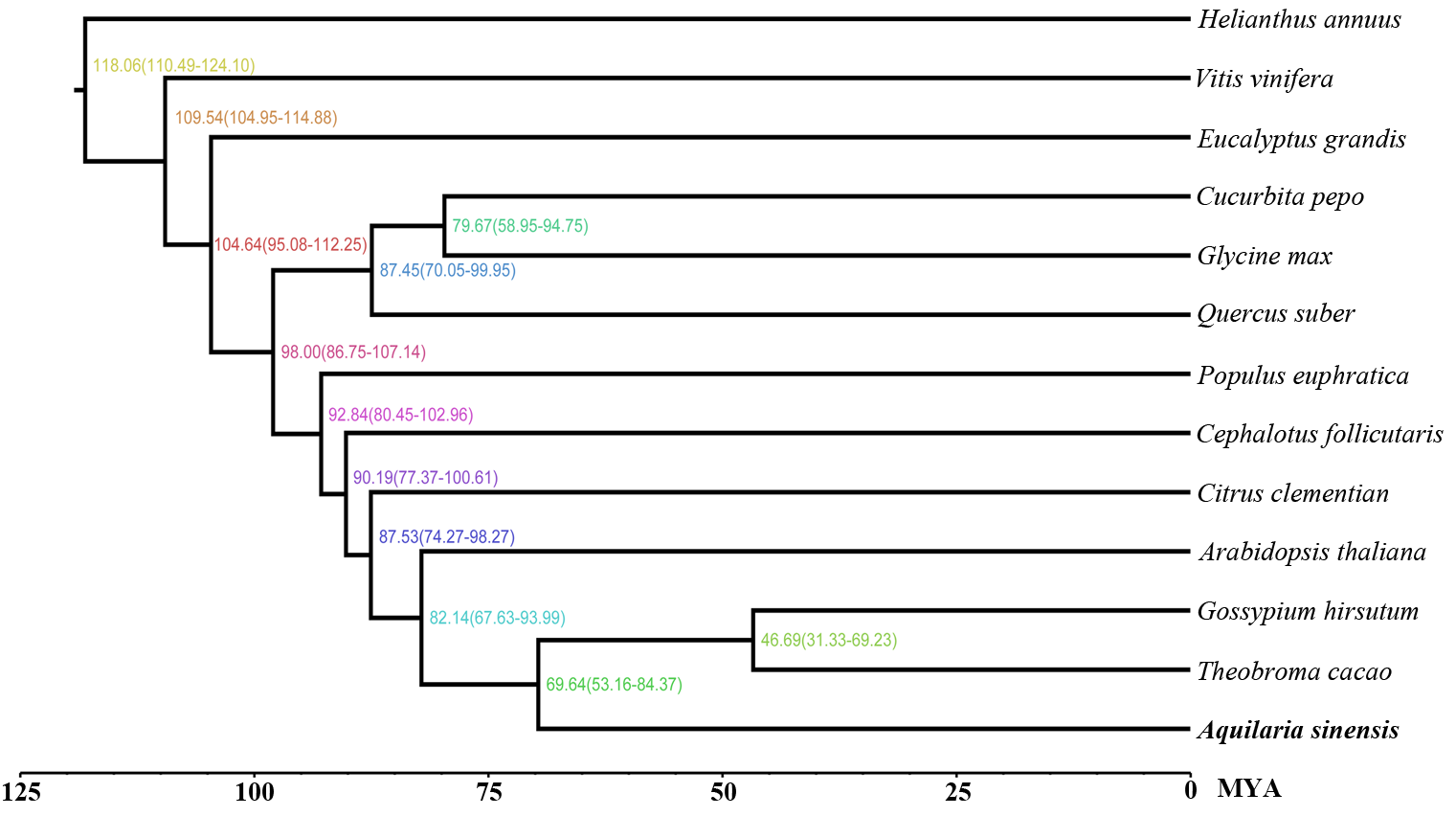

Supplement: giaa013_Supplemental_Files [file giaa013_supplemental_files.zip › S Fig.6.tif]

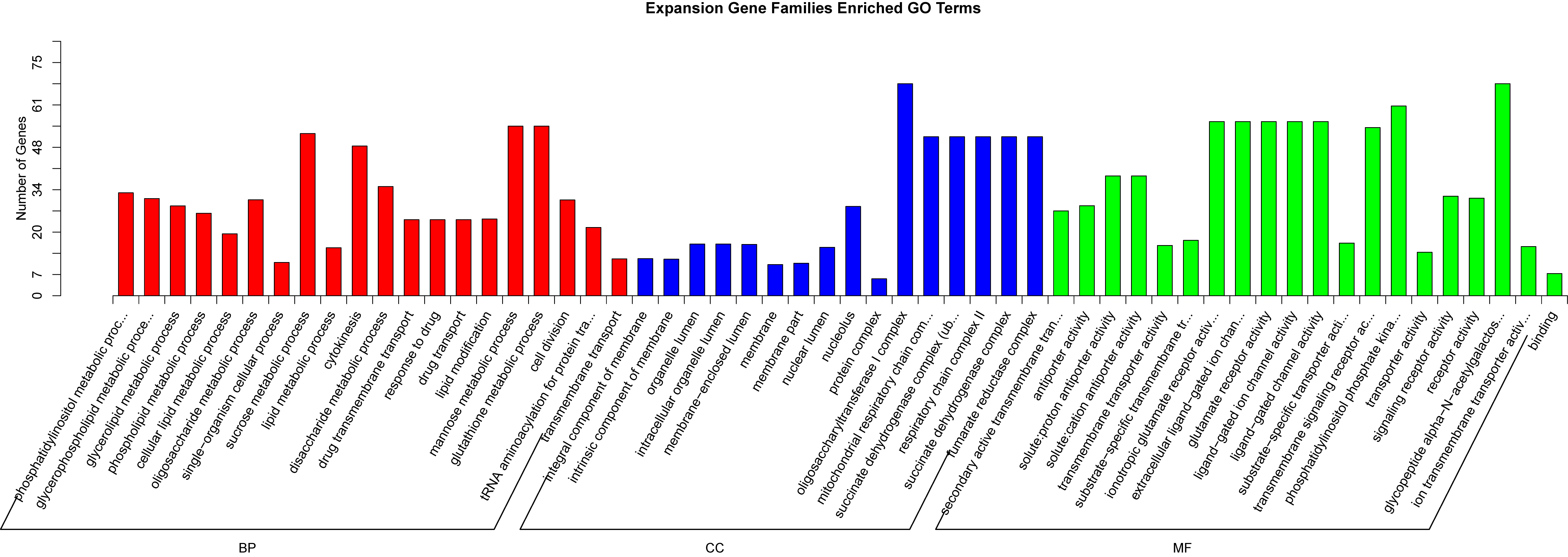

Supplement: giaa013_Supplemental_Files [file giaa013_supplemental_files.zip › S Fig.7.tif]

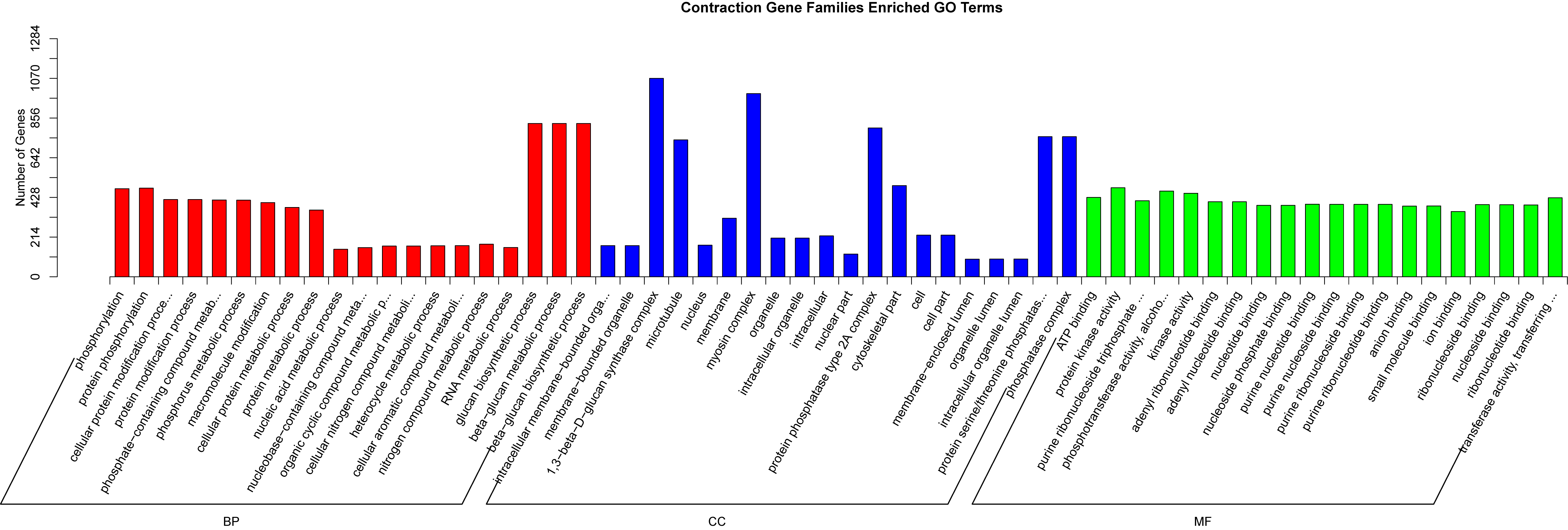

Supplement: giaa013_Supplemental_Files [file giaa013_supplemental_files.zip › S Fig.8.tif]
