# Supplementary material for: Genome sequence of the agarwood tree Aquilaria sinensis (Lour.) Spreng: the first chromosome-level draft genome in the Thymelaeceae family
Source: Gigascience. 2020 Mar 2;9(3):giaa013. doi: 10.1093/gigascience/giaa013 (PMC7050300; doi:10.1093/gigascience/giaa013)
Supplement: giaa013_Response_to_Reviewer_Comments_Original_Submission [file giaa013_response_to_reviewer_comments_original_submission.pdf]

Author's Response To Reviewer Comments

Close

Dear editor and reviewers:
Thanks for your letter and the reviewers' comments concerning our manuscript entitled "Genome sequence of agarwood tree Aquilaria sinensis (Lour.) Spreng: the first chromosome-level draft genome in the Thymelaeceae family" (ID: GIGA-S-19-00378). Those comments are all valuable and very helpful for revising and improving our paper, as well as the important guiding the significance to our future research. We have studied the comments carefully and have made correction which we hope meeting with approval. Especially we upload the relevant data to the NCBI-SRA and revised the language. Revised positions are marked in red in the manuscript with track changes. The main corrections in this revised manuscript and the responds to the reviewer comments are as the following and its word format (including table and figure) have been uploaded in the Supplementary materials too.

Response to the Reviewer #1:
Comment: The authors describe the first chromosome-level genome assembly of the agarwood tree. This work is of great interest because of the intensive use of the agar tree and the need of preservation of this tree. The authors have produced a big work to assemble and annotate the genome with good results.
1. Comment: The methods are well described but in line 204, the authors introduce RNA sequencing of mixed tissues. There is no description of the generation of those data (type of tissues used, RNA extraction method, RNA sequencing libraries preparation, sequencing depth ...). The data are not available in the NCBI Sequence Read Archive under the BioProject accession number given.
Response : The description of the RNA-seq data have been added and can be tracked with red marks in the revised manuscript from line 170 to line 179. Raw data have been submitted to PRJNA556948 in the SRA database of NCBI and its run number is SRR10276652.
2. Comment: The table 1 doesn't contain the statistics of the final assembly (same table than Supplementary table S3). What is the proportion of the gaps introduced after scaffolding with Hi-C?
Response : The new table 1 about the final assembly have been upload in the revised manuscript. The proportion of gaps is roughly 0.9% (~length of 6.4 Mb) in the final genome.
3. Comment: In the figure 3d, a table with the values of the picks could be added (minor issue).
Response : The values of the peaks have been added.
4. Comment: In the line 285, the authors used the HI-C data to correct the mis-assemblies. How many mis-assemblies were found? How have they been detected?
Response : In the initial Hi-C interactive heatmap, the regions of the interactive intensity between different pseudochromosomes higher than their intensity within same pseudochromosome were determined as the mis-assemblies. Then these regions were corrected based on the interactive intensities of re-clustered after the contig divided into the equal length of 100 kb. The following table is the comparison of initial Hi-C assembly and the correct Hi-C assembly:

Chr	Initial assembly		Final assembly	
	Size	Scaf Num	Size	Scaf Num
Chr01	114,318,616	151	109,870,270	166
Chr02	108,239,053	49	99,503,772	60
Chr03	94,462,133	70	89,391,083	91
Chr04	89,950,395	72	88,784,932	86
Chr05	85,082,375	76	86,380,100	112
Chr06	80,447,689	69	84,956,755	91
Chr07	76,713,504	87	80,601,762	89
Chr08	76,239,003	94	77,134,862	110
Total	725,452,768	668	716,623,536	805

5. Comment: Finally, the Hi-C sequencing data are not available in the NCBI Sequence Read Archive under the BioProject accession number given.
Response : The raw data of Hi-C sequencing have been submitted to PRJNA556948 in the SRA database of NCBI now and its run number is SRR10362483.

Response to Reviewer #2:
Comment: The genome sequence presented in your manuscript is a highly valuable resource for sustainable commercial exploitation and to help understanding the evolution of aromatic plants. You have reached a high level of continuity, obtainng a well-annotated chromosome-level assembly. In addition, the phylogenetic analysis is consistent with previous divergence time estimates and the analysis of gene families help to define the evolutionary context of the released the genome. However, before accepting the publication, I would like you to do a major revision of the manuscript and address some of my concerns.
1. Comment: In the background you state that A. sinensis have been included in the IUCN red list. Although that's true you don't mention the current assessment as VULNERABLE (by Harvey Brown, 2018). I think you should let this clear and be cautious at the end when you say it is extremely endangered, etc. Avoid overstatements, the species populations have suffered a dramatic decline in the last 10 years and wild populations are threatened, that's it.
Response：We have confirmed your information about the new assessment of A.sinensis and the description of endangered have been instead by “the species populations of A.sinensis have suffered a dramatic decline in the last 10 years and its wild populations are threatened” in the revision.
2. Comment: Please clarify which method you used to run the 19-mer analysis have you used your own method or software? Is it available for the community and reproducible?
Response：Using K-mer for estimating genome size is the routine approach based on sequencing data for estimating genomic characteristics. This GCE (genomic character estimator) was described in https://arxiv.org/abs/1308.2012 and adopted in other genome projects.
3. Comment: Regarding to this analysis how did you obtained the heterozygosity estimate of 0.32%? have you compared this with the results obtained with Genomescope, GCE or similar methods?
Response：The heterozygosity was estimated by GCE method with a Poisson model according to the same paper of genome size estimation. The heterozygosity value is various with different softwares. The Genome Scope used a mixed model and shown that the heterozygosity of A.sinensis is 0.6% and KAT show the number of 0.34%. We replaced the number with Genomescope result according comparative description in Genomescope’s paper
https://academic.oup.com/bioinformatics/article/33/14/2202/3089939.
4. Comment: I assume the HiSeq2500 was used to sequence two pair-end libraries and, by comparing sequence and k-mer coverage, the read length was 75bp. Please, specify if the library was paired or single end and what was the read length.
Response：The library was pair-end and its read length is 150 bp. This information has been revised in this current manuscript.
5. Comment: Line 122 - you wrote "We obtained 4.8 million subreads" however, nanopore produced long reads and the term subread is used only for pacbio technology. I think this is wrong and should be corrected.
Response：The subreads is instead by nanopore long reads.
6. Comment: Nanopore sequence data: I would also like to know what was the mean accuracy of the reads. The reads that pass the filter are the ones having an average quality of at least 7, right?
Response：It is right, the mean qscore is greater than 7 (>7).
7. Comment: 133 - well, the comparison with other genomes looks very good. However, I suggest adding some comparison to the contig-level assembly for A.sinensis obtained with Pacbio (GCA_005392925.1 SCBG_Asin_1.0) and also A.agallocha (GCA_000696445.1). Mentioning these assemblies will look fair and also will highlight the higher contiguity of the one you present here.
Response：Two contig level assemblies for Aquilaria species have been detailed in the Table S4. The assembly derived from PacBio platform have not been published and the specific details could be got from the NCBI, so we just list N50 of contig in the compared table.
8. Comment: P.129. while polishing with pilon you haven't try to fix indels, why is this? Knowing that nanopore have recurrent errors in homopolymer tracks, I think will be an obvious thing to do. I am curious to know the reason.
Response: In Pilon software, the parameter of “fix bases” included the snps and indels. We specified it in the revised manuscript and the fix parameter has been listed as following:

Pilon --fix parameter：
--fix fixlist
A comma-separated list of categories of issues to try to fix:
"snps": try to fix individual base errors;
"indels": try to fix small indels;

"bases": shorthand for "snps" and "indels" (for back compatibility);
9. Comment: Regarding to the preprocessing of Hi-C reads. Does Fastp merely remove the adaptors? Other protocols, such as Arima Hi-C Mapping pipeline, try to remove some sequence at the ligation junction and keep the 5' ends to avoid the inclusion of "chimeric" reads…
Response：Fastp removed low quality reads and adaptors. For "chimeric" reads, this Hi-C pipeline is built on the basis of Hi-C pro software. Hi-C pro can detect all types of reads and get valid reads for downstream Hi-C analysis.
10. Comment: The assembly has a fairly high gene completeness as estimated by BUSCO. The mapping rate of the pair end reads reflects sequence quality. However, I would like you to perform some additional evaluations of the assembly such as a KAT stacked histogram, that reflect the efficiency and completeness of the assembly process, having into account the genome architecture (https://kat.readthedocs.io/en/latest/).
Response： The estimated assembly completeness is 90.08% in following 27-mer stacked histogram by KAT tools. Black square labeled the distribution of k-mers present in illumine paired-end data but absent in the assembly. Red labeled K-mers present in the read set and once in the assembly. Mainly of black fall into the regions of low quality and heterozygous (half of homozygous peak in x-axis).
11. Comment: I would also like you to include a link to the assembly and annotation in a public repository (this will be very important for the scientific community). In fact, I would like to have a look at the genome myself. In the manuscript you merely give access to the raw reads.
Response：The assembly, annotation and other related files of A.sinensis genome have been uploaded to GigaDB private FTP area for reviewers within peer review stage. The Gigascience will release them to GigaDB if the manuscript were published. Actually, we are constructing a professional public repository about Aquilaria species, including their genomics, transcriptome and metabolism, especially for their natural products. We will let you know when our database was online in the future.
12. Comment: In the supplementary table 2: what are exactly "properly mapped" reads? Do you mean "properly paired"? After looking at ENA it seems that the illlumina is a single end library…I am a bit confused with the term "properly mapped".
Response：The illumine sequence was based on the paired-end library, we have sent this mistake to the submission staff of NCBI for correction.
13. Comment: Please correct some typos: you wrote "Predication" several times (line 193, line 216…) but the correct word is PREDICTION, (i.e. Gene Prediction). Also "sinenensis" (instead of "sinensis") or "de nove" (instead of de novo) appear later.
Response：These typos have been corrected in the revised manuscript.
14. Comment: Sentence in lines 227-235 will read much better by starting with "After removing the redundancy caused by alternative splicing variations and retaining the longest transcript of each gene, whole protein coding genes sets….OrthoMCL…[64]."
Response：This good suggestion has been adopted in the revised manuscript.
15. Comment: Please revise the English of this last pages (lines 238-296,) (specially for the last two pages, that seem to have been written on a rush)
Response：These paragraphs were revised in the revised manuscript.
16. Comment: Line 246 Gblocks filters "poorly aligned or highly divergent sites" from the alignment. I found "bias(ed) regions", as you wrote, a bit weird.
Response：We have accepted this more professional description in the revised manuscript.
17. Comment: Rather than contraction and expansion families I suggest to call them "contracted" and "expanded)
Response：All the contraction and expansion before families or gene families were replaced by contracted or expanded in the revised manuscript.
18. Comment: YOU SHOULD IMPROVE THE IMAGE RESOLUTION OF THE FIGURES the image resolution of the Figures in the manuscript (quality is very poor and numbers are often hard to read) What happened to Figure3c? in fact the supplementary images have much better quality.
Response：The image resolution of all the original figures in manuscript is 508 ×508 dpi. When they were inserted into word (.doc), their definition in generating PDF were much better than coalescing them directly after the manuscript.
19. Comment: I found the Whole genome duplication analyses weak and speculative. To my knowledge the 4DTV ratios can be used to date some evolutionary events, such as hybridization of gene family expansions/contractions. In your case the closest species to A. sinensis have very different genome sizes, with cocoa being almost half of sinensis (~300Mb) and Cotton with almost 3 times the sinensis genome size 2.2Gb. Have you found evidences of a ~300Mb of the agarwood genome being duplicated? Unless you have a more clear analysis of genome size evolution I suggest you to remove this from the manuscript?
Response：As your suggestion, we narrow down this section to “4DTv analysis” and shown the peak

value of distribution. A.sinensis is the only species with chromosome-level assembly in Familia Thymelaeaceae. The cross-Familia comparison cannot fully reveal the diversity in plant genome size.

20. Comment: It would be good if you compare the divergence time estimates you have obtained with those found by other authors. By checking www.timetree.org I have confirmed that your estimate lay between the Confidence Intervals obtained by comparing several studies. It makes sense, to discuss a little bit your results or at least say that they are in concordance with previous studies.

Response：The divergence time of Asterids and Rosids, G.hisutum and T.cacao obtained in our analysis were compare with the previous studies about calibration divergence time in the revised manuscript.

21. Comment: A question about the biology of the plant. In your analyses, have you found any relationship between the immune genes and those involved in the production of agarwood or aromatic resin?

Response：This good suggestion might guide the significance to our future research. Immune gene is very important for plant defense response. But actually, we are focused on the genes about the terpene and flavonoid synthesis in the current stage, especially about the chromone synthesis, which is the sign matters for agarwood formation in A.sinensis. In addition, plant defense related genes were also concerned in our studies, the genes involved in abscisic acid and salicylic acid synthesis and regulation were correlative with contents of chromone in regions of agarwood formation. The ABA and SA also could induced the agarwood formation after they were injected into the stem of A.sinensis plants. According to the quantity, NB-LRRs and WD-40 were frequently presented in the DEG and annotation files. The expression trends of some NB-LRRs and WD-40 genes were highly correlative with agarwood formation based on time gradient. The pattern mentioned above from our previous transcriptome dataset without biological replicates (just five stages and each stage with mixture samples. because the RNA in the stem of agarwood formation was hardly accumulated), only part of them were verified with qPCR. Out further effort will focus on the LCM-based transcriptomics study.

We tried our best to improve the manuscript and made some changes in the revised manuscript. These changes will not influence the content and framework of the paper. And here we did not list the changes but marked in red in revised paper. We appreciated for you and the reviews warm work earnestly, and hope that the correction will meet with approval. Once again, thanks for your comments and suggestion.

Sincerely,

Haofu Dai

Hainan Engineering Research Center of Agarwood, Institute of Tropical Bioscience and Biotechnology, Chinese Academy of Tropical Agricultural Sciences, Rd. Xueyuan No.4, Haikou, 571101, China
Tel: +86-898-6696-1869
Email: daihaofu@itbb.org.cn

Close
