# Supplementary material for: Genome sequence of the agarwood tree Aquilaria sinensis (Lour.) Spreng: the first chromosome-level draft genome in the Thymelaeceae family
Source: Gigascience. 2020 Mar 2;9(3):giaa013. doi: 10.1093/gigascience/giaa013 (PMC7050300; doi:10.1093/gigascience/giaa013)
Supplement: giaa013_Response_to_Reviewer_Comments_Revision_1 [file giaa013_response_to_reviewer_comments_revision_1.pdf]

Author's Response To Reviewer Comments

Close

Dear editor and reviewers:
Thanks for your letter and the reviewers' comments concerning our manuscript entitled "Genome sequence of agarwood tree Aquilaria sinensis (Lour.) Spreng: the first chromosome-level draft genome in the Thymelaeceae family" (ID: GIGA-S-19-00378). These comments are all valuable and very helpful for improving our paper. We have studied the comments carefully and have made correction which we hope meeting with approval. Especially we upload the relevant files as supplementary materials for your reference. Revised positions are marked in red in the manuscript with track changes. The main corrections in this revised manuscript and the responds to the reviewer comments are as the following:

Response to Reviewer #1:
Reviewer #1: The authors have responded to all my comments.
The methods and the results are well described. The new version of the article is greatly improved.
Thanks to the authors for the improvements.
Response: Thanks for your previous professional comments and recommendation.

Response to Reviewer #2
Reviewer #2: Your first revision of the Data Note presenting the genome sequence of the Agarwood really improved the quality of the manuscript addressing most of my questions and concerns. I would be very happy to recommend it for publication of the paper if you reconsider some sentences and add some extra information to the supplementary material. Specifically, would like you to:
1. Comment: Add the Genome scope plot and KAT stacked histogram obtained using k=27 to the supplementary material. In their response to comment #10 you just describe the results of the KAT stacked histogram, they sound good but I would like to take a look by myself.
Response: The genomes cope plot and KAT stacked histogram have been upload in the supplementary material named as Genome Scope and KAT.rar for your reference.
2. Comment: Regarding to answer number 4 to the other reviewer (reviewer 1). I cannot really distinguish the fields properly and compare the numbers. I would like you to mention the number of breaks during the Hi-C correction in the text and add a readable table to the supplementary material.
Response: Breaking number indicated by Hi-C correction were added in the Supplementary Table S6. We modified the original description in the text with total breaking numbers in the Line 298.
3. Comment: Thanks for the clarification about the Hi-C preprocessing pipeline. Please add the reference to Servant et al. Genome Biology 2015. I cannot find it in the new revision.
Response: This reference has been added in the new revised manuscript.
4. Comment: Line 250 at page 9: I think "comparation" should be replaced by "comparison".
Response: This correction is accepted in the new revised manuscript
5. Comment: Please reconsider the sentence at line 309 in page 11. Once again, you are mixing the vulnerability status of the species with the severely endangered one to give emphasis to a threat to the conservation of the species and the utility of the genome for management of natural populations. I suggest they could write something like: "Considering that currently, natural A.sinensis tree populations in China(?) are highly threatened due to stem heavily exploited for creating costly agarwood products, the genome assembly of A.sinensis tree presented here will provide valuable information to aid the global conservation of these precious species and contribute to understanding the mechanism of the agarwood formation, eventually will help us reveal the evolution of aromatic genes and plants." I think this sentence it is clearer, reflecting your point about the current threats without over-stating the species conservation status.
Response: This well description is accepted in the new revised manuscript.

We tried our best to improve the manuscript and made some changes in the revised manuscript. These changes will not influence the content and framework of the paper. And here we did not list the changes but marked in red in revised paper. We appreciated for you and the reviews warm work earnestly, and hope that the correction will meet with approval. Once again, thanks for your comments and suggestion.

Sincerely,

Haofu Dai

Hainan Engineering Research Center of Agarwood, Institute of Tropical Bioscience and Biotechnology, Chinese Academy of Tropical Agricultural Sciences, Rd. Xueyuan No.4, Haikou, 571101, China
Tel: +86-898-6696-1869
Email: daihaofu@itbb.org.cn

Close
