# Supplementary material for: Genome sequence of the agarwood tree Aquilaria sinensis (Lour.) Spreng: the first chromosome-level draft genome in the Thymelaeceae family
Source: Gigascience. 2020 Mar 2;9(3):giaa013. doi: 10.1093/gigascience/giaa013 (PMC7050300; doi:10.1093/gigascience/giaa013)
Supplement: giaa013_Reviewer_1_Report_Original_Submission — Caroline Belser -- 9/5/2019 Reviewed [file giaa013_reviewer_1_report_original_submission.pdf]

Reviewer Report

Title: Genome sequence of agarwood tree Aquilaria sinensis (Lour.) Spreng: the first chromosome-level draft genome in the Thymelaeceae family

Version: Original Submission **Date:** 9/5/2019

Reviewer name: Caroline Belser

Reviewer Comments to Author:

The authors describe the first chromosome-level genome assembly of the agarwood tree. This work is of great interest because of the intensive use of the agar tree and the need of preservation of this tree. The authors have produced a big work to assemble and annotate the genome with good results.
The methods are well described but in line 204, the authors introduce RNA sequencing of mixed tissues. There is no description of the generation of those data (type of tissues used, RNA extraction method, RNA sequencing libraries preparation, sequencing depth ...). The data are not available in the NCBI Sequence Read Archive under the BioProject accession number given.
The table 1 doesn't contain the statistics of the final assembly (same table than Supplementary table S3). What is the proportion of the gaps introduced after scaffolding with Hi-C?
In the figure 3d, a table with the values of the picks could be added (minor issue).
In the line 285, the authors used the HI-C data to correct the mis-assemblies. How many mis-assemblies were found? How have they been detected?
Finally, the Hi-C sequencing data are not available in the NCBI Sequence Read Archive under the BioProject accession number given.
Caroline Belser

Level of Interest

Please indicate how interesting you found the manuscript: Choose an item.

Quality of Written English

Please indicate the quality of language in the manuscript: Choose an item.

Declaration of Competing Interests

Please complete a declaration of competing interests, considering the following questions:

- Have you in the past five years received reimbursements, fees, funding, or salary from an organisation that may in any way gain or lose financially from the publication of this manuscript, either now or in the future?

- Do you hold any stocks or shares in an organisation that may in any way gain or lose financially from the publication of this manuscript, either now or in the future?
- Do you hold or are you currently applying for any patents relating to the content of the manuscript?
- Have you received reimbursements, fees, funding, or salary from an organization that holds or has applied for patents relating to the content of the manuscript?
- Do you have any other financial competing interests?
- Do you have any non-financial competing interests in relation to this paper?

If you can answer no to all of the above, write 'I declare that I have no competing interests' below. If your reply is yes to any, please give details below.

I declare that I have no competing interests

I agree to the open peer review policy of the journal. I understand that my name will be included on my report to the authors and, if the manuscript is accepted for publication, my named report including any attachments I upload will be posted on the website along with the authors' responses. I agree for my report to be made available under an Open Access Creative Commons CC-BY license (http://creativecommons.org/licenses/by/4.0/). I understand that any comments which I do not wish to be included in my named report can be included as confidential comments to the editors, which will not be published.

Choose an item.

To further support our reviewers, we have joined with Publons, where you can gain additional credit to further highlight your hard work (see: https://publons.com/journal/530/gigascience). On publication of this paper, your review will be automatically added to Publons, you can then choose whether or not to claim your Publons credit. I understand this statement.

Yes Choose an item.
