# Supplementary material for: Genome sequence of the agarwood tree Aquilaria sinensis (Lour.) Spreng: the first chromosome-level draft genome in the Thymelaeceae family
Source: Gigascience. 2020 Mar 2;9(3):giaa013. doi: 10.1093/gigascience/giaa013 (PMC7050300; doi:10.1093/gigascience/giaa013)
Supplement: giaa013_Reviewer_2_Report_Original_Submission — Fernando Cruz -- 9/29/2019 Reviewed [file giaa013_reviewer_2_report_original_submission.pdf]

Reviewer Report

Title: Genome sequence of agarwood tree Aquilaria sinensis (Lour.) Spreng: the first chromosome-level draft genome in the Thymelaeceae family

Version: Original Submission **Date:** 9/29/2019

Reviewer name: Fernando Cruz

Reviewer Comments to Author:

The genome sequence presented in your manuscript is a highly valuable resource for sustainable commercial exploitation and to help understanding the evolution of aromatic plants. You have reached a high level of continuity, obtainng a well-annotated chromosome-level assembly. In addition, the phylogenetic analysis is consistent with previous divergence time estimates and the analysis of gene families help to define the evolutionary context of the released the genome. However, before accepting the publication, I would like you to do a major revision of the manuscript and address some of my concerns.

1. In the background you state that A. sinensis have been included in the IUCN red list. Although that's true you don't mention the current assessment as VULNERABLE (by Harvey Brown, 2018). I think you should let this clear and be cautious at the end when you say it is extremely endangered, etc. Avoid overstatements, the species populations have suffered a dramatic decline in the last 10 years and wild populations are threatened, that's it.
2. Please clarify which method you used to run the 19-mer analysis have you used your own method or software? Is it available for the community and reproducible?
3. Regarding to this analysis how did you obtained the heterozygosity estimate of 0.32%? have you compared this with the results obtained with Genomescope, GCE or similar methods?
4. I assume the HiSeq2500 was used to sequence two pair-end libraries and, by comparing sequence and k-mer coverage, the read length was 75bp. Please, specify if the library was paired or single end and what was the read length.
5. Line 122 - you wrote "We obtained 4.8 million subreads" however, nanopore produced long reads and the term subread is used only for pacbio technology. I think this is wrong and should be corrected.
6. Nanopore sequence data: I would also like to know what was the mean accuracy of the reads. The reads that pass the filter are the ones having a average quality of at least 7, right?
7. 133 - well, the comparison with other genomes looks very good. However, I suggest adding some comparison to the contig-level assembly for A. sinensis obtained with Pacbio (GCA_005392925.1 SCBG_Asin_1.0) and also A. agallocha (GCA_000696445.1). Mentioning these assemblies will look fair and also will highlight the higher contiguity of the one you present here.
8. P.129. while polishing with pilon you haven't try to fix indels, why is this? Knowing that nanopore have recurrent errors in homopolymer tracks, I think will be an obvious thing to do. I am curious to know the reason.
9. Regarding to the preprocessing of Hi-C reads. Does Fastp merely remove the adaptors? Other protocols, such as Arima Hi-C Mapping pipeline, try to remove some sequence at the ligation junction

and keep the 5' ends to avoid the inclusion of "chimeric" reads…

10. The assembly has a fairly high gene completeness as estimated by BUSCO. The mapping rate of the pair end reads reflects sequence quality. However, I would like you to perform some additional evaluations of the assembly such as a KAT stacked histogram, that reflect the efficiency and completeness of the assembly process, having into account the genome architecture (https://kat.readthedocs.io/en/latest/)

11. I would also like you to include a link to the assembly and annotation in a public repository (this will be very important for the scientific community). In fact, I would like to have a look at the genome myself. In the manuscript you merely give access to the raw reads.

12. In the supplementary table 2: what are exactly "properly mapped" reads? Do you mean "properly paired"? After looking at ENA it seems that the illlumina is a single end library…I am a bit confused with the term "properly mapped".

13. Please correct some typos: you wrote "Predication" several times (line 193, line 216…) but the correct word is PREDICTION, (i.e. Gene Prediction). Also "sinenensis" (instead of "sinensis") or "de nove" (instead of de novo) appear later.

14. Sentence in lines 227-235 will read much better by starting with "After removing the redundancy caused by alternative splicing variations and retaining the longest transcript of each gene, whole protein coding genes sets….OrthoMCL…[64]."

15. Please revise the English of this last pages (lines 238-296,) (specially for the last two pages, that seem to have been written on a rush)

16. Line 246 Gblocks filters "poorly aligned or highly divergent sites" from the alignment. I found "bias(ed) regions", as you wrote, a bit weird.

17. Rather than contraction and expansion families I suggest to call them "contracted" and "expanded)

18. YOU SHOULD IMPROVE THE IMAGE RESOLUTION OF THE FIGURES the image resolution of the Figures in the manuscript (quality is very poor and numbers are often hard to read) What happened to Figure3c? in fact the supplementary images have much better quality.

19. I found the Whole genome duplication analyses weak and speculative. To my knowledge the 4DTV ratios can be used to date some evolutionary events , such as hybridization of gene family expansions/contractions. In your case the closest species to A. sinensis have very different genome sizes, with cocoa being almost half of sinensis (~300Mb) and Cotton with almost 3 times the sinensis genome size 2.2Gb. Have you found evidences of a ~300Mb of the agarwood genome being duplicated? Unless you have a more clear analysis of genome size evolution I suggest you to remove this from the manuscript?

20. It would be good if you compare the divergence time estimates you have obtained with those found by other authors. By checking www.timetree.org I have confirmed that your estimate lay between the Confidence Intervals obtained by comparing several studies. It makes sense, to discuss a little bit your results or at least say that they are in concordance with previous studies.

21. A question about the biology of the plant. In your analyses, have you found any relationship between the immune genes and those involved in the production of agarwood or aromatic resin?

Level of Interest

Please indicate how interesting you found the manuscript: Choose an item.

Quality of Written English

Please indicate the quality of language in the manuscript: Choose an item.

Declaration of Competing Interests

Please complete a declaration of competing interests, considering the following questions:

- Have you in the past five years received reimbursements, fees, funding, or salary from an organisation that may in any way gain or lose financially from the publication of this manuscript, either now or in the future?
- Do you hold any stocks or shares in an organisation that may in any way gain or lose financially from the publication of this manuscript, either now or in the future?
- Do you hold or are you currently applying for any patents relating to the content of the manuscript?
- Have you received reimbursements, fees, funding, or salary from an organization that holds or has applied for patents relating to the content of the manuscript?
- Do you have any other financial competing interests?
- Do you have any non-financial competing interests in relation to this paper?

If you can answer no to all of the above, write 'I declare that I have no competing interests' below. If your reply is yes to any, please give details below.

I declare that I have no competing interests

I agree to the open peer review policy of the journal. I understand that my name will be included on my report to the authors and, if the manuscript is accepted for publication, my named report including any attachments I upload will be posted on the website along with the authors' responses. I agree for my report to be made available under an Open Access Creative Commons CC-BY license (http://creativecommons.org/licenses/by/4.0/). I understand that any comments which I do not wish to be included in my named report can be included as confidential comments to the editors, which will not be published.

Choose an item.

To further support our reviewers, we have joined with Publons, where you can gain additional credit to further highlight your hard work (see: https://publons.com/journal/530/gigascience). On publication of

this paper, your review will be automatically added to Publons, you can then choose whether or not to claim your Publons credit. I understand this statement.

Yes Choose an item.
