# Supplementary material for: Genome sequence of the agarwood tree Aquilaria sinensis (Lour.) Spreng: the first chromosome-level draft genome in the Thymelaeceae family
Source: Gigascience. 2020 Mar 2;9(3):giaa013. doi: 10.1093/gigascience/giaa013 (PMC7050300; doi:10.1093/gigascience/giaa013)
Supplement: giaa013_Reviewer_2_Report_Revision_1 — Fernando Cruz -- 1/9/2020 Reviewed [file giaa013_reviewer_2_report_revision_1.pdf]

**Reviewer Report**

**Title: Genome sequence of agarwood tree Aquilaria sinensis (Lour.) Spreng: the first chromosome-level draft genome in the Thymelaeceae family**

**Version: Revision 1** **Date:** 1/9/2020

**Reviewer name: Fernando Cruz**

**Reviewer Comments to Author:**

Your first revision of the Data Note presenting the genome sequence of the Agarwood really improved the quality of the manuscript addressing most of my questions and concerns. I would be very happy to recommend it for publication of the paper if you reconsider some sentences and add some extra information to the supplementary material. Specifically, would like you to:

1. Add the Genomescope plot and KAT stacked histogram obtained using k=27 to the supplementary material. In their response to comment #10 you just describe the results of the KAT stacked histogram, they sound good but I would like to take a look by myself.
2. Regarding to answer number 4 to the other reviewer (reviewer 1). I cannot really distinguish the fields properly and compare the numbers. I would like you to mention the number of breaks during the Hi-C correction in the text and add a readable table to the supplementary material.
3. Thanks for the clarification about the Hi-C preprocessing pipeline. Please add the reference to Servant et al. Genome Biology 2015. I cannot find it in the new revision.
4. Line 250 at page 9: I think "comparation" should be replaced by "comparison".
5. Please reconsider the sentence at line 309 in page 11. Once again, you are mixing the vulnerability status of the species with the severely endangered one to give emphasis to a threat to the conservation of the species and the utility of the genome for management of natural populations. I suggest they could write something like: "Considering that currently, natural A.sinensis tree populations in China(?) are highly threatened due to stem heavily exploited for creating costly agarwood products, the genome assembly of A.sinensis tree presented here will provide valuable information to aid the global conservation of these precious species and contribute to understanding the mechanism of the agarwood formation, eventually will help us reveal the evolution of aromatic genes and plants." I think this sentence it is clearer, reflecting your point about the current threats without over-stating the species conservation status.

If you address these minor concerns your manuscript will be in great shape for publication and sharing the resource with the scientific community.

**Level of Interest**

Please indicate how interesting you found the manuscript: Choose an item.

**Quality of Written English**

Please indicate the quality of language in the manuscript: Choose an item.

**Declaration of Competing Interests**

Please complete a declaration of competing interests, considering the following questions:

- Have you in the past five years received reimbursements, fees, funding, or salary from an organisation that may in any way gain or lose financially from the publication of this manuscript, either now or in the future?
- Do you hold any stocks or shares in an organisation that may in any way gain or lose financially from the publication of this manuscript, either now or in the future?
- Do you hold or are you currently applying for any patents relating to the content of the manuscript?
- Have you received reimbursements, fees, funding, or salary from an organization that holds or has applied for patents relating to the content of the manuscript?
- Do you have any other financial competing interests?
- Do you have any non-financial competing interests in relation to this paper?

If you can answer no to all of the above, write 'I declare that I have no competing interests' below. If your reply is yes to any, please give details below.

I declare that I have no competing interests

I agree to the open peer review policy of the journal. I understand that my name will be included on my report to the authors and, if the manuscript is accepted for publication, my named report including any attachments I upload will be posted on the website along with the authors' responses. I agree for my report to be made available under an Open Access Creative Commons CC-BY license (http://creativecommons.org/licenses/by/4.0/). I understand that any comments which I do not wish to be included in my named report can be included as confidential comments to the editors, which will not be published.

Choose an item.

To further support our reviewers, we have joined with Publons, where you can gain additional credit to further highlight your hard work (see: https://publons.com/journal/530/gigascience). On publication of this paper, your review will be automatically added to Publons, you can then choose whether or not to claim your Publons credit. I understand this statement.

Yes Choose an item.
